# An Unusual and Facile Synthetic Route to Alumoles

**DOI:** 10.1002/anie.202000899

**Published:** 2020-04-23

**Authors:** Jiancheng Li, Peng Wu, Wenjun Jiang, Bin Li, Binju Wang, Hongping Zhu, Herbert W. Roesky

**Affiliations:** ^1^ State Key Laboratory of Physical Chemistry of Solid Surface National Engineering Laboratory for Green Chemical Productions of Alcohols-Ethers-Esters College of Chemistry and Chemical Engineering Xiamen University Xiamen Fujian 361005 China; ^2^ Institüt für Anorganische Chemie Universität Göttingen Tammannstrasse 4 37077 Göttingen Germany; ^3^ State Key Laboratory of Structural Chemistry Fujian Institute of Research on the Structure of Matter Chinese Academy of Science Fuzhou Fujian 350002 China

**Keywords:** aggregation-induced emission, aluminum, heterocycles, alumoles, tris(pentafluorophenyl)borane

## Abstract

Reaction of the aluminum dialkynyl LAl(CCR)_2_ (L=N,N‐chelate ligand and R=organic group) with B(C_6_F_5_)_3_ proceeds through an intermediate with Al⋅⋅⋅η^2^‐C≡C side‐on coordination to form the alumoles (**2**, **4**, **6**). A distinctive reaction pattern indicates a new facile synthetic route to aluminum‐containing heterocycles. The synthetic process is described, and the characterization of compounds and computational calculations were carried out. Furthermore, alumoles **2** and **4** exhibit an aggregation‐induced emission (AIE) of the bright yellow fluorescence.

## Graphical abstract

**Al in**: Reaction of the aluminum dialkynyl with B(C_6_F_5_)_3_ proceeds through an intermediate with Al⋅⋅⋅η^2^‐C≡C side‐on coordination to form the alumole. Some of the alumoles synthesized in this way exhibit an aggregation‐induced emission (AIE) bright yellow fluorescence.

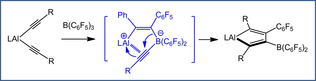

Aluminacyclopentadiene, named as the alumole, exhibits four dominantly localized π electrons within the AlC_4_ five‐membered ring. The alumole is an important organoaluminum species with an unique aluminum containing heterocycle, which usually emerges as an intermediate in organic or organometallic reactions.^1^ To date, only a few examples of alumoles were prepared from the salt metathesis reactions using 1,4‐dilithio‐1,3‐butadiene and aluminum dihalides as well as its derivatives.^2^ Previously an alternative method was applied using zirconacyclo‐pentadiene mediated transformation of internal alkyne and aluminum dihalides in a catalytic fashion.^3^ To our knowledge, no other route is available. Actually, the aluminum center of compounds exhibits strong Lewis acidity, and this causes problems for predicting a reliable synthetic route to prepare aluminum containing heterocycles. We have utilized the chelate ligand to coordinately stabilize the Al center and successfully prepared the aluminacyclopropenes L^1^Al(η^2^‐C_2_R_2_) (L^1^=CH(CMeNAr)_2_, Ar=2,6‐*i*Pr_2_C_6_H_3_; R=H, Ph, SiMe_3_, Me).^4^ Other organic heterocycles of composition AlC_2_N,^5^ AlC_3_,^6^ AlC_3_O,4a AlC_3_N,4a, 7 and Al_2_C_3_S^8^ have been reported. However, attempts to synthesize alumoles by alkyne‐insertion reactions were not successful.4b, 9 Herein, we explored the reaction of the aluminum dialkynyl LAl(C≡CR)_2_ with the strongly Lewis acidic borane B(C_6_F_5_)_3_. We were able to approach the alumole LAl{C(R)=C(C_6_F_5_)C[B(C_6_F_5_)_2_]=C(R)}, through the zwitterionic aluminum cation borate intermediate LAlC(R)=C(C_6_F_5_)B(C_6_F_5_)_2_(C≡CR) (see Scheme 2). The utilization of the strong Lewis acid enabled chemical changes of the two C≡C bonds at the Al center in an unusual fashion. Moreover, the present alumoles are highly substituted, featuring one C_6_F_5_ group and one B(C_6_F_5_)_2_ group besides two R groups attached at the butadiene skeleton. This is quite different from those alumoles having the same four R groups, showing expansion of the precursors. Herein we present in detail the formation of the alumoles with their intermediates.

We prepared three β‐diketiminato ligand‐stabilized aluminum dialkynyls L^1^Al(C≡CR)_2_ (L^1^=CH(MeCNAr)_2_, Ar=2,6‐*i*Pr_2_C_6_H_3_, R=Ph (**1**), *t*Bu (**1 a**), SiMe_3_ (**1 b**) by the metathesis reaction using L^1^AlCl_2_ and in situ generated LiC≡CR.^10^ Further reaction was carried out initially employing **1 a** and/or **1 b** with B(C_6_F_5_)_3_ 11 in a 1:1 molar ratio in C_6_D_6_. But, no reaction occurred even when treated under reflux conditions (see Figures S2‐1 and S2‐2 in the supporting information). It was fortunate that **1** reacted with B(C_6_F_5_)_3_ at 65 °C to smoothly afford alumole **2**, which was isolated as an orange solid in 78 % yield (Scheme 1). We have tried an alternative route using BEt_3_ instead of B(C_6_F_5_)_3_, but no reaction occurred with **1**, **1 a** and **1 b**, respectively.

**Scheme 1:**
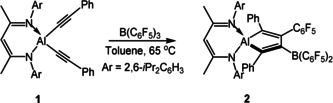
Reactions of aluminum dialkynyl compounds supported by β‐diketiminato ligands with B(C_6_F_5_)_3_ to form the alumole **2**.

To further expand the reaction scope, we selected an anilido‐imino ligand. We prepared the corresponding aluminum dialkynyls L^2^Al(C≡CR)_2_ (L^2^=*o*‐C_6_H_4_(CH=NAr)NAr, Ar=2,6‐*i*Pr_2_C_6_H_3_; R=Ph (**3**), 2‐thienyl (**5**)) by a similar route to L^1^Al(C≡CR)_2_ as mentioned before. Subsequently, the reactions of **3** or **5** with equivalent amounts of B(C_6_F_5_)_3_ were conducted under similar conditions in toluene. As a consequence, alumoles **4** and **6** were successfully prepared with a yield of 81 % and 86 %, respectively (Scheme 2).

**Scheme 2:**
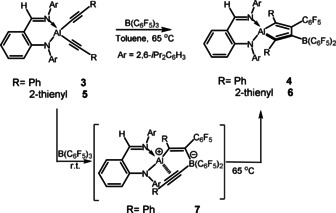
Reactions of aluminum dialkynyl compounds supported by anilido‐imino ligands with B(C_6_F_5_)_3_ to form the alumoles **4**, **6** via intermediate **7**.

X‐ray crystallographic analysis revealed that all **2**, **4** and **6**, contain the AlC_4_‐fused heterocycle (Figure 1 for **4**; Figure S3‐1 for **2**; Figure S3‐2 for **6**). The C(1)‐C(2) and C(3)‐C(4) bond lengths fall in the range of 1.354(3)–1.367(2) Å, while the C(2)‐C(3) bond lengths are 1.503(3)–1.524(2) Å, indicating the typical alumole structure exhibiting a double‐single‐double bond arrangement. It is important to mention that the AlC_4_‐rings in these three compounds are perfectly planar as indicated by the least square plane values of 0.0050 for **2**, 0.0442 for **4** and 0.0121 Å for **6**, respectively, which are below the standard of 0.05 Å. These central alumole frameworks are highly substituted, with two R groups at the two α‐C atoms, C_6_F_5_ and B(C_6_F_5_)_2_ group at each of the two β‐C atoms, and additionally the Al atom is chelated by the β‐diketiminato ligand for **2** and anilido‐imino ligand for **4** and **6**. In the ^13^C NMR spectrum of **2**, the C_4_ skeleton carbon resonances were found at 136.2, 157.3, 170.0, 170.4 ppm, and approximative chemical shift were also observed in that of **4** and **6** (see Supporting Information). Furthermore, the ^19^F NMR spectra display two sets of the fluorine resonances due to the C_6_F_5_ and B(C_6_F_5_)_2_ groups.

**Figure 1:**
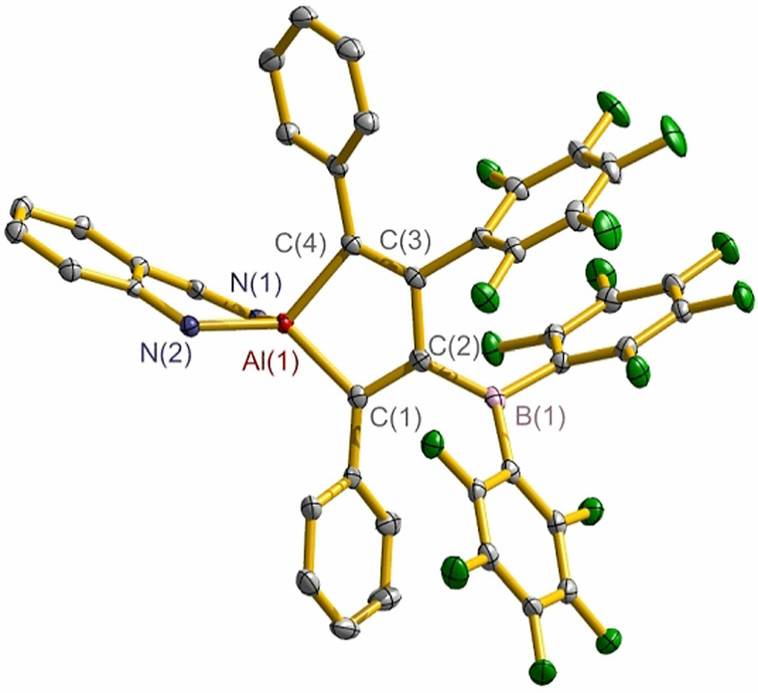
X‐ray crystal structure of **4** with thermal ellipsoids set at 20 % probability. H atoms and 2,6‐*i*Pr_2_C_6_H_3_ groups at N atoms are omitted for clarity. Selected bond lengths [Å] and angles [°]: Al(1)–N(1) 1.9460(15), Al(1)–N(2) 1.8626(15), Al(1)–C(1) 1.9886(18), Al(1)–C(4) 1.9872(18), C(1)–C(2) 1.367(2), C(2)–C(3) 1.524(2), C(3)–C(4) 1.357(3), B(1)–C(2) 1.552(3); N(1)‐Al(1)‐N(2) 95.53(6), C(1)‐Al(1)‐C(4) 90.60(7). The CCDC numbers for all the structures reported herein can be found in the Supporting Information.

To reveal the reaction mechanism in detail, we carried out the reaction of **3** with equivalent amounts of B(C_6_F_5_)_3_ by monitoring the NMR spectra. The time‐dependent ^1^H NMR spectra recorded in C_6_D_6_ at 65 °C are shown in Figure 2 (the corresponding ^19^F NMR spectra data is shown in Figure S2‐3. Clearly, there displays a gradual change of the proton resonances of either the *H*C=N in the skeleton or the C*H*Me_2_ in the Ar substituent. This shows the consumption of starting material **3** and the generation of product **4** via one species **7** which resonates at *δ*_*H*C=N_ 7.88 ppm and *δ*_C*H*Me2_ 2.41, 2.93, 2.99 and 4.06 ppm. We were intrigued with the composition and structure of **7** as the possible intermediate. By means of the reaction temperature (at room temperature) and time (5 h) control, we were able to crystallize an aluminum cation borate zwitterion **7** (light‐yellow crystals, yield of 53 %). However, the isolation of **7**‐like species from the reaction of either **1** or **5** with B(C_6_F_5_)_3_ was not successful. Furthermore, we prepared the amidinato ligand stabilized aluminum dialkynyl L^3^Al(C≡CPh)_2_ (L^3^=C*t*Bu(NCy)_2_, Cy=*cyclo*‐C_6_H_11_, **8**) and subsequently accomplished the reaction with B(C_6_F_5_)_3_. As a consequence, we isolated intermediate **9** (colorless crystals, yield of 67 %, Scheme 3). Unexpectedly compound **9** is remarkably stable, and its further conversion to alumole was not detected even at higher temperature (110 °C) for 24 h. This could be due to the much higher energy barrier (39.6 kcal mol^−1^) for the reaction of the second alkynyl group of compound **9** to alumole as compared to that of **7** (23.9 kcal mol^−1^).

**Figure 2:**
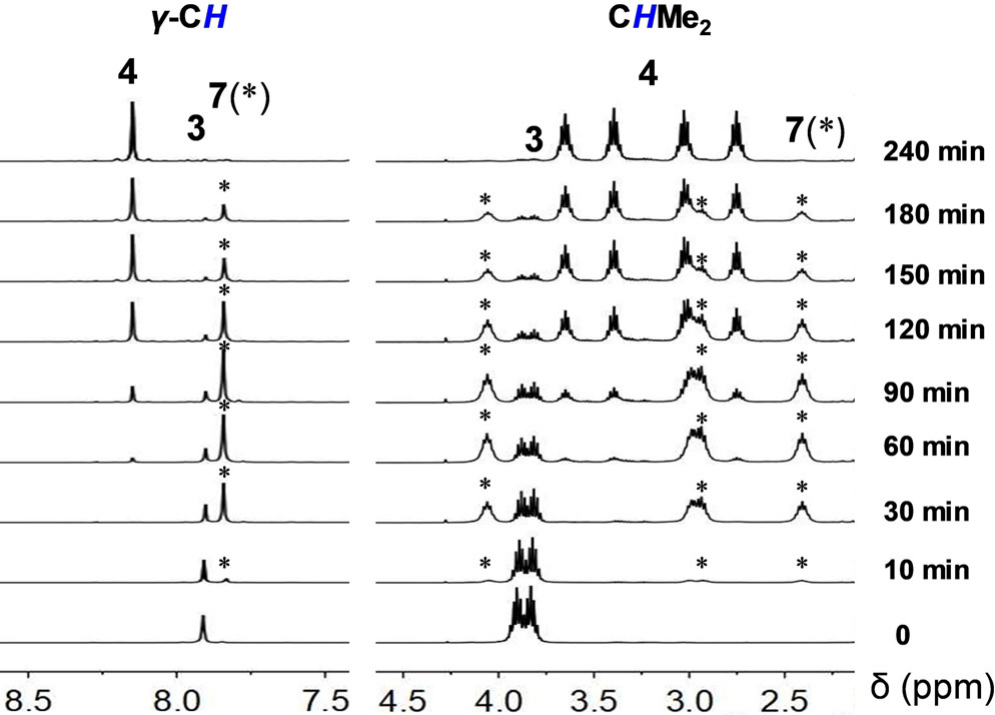
The ^1^H NMR spectral‐monitored progress on reaction of **3** and B(C_6_F_5_)_3_ to give **7** and **4** in C_6_D_6_ at 65 °C. The regions between *δ* 8.5–7.5 ppm for the *H*C=N of L^2^ skeleton and *δ* 4.5–2.0 ppm for the C*H*Me_2_ of the Ar substituent are shown.

**Scheme 3:**
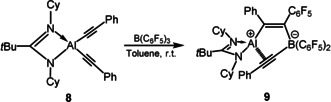
Reactions of aluminum dialkynyl compounds supported by amidinato ligands with B(C_6_F_5_)_3_ to form compound **9**.

Compounds **7** and **9** both exhibit a structure (Figure 3 for **7** and Figure S3‐3 for **9**) that is much different from those of **2**, **4** and **6**. The C(1)‐C(2) bonds (1.351(3) in **7** and 1.354(5) Å in **9**) have double bond character whereas the C(3)‐C(4) bonds (1.208(3) in **7** and 1.217(5) Å in **9**) exhibit triple bond characteristics. The Al(1)‐C(1) (1.972(2) in **7** and 1.939(4) Å in **9**) bond lengths are comparable to those found within the AlC_4_‐rings of **2**, **4** and **6** (1.972(2)–2.001(2) Å), respectively. However the separations of Al(1)‐C(3) (2.248(2) in **7** and 2.194(4) Å in **9**) and Al(1)‐C(4) (2.531(1) in **7** and 2.352(4) Å in **9**) are much longer than that of the common Al−C σ‐bond. This is indicative of a side‐on coordination of C(3)‐C(4) π‐bond to the aluminum center. The ^13^C NMR spectral data warrant the olefinic carbon resonances for C(1) and C(2) (for **7**, *δ* 129.6 (Al*C*=) and 158.1 ppm (=*C*B); for **9**, see the Supporting Information), as well as the alkynyl carbon resonances for C(3) and C(4) (for **7**, *δ* 118.3 (Ph*C*≡) and 123.9 ppm (≡*C*B); for **9**, see the Supporting Information). Note that the B atom adopts a tetrahedral coordination geometry, in agreement with the ^11^B NMR data (*δ*−20.7 in **7** and −20.2 ppm in **9**). Then the B center holds a negative charge and correspondingly the Al atom a positive charge; either **7** or **9** is indeed a zwitterionic compound. To our knowledge, compounds **7** and **9** may represent the first structural example exhibiting a definite π‐bond complexation between the cationic Al and the C≡C bond, although such interaction was only found at neutral and dimeric alkynyl aluminum compounds.^12^

**Figure 3:**
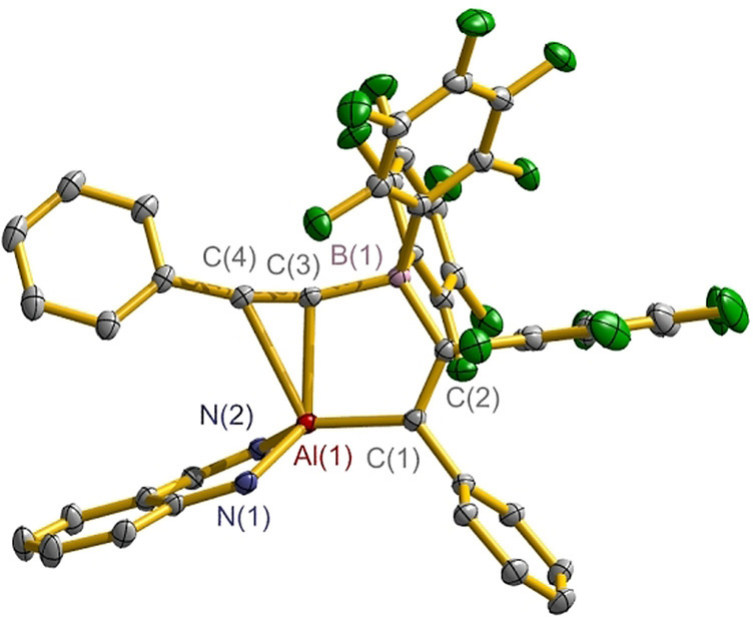
X‐ray crystal structure of **7** with thermal ellipsoids set at 50 % probability. H atoms and 2,6‐*i*Pr_2_C_6_H_3_ groups at N atoms are omitted for clarity. Selected bond lengths [Å] and angles [°]: Al(1)–N(1) 1.858(2), Al(1)–N(2) 1.929(2), Al(1)–C(1) 1.972(2), Al(1)–C(3) 2.248(2), Al(1)–C(4) 2.531(1), C(1)–C(2) 1.351(3), C(2)–B(1) 1.624(3), B(1)–C(3) 1.629(3), C(3)–C(4) 1.208(3); N(1)‐Al(1)‐N(2) 96.49(9), C(3)‐Al(1)‐C(4) 30.8(2).

To further understand the reaction mechanism for the alumole synthesis, DFT calculations at M06‐2X level were performed on reaction of **3** and B(C_6_F_5_)_3_ to **4** as the representative example (Figure 4). The reaction usually started with a commonly known interaction between the C≡C π‐electrons and the B center. The step experiences a moderate Gibbs energy barrier of 8.5 kcal mol^−1^ (for **TS1**), leading to the formation of a zwitteronic aluminum‐cation borate **Int1**. The subsequent 1,2‐migration of one C_6_F_5_ group and meanwhile a Al‐C_alkynyl_ bonding, overcoming a Gibbs energy barrier of 10.1 kcal mol^−1^ (for **TS2**), affords intermediate **Int2** that was energetically favored in fact. However, intermediate **7**, formed as a consequence by the second alkynyl group migration from the Al to B center, appeared to be more stable than the **int2** by 10.4 kcal mol^−1^ (between these two species exists **TS3** with a very small energy barrier of 1.5 kcal mol^−1^), and therefore it was possible to isolate **7** rather than the **int2**. Finally, climbing the **TS4** resulted in **4** as the most stable compound in the whole reaction process. The group exchange reaction has been known for the aluminum alkyl and B(C_6_F_5_)_3_.^13^ For the aluminum alkynyl, the alkynyl migration to the electron deficient B center is instead. It is worth mentioning that similar synthetic routes to the borole, silole, phosphole, and stannole compounds have also been reported using related dialkynyls with B(C_6_F_5_)_3_ by Erker et al.^14^ and previously with trialkylboron by Wrackmeyer et al.^15^ A phosphirenium borate and a vinylacetenylphosphine, which are, respectively similar to **Int1** and **Int2** in structure, were isolated and characterized as intermediates in the synthesis of phospholes.14c And also several tin or lead analogues of **7** (or **9**) with such side‐on coordination characteristics were reported by Wrackmeyer and co‐workers.^16^ The approach to the alumoles opens a door to the aluminum element, fulfilling a widely effective route for preparing main group III to VI element combinations.

**Figure 4:**
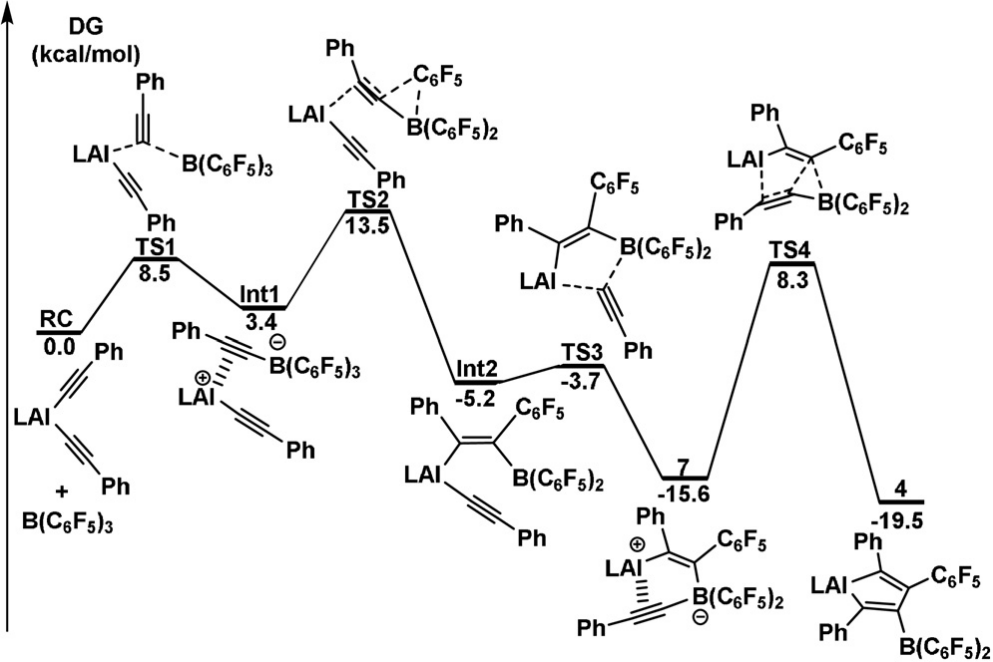
DFT‐calculated reaction mechanism of **3** and B(C_6_F_5_)_3_ to form **4** via **7** as a stable intermediate (L=*o*‐C_6_H_4_(CH=NAr)NAr, Ar=2,6‐*i*Pr_2_C_6_H_3_).

Alumoles **2** and **4** in benzene show similar absorption mode with peaks at 337 and 365 nm for **2**, and 337 nm and 366 nm for **4**, respectively (Figure S4‐1). However, their fluorescence emissions in benzene are very weak or almost negligible. Interestingly, they exhibit brightly yellow emissions (*λ*_em(maximum)_ at 565 nm for **2** and 566 nm for **4**, Figure 5) in the solid state. The luminescence lifetime for **2** was obtained at 13.7 ns (quantum yield *Φ*≈9.3 %) and for **4** at 4.3 ns (quantum yield *Φ*≈8.6 %) (Figures S4‐4 and S4‐5). This is in contrast to the conventional organic fluorophores showing good fluorescence in (dilute) solution but weak or even none in solid state because of a self‐quenching. Therefore, the fluorescence of both **2** and **4** may be of the typical aggregation‐induced emission (AIE) character more recently reported.^17^ Similar AIE property was also observed for the main group element‐containing heteroles.^18^ This AIE is often illustrated in terms of the restricted intramolecular rotation (RIR) mechanism.^19^ In **2** and **4**, there are arranged around the central AlC_4_ plane six rings (two Ph, three C_6_F_5_, and one AlN_2_C_3_). For that reason the RIR is especially remarkable in the solid state. The DFT calculations confirmed the charge density difference between the first lowest singlet excited state and the ground state (Figure S5‐2), and suggested an electron transfer from the π orbitals over the AlC_4_‐ring to the p orbital of the B center. The alumole **6** exhibits the absorption peaks at 394 and 445 nm, but shows no fluorescence emission both in the solid and the solution state (Figures S4‐2 and S4‐3). This is probably due to the deviation of the central AlC_4_ ring from the plane with the consequence of a lowered RIR.

**Figure 5:**
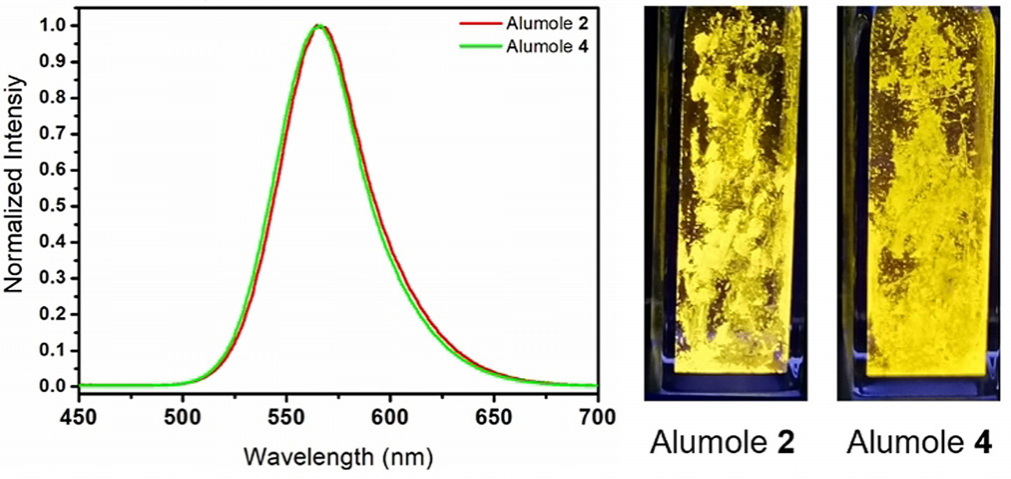
Normalized fluorescent emission spectra and pictures of fluorescent emissions of alumoles **2** and **4** in the solid state (365 nm excitation).

In summary, we have found a facile route to synthesize the alumoles. Alumoles **2** and **4** both exhibit aggregation‐induced emission (AIE) because of restricted intramolecular rotations (RIR); alumole **6** does not have this property. The intramolecular aluminum borate zwitterions **7** and **9** each with Al⋅⋅⋅η^2^‐C≡C side‐on coordination structure are suggested as the intermediates. These results show a distinctive route so far not known in the organoaluminum chemistry for the preparation of the aluminum containing heterocycles.

## Conflict of interest

The authors declare no conflict of interest.

## Supporting information

As a service to our authors and readers, this journal provides supporting information supplied by the authors. Such materials are peer reviewed and may be re‐organized for online delivery, but are not copy‐edited or typeset. Technical support issues arising from supporting information (other than missing files) should be addressed to the authors.

Supplementary

## References

[1a] C. Zhao , P. Li , Z. Xi , Chem. Eur. J. 2002, 8, 4292–4298; 12298021 10.1002/1521-3765(20020916)8:18<4292::AID-CHEM4292>3.0.CO;2-G

[1b] J. J. Eisch , W. C. Kaska , J. Am. Chem. Soc. 1962, 84, 1501–1502;

[1c] J. J. Eisch , W. C. Kaska , J. Am. Chem. Soc. 1966, 88, 2976–2983;

[1d] H. Hoberg , W. Richter , J. Organomet. Chem. 1980, 195, 347–353.

[2a] H. Hoberg , R. Krause-Göing , J. Organomet. Chem. 1977, 127, C29–C31;

[2b] T. Agou , T. Wasano , P. Jin , S. Nagase , N. Tokitoh , Angew. Chem. Int. Ed. 2013, 52, 10031–10034; 10.1002/anie.201304143 23929506

[2c] T. Wasano , T. Agou , T. Sasamori , N. Tokitoh , Chem. Commun. 2014, 50, 8148–8150; 10.1039/c4cc03470h 24874063

[2d] Y. Zhang , J. Wei , W.-X. Zhang , Z. Xi , Inorg. Chem. 2015, 54, 10695–10700. 26501357 10.1021/acs.inorgchem.5b01551

[3a] Z. Xi , P. Li , Angew. Chem. Int. Ed. 2000, 39, 2950–2952; 11028021

[3b] U. M. Dzhemilev , A. G. Ibragimov , L. O. Khafizova , L. R. Yakupova , L. M. Khalilov , Russ. J. Org. Chem. 2005, 41, 667–672;

[3c] E. Negishi , D. Y. Kondakov , D. Choueiry , K. Kasai , T. Takahshi , J. Am. Chem. Soc. 1996, 118, 9577–9588.

[4a] C. Cui , S. Köpke , R. Herbst-Irmer , H. W. Roesky , M. Noltemeyer , H.-G. Schmidt , B. Wrackmeyer , J. Am. Chem. Soc. 2001, 123, 9091–9098; 11552816 10.1021/ja003185i

[4b] H. Zhu , R. B. Oswald , H. Fan , H. W. Roesky , Q. Ma , Z. Yang , H.-G. Schmidt , M. Noltemeyer , K. Starke , N. S. Hosmane , J. Am. Chem. Soc. 2006, 128, 5100–5108. 16608344 10.1021/ja057731p

[5] H. Zhu , J. Chai , V. Chandrasekhar , H. W. Roesky , J. Magull , D. Vidovic , H.-G. Schmidt , M. Noltemeyer , P. P. Power , W. A. Merrill , J. Am. Chem. Soc. 2004, 126, 9472–9473. 15291514 10.1021/ja0475712

[6] X. Li , C. Ni , H. Song , C. Cui , Chem. Commun. 2006, 1763–1765. 10.1039/b601056c 16609797

[7] X. Li , L. Duan , H. Song , C. Ni , C. Cui , Organometallics 2006, 25, 5665–5667.

[8] H. Zhu , J. Chai , Q. Ma , V. Jancik , H. W. Roesky , H. Fan , R. Herbst-Irmer , J. Am. Chem. Soc. 2004, 126, 10194–10195. 15315402 10.1021/ja049462t

[9] H. Zhu , J. Chai , H. Fan , H. W. Roesky , C. He , V. Jancik , H.-G. Schmidt , M. Noltemeyer , W. A. Merrill , P. P. Power , Angew. Chem. Int. Ed. 2005, 44, 5090–5093; 10.1002/anie.200500899 16015667

[10] W. Zheng , H. W. Roesky , J. Chem. Soc. Dalton Trans. 2002, 2787–2796.

[11] A. G. Massey , A. J. Park , J. Organomet. Chem. 1964, 2, 245–250.

[12a] W. Uhl , F. Breher , S. Haddadpour , R. Koch , M. Matar , Z. Anorg. Allg. Chem. 2004, 630, 1839–1845;

[12b] W. Uhl , E. Er , O. Hübner , H.-J. Himmel , Z. Anorg. Allg. Chem. 2008, 634, 2133–2139.

[13a] G. S. Hair , A. H. Cowley , R. A. Jones , B. G. McBurnett , A. Voigt , J. Am. Chem. Soc. 1999, 121, 4922–4923;

[13b] J. Chen , E. Y. X. Chen , Dalton Trans. 2016, 45, 6105–6110. 26567780 10.1039/c5dt03895b

[14a] G. Kehr , G. Erker , Chem. Commun. 2012, 48, 1839–1850; 10.1039/c1cc15628d 22116402

[14b] J. Ugolotti , G. Kehr , R. Fröhlich , G. Erker , Chem. Commun. 2010, 46, 3016–3018; 10.1039/b927221f 20386853

[14c] J. Möbus , Q. Bonnin , K. Ueda , R. Fröhlich , K. Itami , G. Kehr , G. Erker , Angew. Chem. Int. Ed. 2012, 51, 1954–1957; 10.1002/anie.201107398 22241588

[14d] J. Möbus , A. Galstyan , A. Feldmann , C. G. Daniliuc , R. Fröhlich , C. A. Strassert , G. Kehr , G. Erker , Chem. Eur. J. 2014, 20, 11883–11893; 25123406 10.1002/chem.201403102

[14e] C. Eller , G. Kehr , C. G. Daniliuc , D. W. Stephan , G. Erker , Chem. Commun. 2015, 51, 7226–7229; 10.1039/c5cc01806d 25813555

[14f] F. Ge , G. Kehr , C. G. Daniliuc , G. Erker , J. Am. Chem. Soc. 2014, 136, 68–71; 24354317 10.1021/ja4110396

[14g] F. Ge , G. Kehr , C. G. Daniliuc , G. Erker , Organometallics 2015, 34, 229–235.

[15a] B. Wrackmeyer , S. Bayer , W. Milius , E. V. Elena , J. Organomet. Chem. 2018, 865, 80–88;

[15b] E. Khan , B. Wrackmeyer , R. Kempe , G. Glatz , Appl. Organomet. Chem. 2015, 29, 384–391;

[15c] B. Wrackmeyer , B. Bernd , H. Moazzam , S. Ali , L. Oleg , Y. N. Yuri , J. Organomet. Chem. 2002, 657, 146–154;

[15d] B. Wrackmeyer , G. Kehr , J. Suess , Chem. Ber. 1993, 126, 2221–2226;

[15e] B. Wrackmeyer , G. Kehr , D. Wettinger , Inorg. Chim. Acta 1994, 220, 161–173.

[16a] B. Wrackmeyer , P. Thoma , S. Marx , G. Glatz , R. Kempe , Z. Anorg. Allg. Chem. 2013, 639, 1205–1213;

[16b] B. Wrackmeyer , S. Kundler , R. Boese , Chem. Ber. 1993, 126, 1361–1370;

[16c] B. Wrackmeyer , P. Thoma , S. Marx , T. Bauer , R. Kempe , Eur. J. Inorg. Chem. 2014, 2103–2112;

[16d] B. Wrackmeyer , S. Kundler , W. Milius , R. Boese , Chem. Ber. 1994, 127, 333–342;

[16e] B. Wrackmeyer , K. Horchler , R. Boese , Angew. Chem. Int. Ed. Engl. 1989, 28, 1500–1502;

[17a] J. Luo , Z. Xie , J. Y. Lam , C. Lin , H. Chen , C. Qiu , H. S. Kwok , X. Zhan , Y. Liu , D. Zhu , B. Z. Tang , Chem. Commun. 2001, 1740–1741; 10.1039/b105159h 12240292

[17b] Y. Hong , J. Y. Lam , B. Z. Tang , Chem. Soc. Rev. 2011, 40, 5361–5388; 21799992 10.1039/c1cs15113d

[17c] Z. Zhao , B. He , B. Z. Tang , Chem. Sci. 2015, 6, 5347–5365; 28717442 10.1039/c5sc01946j PMC5502404

[17d] M. Gao , B. Z. Tang , Coord. Chem. Rev. 2020, 402, 213076–221417.

[18a] T. Baumgartner , R. Réau , Chem. Rev. 2006, 106, 4681–4727; 17091932 10.1021/cr040179m

[18b] G. He , W. T. Delgado , D. J. Schatz , C. Merten , A. Mohammadpour , L. Mayr , M. J. Ferguson , R. McDonald , A. Brown , K. Shankar , E. Rivard , Angew. Chem. Int. Ed. 2014, 53, 4587–4591; 10.1002/anie.201307373 24668889

[18c] S. M. Parke , E. Hupf , G. K. Matharu , I. de Aguiar , L. Xu , H. Yu , M. P. Boone , G. L. C. de Souza , R. McDonald , M. J. Ferguson , G. He , A. Brown , E. Rivard , Angew. Chem. Int. Ed. 2018, 57, 14841–14846; 10.1002/anie.201809357 30239084

[18d] H. Imoto , A. Urushizaki , I. Kawashima , K. Naka , Chem. Eur. J. 2018, 24, 8797–8803. 29719074 10.1002/chem.201801589

[19a] Y. Hong , J. W. Y. Lam , B. Z. Tang , Chem. Commun. 2009, 4332–4353; 10.1039/b904665h 19597589

[19b] J. Chen , B. Z. Tang , Aggregation-Induced Emission: Fundamentals (Eds.: A. Qin, B. Z. Tang), Wiley, Hoboken, 2014, pp. 307–322.

