# Supplementary material for: An Unusual and Facile Synthetic Route to Alumoles
Source: Angew Chem Int Ed Engl. 2020 Apr 23;59(25):10027–31. doi: 10.1002/anie.202000899 (PMC7318123; doi:10.1002/anie.202000899)
Supplement: Supplementary file 1 — Supplementary [file ANIE-59-10027-s001.pdf]

Angewandte Chemie
Eine Zeitschrift der Gesellschaft Deutscher Chemiker

Supporting Information

An Unusual and Facile Synthetic Route to Alumoles

Jiancheng Li, Peng Wu, Wenjun Jiang, Bin Li, Binju Wang, Hongping Zhu,* and Herbert W. Roesky**

anie_202000899_sm_miscellaneous_information.pdf

Supporting Information

Content:

I. Experimental section

II. NMR spectra-detected reactions

III. X-Ray crystallographic details

IV. The UV-vis absorption/emission spectra and luminescence lifetime measurements

V. Theoretical calculation study

VI. Collected ^{1}H, ^{13}C, ^{19}F and ^{11}B NMR spectra

VII. References

I. Experimental section

Materials and Methods All manipulations were carried out under dry argon or nitrogen atmosphere by using Schlenk line and glovebox techniques. Organic solvents as toluene, *n*-hexane and diethyl ether were dried by refluxing with sodium/potassium benzophenone under N_2 prior to use. NMR (^{1}H, ^{11}B, ^{13}C and ^{19}F) spectra were recorded on Bruker Avance II 400 spectrometer. Melting point of compound was measured in a sealed glass tube using the Büchi-540 instrument. Elemental analysis was performed on a Thermo Quest Italia SPA EA 1110 instrument. Commercial reagents were purchased from Energy Chemical or J&K Chemical Co. and used as received. Compounds $L^1Al(C{\equiv}CPh)_2$ (L^1 = $CH(CMeNAr)_2$, Ar = 2,6-$i$$Pr_2C_6H_3$) (**1**),[S1] L^2AlCl_2 (L^2 = *o*-$C_6H_4(CH{=}NAr)(NAr)$, Ar = 2,6-$i$$Pr_2C_6H_3$),[S2] L^3AlCl_2 (L^3 = *t*BuC(NCy)$_2$, Cy = *cyclo*-C_6H_{11}),[S3] and $B(C_6F_5)_3$[S4] were prepared according to literature procedures.

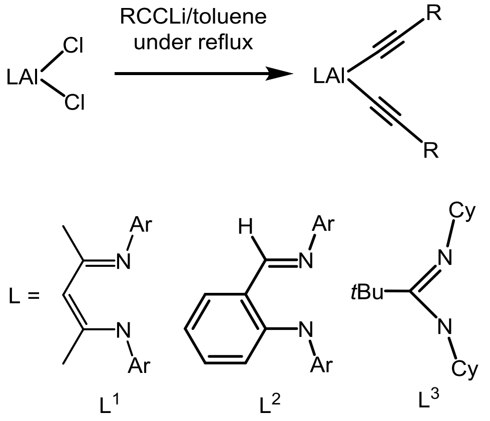

Scheme S1. Preparation of the ligand stabilized aluminum dialkynyls.

$L^1Al(C{\equiv}C$*t*Bu)$_2$ (L^1 = $CH(CMeNAr)_2$, Ar = 2,6-$i$$Pr_2C_6H_3$, **1a**) A suspension of *t*BuCCLi, freshly prepared from the reaction of *t*BuCCH (0.82 g, 10 mmol) and *n*BuLi (4.2 mL 2.4 M *n*-hexane solution, 10 mmol) in toluene (30 mL) from –20 ℃ to room temperature in the period of 10 h, was added dropwise to a solution of L^1AlCl_2 (2.5 g, 5 mmol) in toluene (30 mL) at –20 ℃ under stirring. After addition, the mixture was allowed to heat at reflux for 24 h. Then the reaction mixture was cooled to room temperature. The insoluble LiCl was removed by filtration, and the filtrate was evaporated to dryness under reduced pressure, yielding an off-white solid of **1a** that was washed with cooled *n*-hexane (10 mL at –20 ℃). Yield: 2.7 g, 90%. Mp: 167 ℃. ^{1}H NMR (400 MHz, C_6D_6, 298 K, ppm): δ = 1.02 (s, 9 H, C*Me*$_3$), 1.23 (d, $^{3}J_{HH}$ = 6.7 Hz, 12 H, CH*Me*$_2$), 1.51 (d, $^{3}J_{HH}$ = 6.7 Hz, 12 H, CH*Me*$_2$), 1.51 (s, 6 H, *β-Me*), 3.69 (sept, $^{3}J_{HH}$ = 6.7 Hz, 4 H, C*H*Me$_2$), 4.92 (s, 1 H, *γ-H*), 7.13 (m, 6 H, C_6H_3). ^{13}C{^{1}H} NMR (100 MHz, C_6D_6, 298 K, ppm): δ = 23.3, 24.1, 26.2 (CH*Me*$_2$) 27.8 (*β-Me*), 28.1 (C*Me*$_3$), 31.1 (*C*Me$_3$), 93.9 (Al*C*≡), 98.4 (γ-*C*), 116.2 (*t*Bu*C*≡), 124.0, 126.8, 140.5, 144.4 (*C*$_6$H$_3$), 170.15 (*C*=N). Anal. calcd (%) for $C_{41}H_{59}AlN_2$ (M_r = 606.92): C, 81.14; H, 9.80; N, 4.62. Found: C, 81.42; H, 9.76; N, 4.39.

$L^1Al(C{\equiv}CSiMe_3)_2$ (L^1 = $CH(CMeNAr)_2$, Ar = 2,6-$i$$Pr_2C_6H_3$, **1b**) A suspension of $Me_3SiCCLi$, freshly prepared from the reaction of Me_3SiCCH (0.98 g, 10 mmol) and *n*BuLi (4.2 mL 2.4 M

n-hexane solution, 10 mmol) in toluene (30 mL) from –20 ℃ to room temperature in the period of 10 h, was added drop by drop to a solution of L^1AlCl_2 (2.5 g, 5 mmol) in toluene (30 mL) at –20 ℃ under stirring. After addition, the mixture was allowed to heat at reflux for 12 h. Then the reaction mixture was cooled to room temperature, from which the insoluble LiCl was removed by filtration. The filtrate was evaporated to dryness under reduced pressure, producing an off-white solid of **1b** that was washed with cooled *n*-hexane (10 mL at –20 ℃). Yield: 2.6 g, 82%. Mp: 143 ℃. ^1H NMR (400 MHz, C_6D_6, 298 K, ppm): δ = –0.01 (s, 9 H, Si*Me*$_3$), 1.21 (d, $^3J_{HH}$ = 6.8 Hz, 12 H, CH*Me*$_2$), 1.48 (s, 6 H, *β-Me*), 1.51 (d, $^3J_{HH}$ = 6.8 Hz, 12 H, CH*Me*$_2$), 3.64 (sept, $^3J_{HH}$ = 6.8 Hz, 4 H, C*H*Me$_2$), 4.82 (s, 1 H, γ-*H*), 7.14 (m, 6 H, C_6H_3). $^{13}C\{^1H\}$ NMR (100 MHz, C_6D_6, 298 K, ppm): δ = 0.0 (Si*Me*$_3$), 23.2, 24.2, 26.2 (*C*H*Me*$_2$), 28.2 (*β-Me*), 93.6 (Al*C*≡), 98.4 (γ-*C*), 114.9 (Si*C*≡), 124.2, 127.1, 139.7, 144.4, (*C*$_6$H$_3$), 170.5 (*C*=N). ^{29}Si NMR (79 MHz, C_6D_6, 298 K, ppm): δ = –22.3 (*Si*Me$_3$). Anal. calcd (%) for $C_{39}H_{59}AlN_2Si_2$ (M_r = 639.07): C, 73.30; H, 9.31; N, 4.38. Found: C, 73.02; H, 9.54; N, 4.76.

$L^1Al\{C(Ph)=C(C_6F_5)C[B(C_6F_5)_2]=C(Ph)\}$ (L^1 = CH(MeCNAr)$_2$, Ar = 2,6-*i*Pr$_2$C$_6$H$_3$, **2**) At room temperature, toluene (20 mL) was added to a mixture of **1** (128 mg, 0.2 mmol) and $B(C_6F_5)_3$ (100 mg, 0.2 mmol). This solution was allowed to heat at 65 ℃ for 10 h, during which a color change from colorless to orange-red was observed. After cooling to room temperature, the solution was concentrated to ca. 5 mL. The addition of *n*-hexane (10 mL) led to precipitation of **2** as an orange solid that was collected by filtration and washed with *n*-hexane. Yield: 180.7 mg, 78%. Mp: 243 ℃ (dec.). ^1H NMR (400 MHz, C_6D_6, 298 K, ppm): δ = 0.77 (d, $^3J_{HH}$ = 6.5 Hz, 6 H, CH*Me*$_2$), 0.89 (d, $^3J_{HH}$ = 6.5 Hz, 6 H, CH*Me*$_2$), 0.97 (d, $^3J_{HH}$ = 6.5 Hz, 6 H, CH*Me*$_2$), 1.47 (s, 6 H, *β-Me*), 1.54 (d, $^3J_{HH}$ = 6.5 Hz, 6 H, CH*Me*$_2$), 2.91 (sept, $^3J_{HH}$ = 6.5 Hz, 2 H, C*H*Me$_2$), 3.31 (sept, $^3J_{HH}$ = 6.5 Hz, 2 H, C*H*Me$_2$), 5.09 (s, 1 H, *γ-H*), 6.21 (m), 6.42 (m), 6.55 (m), 6.95–7.31 (m, 16 H) (C$_6$*H*$_3$ and C$_6$*H*$_5$). $^{13}C\{^1H\}$ NMR (100 MHz, C_6D_6, 298 K, ppm): δ = 23.9, 24.5, 24.8, 28.2, 29.0, 31.3 (*C*H*Me*$_2$), 38.2 (*β-Me*), 99.7 (*γ-C*), 123.8, 124.9, 125.8, 126.3, 126.5, 128.9, 131.7, 131.8, 140.5, 142.4, 142.8, 145.2 (*C*$_6$H$_3$, *C*$_6$H$_5$), 131.6, 157.3, 170.0, 170.4 (Al*C*$_4$), 172.5 (*C*=N), 115.3 (m), 118.8 (m, br), 131.5 (m, br), 135.5 (m, br), 137.9 (m, br), 141.0 (m, br), 145.3 (m, br), 147.8 (m, br), 149.3 (br) (*C*$_6$F$_5$ and B(*C*$_6$F$_5$)$_2$). ^{19}F NMR (376 MHz, 298, C_6D_6, ppm): δ = –127.1 (m, 4 F, *o*-F_{BC6F5}), –135.5 (m, 2 F, *o*-F_{CC6F5}), –148.9 (t, $^3J_{FF}$ = 21.4 Hz, 1 F, *p*-F_{CC6F5}), –157.2 (t, $^3J_{FF}$ = 21.0 Hz, 2 F, *p*-F_{BC6F5}), –162.3 (m, 2 F, *m*-F_{CC6F5}), –162.6 (m, 4 F, *m*-F_{BC6F5}). ^{11}B NMR (128 MHz, 298 K, C_6D_6, ppm): δ = 62.4. Anal. calcd (%) for $C_{63}H_{51}AlBF_{15}N_2$ (M_r = 1158.88): C, 65.30; H, 4.44; N, 2.42. Found: C, 64.95; H, 4.83; N, 2.68.

$L^2Al(C\equiv CPh)_2$ (L^2 = *o*-C$_6$H$_4$(CH=NAr)(NAr), Ar = 2,6-*i*Pr$_2$C$_6$H$_3$, **3**) A suspension of PhCCLi, freshly prepared from the reaction of PhCCH (1.022 g, 10 mmol) and *n*BuLi (4.17 mL 2.4 M *n*-hexane solution, 10 mmol) in toluene (30 mL) from –20 ℃ to room temperature in the period of 10 h, was added drop by drop to a solution of L^2AlCl_2 (2.7 g, 5 mmol) in toluene (30 mL) at –20 ℃ under stirring. After addition, the mixture was allowed to heat at 80 ℃ for 10 h. Then the reaction mixture was cooled to room temperature, from which the insoluble LiCl was removed by filtration. The filtrate was evaporated to dryness under reduced pressure, producing a light-yellow solid of **3** that was washed with cooled *n*-hexane (10 mL at –20 ℃). Yield: 2.8 g, 85%. Mp: 197 ℃. ^1H NMR (400 MHz, C_6D_6, 298 K, ppm): δ = 1.05 (d, $^3J_{HH}$ = 6.8 Hz, 6 H, CH*Me*$_2$), 1.14 (d, $^3J_{HH}$ = 6.8 Hz, 6 H, CH*Me*$_2$), 1.42 (d, $^3J_{HH}$ = 6.8 Hz, 6 H,

CH*Me*$_2$), 1.57 (d, $^3J_{HH}$ = 6.8 Hz, 6 H, CH*Me*$_2$), 3.83 (sept, $^3J_{HH}$ = 6.8 Hz, 4 H, C*H*Me$_2$), 3.90 (sept, $^3J_{HH}$ = 6.8 Hz, 4 H, C*H*Me$_2$), 6.23 (m), 6.45 (m), 6.72–6.90 (m), 7.13–7.31 (m) (20 H, C_6H_3, C_6H_4 and C_6H_5), 7.92 (s, 1 H, *H*C=N). ^{13}C{^{1}H} NMR (100 MHz, C_6D_6, 298 K, ppm): δ = 23.2, 24.8, 25.3, 25.5 (CH*Me*$_2$), 28.8, 29.1 (*C*HMe$_2$), 102.5 (Al*C*≡), 108.3 (Ph*C*≡), 115.2, 115.8, 119.1, 124.3, 124.7, 125.1, 127.2, 128.4, 132.0, 136.3, 136.7, 139.4, 142.5, 143.2, 146.6, 157.7 (*C*$_6$H$_3$, *C*$_6$H$_4$ and *C*$_6$H$_5$), 172.4 (*C*=N). Anal. calcd (%) for $C_{47}H_{49}AlN_2$ (M_r = 668.90): C, 84.39; H, 7.38; N, 4.19. Found: C, 84.58; H, 7.12; N, 4.32.

L^{2}Al{C(Ph)=C(C_6F_5)C[B(C_6F_5)$_2$]=C(Ph)} (L^2 = *o*-C_6H_4(CH=NAr)(NAr), Ar = 2,6-*i*Pr$_2$C$_6$H$_3$, **4**) At room temperature, toluene (20 mL) was added to a mixture of **3** (134 mg, 0.2 mmol) and B(C_6F_5)$_3$ (100 mg, 0.2 mmol). This solution was allowed to heat at 65 ℃ for 12 h. After cooling to room temperature, the solution was concentrated to ca. 5 mL. By storing the solution at 4 ℃ for 2 days, yellow crystals of **4** were obtained which were collected by filtration and washed with *n*-hexane. Yield：191 mg, 71%. Mp: 275 ℃ (dec.). ^{1}H NMR (400 MHz, C_6D_6, 298 K, ppm): δ = 0.47 (d, $^3J_{HH}$ = 6.6 Hz, 3 H, CH*Me*$_2$), 0.75 (d, $^3J_{HH}$ = 6.6 Hz, 3 H, CH*Me*$_2$), 0.75 (d, $^3J_{HH}$ = 6.6 Hz, 3 H, CH*Me*$_2$), 0.80 (d, $^3J_{HH}$ = 6.6 Hz, 3 H, CH*Me*$_2$), 1.02 (d, $^3J_{HH}$ = 6.6 Hz, 3 H, CH*Me*$_2$), 1.09 (d, $^3J_{HH}$ = 6.6 Hz, 3 H, CH*Me*$_2$), 1.43 (m, 3 H, CH*Me*$_2$), 1.58 (d, $^3J_{HH}$ = 6.6 Hz, 3 H, CH*Me*$_2$), 2.75 (sept, $^3J_{HH}$ = 6.6 Hz, 1 H, C*H*Me$_2$), 3.03 (sept, $^3J_{HH}$ = 6.6 Hz, 1 H, C*H*Me$_2$), 3.39 (sept, $^3J_{HH}$ = 6.8 Hz, 1 H, C*H*Me$_2$), 3.66 (sept, $^3J_{HH}$ = 6.7 Hz, 1 H, C*H*Me$_2$), 6.20−7.40 (m, 20 H) (C_6H_3, C_6H_4 and C_6H_5), 8.11 (s, 1 H, C*H*=N). ^{13}C{^{1}H} NMR (100 MHz, C_6D_6, 298 K, ppm): δ = 20.6, 21.6 (d, *J* = 11.0 Hz), 23.2 (d, *J* = 4.4 Hz), 23.8, 24.9, 25.2, 25.8, 25.9 (CH*Me*$_2$), 28.5, 28.6, 28.8, 29.4 (*C*HMe$_2$), 116.3, 120.8, 123.5, 124.6, 124.7, 125.0, 126.1, 126.3, 126.7, 128.5, 135.4, 136.1, 141.0, 141.4, 141.8, 142.8, 144.5, 147.4, 147.5, 158.3 (*C*$_6$H$_3$, *C*$_6$H$_4$ and *C*$_6$H$_5$), 174.2 (H*C*=N), 135.1, 156.2, 170.7, 171.2 (Al*C*$_4$), 115.1 (m), 119.1 (m, br), 131.5 (m, br), 138.0 (m, br), 141.2 (m, br), 145.5 (m, br), 148.0 (m, br) (*C*$_6$F$_5$ and B(*C*$_6$F$_5$)$_2$). ^{19}F NMR (376 MHz, 298, C_6D_6, ppm): δ = –127.6 (m, 4 F, *o*-F_{BC6F5}), (–136.6, –137.1) (m, 2 F, *o*-F_{CC6F5}), –148.6 (t, $^3J_{FF}$ = 21.4 Hz, 1 F, *p*-F_{CC6F5}), –157.0 (t, $^3J_{FF}$ = 21.0 Hz, 2 F, *p*-F_{BC6F5}), (–161.6, –162.9) (m, 2 F, *m*-F_{CC6F5}), –162.4 (m, 4 F, *m*-F_{BC6F5}). ^{11}B NMR (128 MHz, 298 K, C_6D_6, ppm): δ = 64.1. Anal. calcd (%) for $C_{65}H_{49}AlBF_{15}N_2$ (M_r =1180.89): C, 66.11; H, 4.18; N, 2.37. Found: C, 66.51; H, 4.03; N, 2.34.

L^{2}Al(C≡C(2-thienyl))$_2$ (L^2 = *o*-C_6H_4(CH=NAr)(NAr), Ar = 2,6-*i*Pr$_2$C$_6$H$_3$, **5**) A suspension of (2-C_4H_3S)CCLi, freshly prepared from the reaction of (2-C_4H_3S)CCH (0.43 g, 4 mmol) and *n*BuLi (1.7 mL 2.4 M *n*-hexane solution, 4 mmol) in toluene (30 mL) from –20 ℃ to room temperature in the period of 10 h, was added drop by drop to a solution of L^{2}AlCl$_2$ (1.1 g, 2 mmol) in toluene (30 mL) at room temperature under stirring. After addition, the mixture was allowed to heat at 100 ℃ for 10 h. Then the reaction mixture was cooled to room temperature, from which the insoluble LiCl was removed by filtration. The filtrate was evaporated to dryness under reduced pressure, producing a light-yellow solid of **5** that was washed with cooled *n*-hexane (10 mL at –20 ℃). Yield: 2.3 g, 86%. Mp: 204 ℃. ^{1}H NMR (400 MHz, C_6D_6, 298 K, ppm): δ = 1.04 (d, $^3J_{HH}$ = 6.8 Hz, 6 H, CH*Me*$_2$), 1.11 (d, $^3J_{HH}$ = 6.8 Hz, 6 H, CH*Me*$_2$), 1.47 (d, $^3J_{HH}$ = 6.8 Hz, 6 H, CH*Me*$_2$), 1.59 (d, $^3J_{HH}$ = 6.8 Hz, 6 H, CH*Me*$_2$), 3.77 (sept, $^3J_{HH}$ = 6.8 Hz, 2 H, C*H*Me$_2$), 3.83 (sept, $^3J_{HH}$ = 6.8 Hz, 2 H, C*H*Me$_2$), 6.38 (dd, $^3J_{HH}$ = 5.1, $^3J_{HH}$ = 3.6 Hz, 2 H, 2-C$_4$*H*$_3$S), 6.48 (dd, $^3J_{HH}$ = 5.2, $^4J_{HH}$ = 1.1 Hz, 2 H, 2-C$_4$*H*$_3$S), 6.82 (dd, $^3J_{HH}$ = 3.6, $^4J_{HH}$ = 1.1 Hz, 2 H, 2-C$_4$*H*$_3$S), 6.24 (m), 6.42 (m), 6.74 (m) (4 H, C$_6$*H*$_4$) 7.10–

7.30 (m) (6 H, C_6H_3), 7.91 (s, 1 H, *H*C=N). $^{13}C\{^1H\}$ NMR (100 MHz, C_6D_6, 298 K, ppm): δ = 23.6, 25.3, 25.7, 25.9 (CH*Me*$_2$), 29.2, 29.5 (*C*HMe$_2$), 101.0 ((2-C_4H_3S)*C*≡), 107.6 (Al*C*≡), 115.5, 116.3, 119.4, 124.8, 152.2, 125.7, 126.0, 126.6, 128.8, 132.1, 136.8, 137.2, 139.4, 142.7, 143.6, 147.0, 158.0 (*C*$_6$H$_3$, *C*$_6$H$_4$ and 2-*C*$_4$H$_3$), 172.9 (*C*=N). Anal. calcd (%) for $C_{47}H_{49}AlN_2$ (M_r = 668.90): C, 78.85; H, 6.66; N, 4.11; S, 9.42. Found: C, 78.42; H, 6.89; N, 4.18; S, 9.28.

L^{2}Al{C(2-thienyl)=C(C$_6$F$_5$)C[B(C$_6$F$_5$)$_2$]=C(2-thienyl)} (L^2 = *o*-C$_6$H$_4$(CH=NAr)NAr, Ar = 2,6-*i*Pr$_2$C$_6$H$_3$, 6) At room temperature, toluene (20 mL) was added to a mixture of **5** (136 mg, 0.2 mmol) and $B(C_6F_5)_3$ (100 mg, 0.2 mmol). This solution was allowed to heat at 65 ℃ and stirred for 10 h. After cooling to room temperature, the solution was concentrated to ca. 3 mL. By storing the solution in a freezer at 4 ℃ for 2 days, red crystals **6** were obtained which were collected by filtration and washed with n-hexane. Yield：205 mg, 86%. Mp: 283 ℃ (dec.). ^1H NMR (400 MHz, C_6D_6, 298 K, ppm): δ = 0.77 (d, $^3J_{HH}$ = 6.6 Hz, 3 H, CH*Me*$_2$), 0.81 (d, $^3J_{HH}$ = 6.6 Hz, 3 H, CH*Me*$_2$), 0.83 (d, $^3J_{HH}$ = 6.6 Hz, 3 H, CH*Me*$_2$), 0.93 (d, $^3J_{HH}$ = 6.7 Hz, 3 H, CH*Me*$_2$), 0.94 (d, $^3J_{HH}$ = 6.6 Hz, 3 H, CH*Me*$_2$), 1.17 (d, $^3J_{HH}$ = 6.6 Hz, 3 H, CH*Me*$_2$), 1.20 (m, 3 H, CH*Me*$_2$), 1.47 (d, $^3J_{HH}$ = 6.6 Hz, 3 H, CH*Me*$_2$), 3.04 (sept, $^3J_{HH}$ = 6.6 Hz, 1 H, C*H*Me$_2$), 3.14 (sept, $^3J_{HH}$ = 6.7 Hz, 1 H, C*H*Me$_2$), 3.26 (sept, $^3J_{HH}$ = 6.8 Hz, 1 H, C*H*Me$_2$), 3.53 (sept, $^3J_{HH}$ = 6.7 Hz, 1 H, C*H*Me$_2$), 5.90 (dd, $^3J_{HH}$ = 3.4 Hz, $^4J_{HH}$ = 1.0 Hz, 1 H, C$_4$*H*$_3$S), 5.97 (dd, $^3J_{HH}$ = 5.1 Hz, $^4J_{HH}$ = 1.0 Hz, 1 H, C$_4$*H*$_3$S), 6.07 (dd, $^3J_{HH}$ = 5.1 Hz, $^3J_{HH}$ =3.4 Hz, 1 H, C$_4$*H*$_3$S), 6.30−7.40 (m, 13 H) (C$_6$*H*$_3$, C$_6$*H*$_4$ and C$_4$*H*$_3$S), 8.10 (s, 1 H, C*H*=N). $^{13}C\{^1H\}$ NMR (100 MHz, C_6D_6, 298 K, ppm): δ = 20.7, 21.9 (d, *J* = 11.1 Hz), 23.0 (d, *J* = 6.3 Hz), 23.9, 25.1, 25.2, 25.6, 25.7 (CH*Me*$_2$), 28.4, 28.6, 29.4, 29.7 (*C*HMe$_2$), 116.1, 117.1, 121.1, 122.4, 123.3, 124.2, 124.6, 124.8, 150.0, 125.8, 126.7, 128.5, 130.1, 131.0, 135.7, 136.3, 141.0, 141.1, 142.9, 143.2, 144.2, 145.4, 147.3, 151.6, 158.3 (*C*$_6$H$_3$, *C*$_6$H$_4$ and *C*$_4$H$_3$S) 174.1 (H*C*=N) 132.5, 154.2, 173.7, 174.6 (Al*C*$_4$), 114.2 (m), 117.6 (m, br), 135.5 (m, br), 138.0 (m, br), 143.6 (m, br), 145.9 (m, br), 147.7 (m, br), 148.4 (br) (*C*$_6$F$_5$ and B(*C*$_6$F$_5$)$_2$). ^{19}F NMR (376 MHz, 298, C_6D_6, ppm): δ = –127.2 (m, 4 F, *o*-F_{BC6F5}), (–135.4, –136.3) (m, 2 F, *o*-F_{CC6F5}), –149.5 (m, 1 F, *p*-F_{CC6F5}), –155.5 (t, $^3J_{FF}$ = 20.9 Hz, 2 F, *p*-F_{BC6F5}), (–161.3, –163.3) (m, 2 F, *m*-F_{CC6F5}), –163.0 (m, 4 F, *m*-F_{BC6F5}). ^{11}B NMR (128 MHz, 298 K, C_6D_6, ppm): δ = 63.8. Anal. calcd (%) for $C_{59}H_{47}AlBF_{15}N_2S_2$ (M_r =1170.93): C, 60.52; H, 4.05; N, 2.39; S, 5.48. Found: C, 60.35; H, 4.16; N, 2.14; S, 5.42.

L^{2}AlC(Ph)=C(C$_6$F$_5$)B(C$_6$F$_5$)$_2$(C≡CPh) (L^2 = *o*-C$_6$H$_4$(CH=NAr)NAr, Ar = 2,6-*i*Pr$_2$C$_6$H$_3$, **7**) At room temperature, toluene (20 mL) was added to a mixture of **3** (134 mg, 0.2 mmol) and $B(C_6F_5)_3$ (100 mg, 0.2 mmol). This solution was stirred at room temperature for 5 h, then the solution was concentrated to ca. 5 mL and overlaid with 10 mL of n-hexane, then stored at -20 ℃ for 2 days in a freezer, light yellow crystals of **7** were obtained which were collected by filtration then washed with n-hexane. Yield：142 mg, 53%. Mp: 296 ℃ (dec.). ^1H NMR (400 MHz, C_6D_6, 298 K, ppm): δ = 0.08 (d, $^3J_{HH}$ = 6.3 Hz, 3 H, CH*Me*$_2$), 0.42 (d, $^3J_{HH}$ = 6.3 Hz, 3 H, CH*Me*$_2$), 0.61 (d, $^3J_{HH}$ = 6.3 Hz, 3 H, CH*Me*$_2$), 0.64 (d, $^3J_{HH}$ = 6.3 Hz, 3 H, CH*Me*$_2$), 0.65 (d, $^3J_{HH}$ = 6.3 Hz, 3 H, CH*Me*$_2$), 1.17 (d, $^3J_{HH}$ = 6.3 Hz, 3 H, CH*Me*$_2$), 1.30 (d, $^3J_{HH}$ = 6.3 Hz, 3 H, CH*Me*$_2$), 1.77 (d, $^3J_{HH}$ = 6.3 Hz, 3 H, CH*Me*$_2$), 2.40 (m, 1 H, C*H*Me$_2$), 2.93 (m, 1 H, C*H*Me$_2$), 2.99 (m, 1 H, C*H*Me$_2$), 4.06 (m, 1 H, C*H*Me$_2$), 6.10−7.40 (m, 20 H) (C$_6$*H*$_3$, C$_6$*H*$_4$ and C$_6$*H*$_5$), 7.88 (s, 1 H, *H*C=N). $^{13}C\{^1H\}$ NMR (100 MHz, C_6D_6, 298 K, ppm): δ = 21.2, 23.1

(d, J = 9.3 Hz), 24.5, 24.6, 26.3,26.6, 27.6 (CHMe_2), 28.6, 29.5, 29.9, 31.5 (CHMe$_2$), 118.3 (PhC≡), 123.9 (≡CB(C_6F_5)$_2$), 129.6 (AlC=), 158.1 (=CB(C_6F_5)$_2$), 117.2, 118.5, 121.6, 124.8, 124.9, 125.3, 126.0, 126.7, 129.9, 130.8, 132.1, 135.8, 137.0, 141.3, 141.5, 141.6, 143.0 145.7, 146.7, 159.0 (C_6H_3, C_6H_4 and C_6H_5), 175.8 (HC=N), 114.8 (m), 120.6 (m, br), 135.9 (m, br), 138.3 (m, br), 140.0 (m, br), 144.5 (m, br), 147.5 (m, br), 149.0 (m, br), 149.9 (m, br) (C_6F_5 and B(C_6F_5)$_2$). ^{19}F NMR (376 MHz, 298, C_6D_6, ppm): δ = –125.9 (br, 4 F, o-F_{BC6F5}), (–136.3, –137.1) (m, 2 F, o-F_{CC6F5}), –159.4 (m, 1 F, p-F_{CC6F5}), –159.7 (t, $^3J_{FF}$ = 20.7 Hz, 2 F, p-F_{BC6F5}), (–163.7, –164.9) (m, 2 F, m-F_{CC6F5}), (–165.3, 166.4) (m, 4 F, m-F_{BC6F5}). ^{11}B NMR (128 MHz, 298 K, C_6D_6, ppm): δ = -20.7. Anal. calcd (%) for $C_{65}H_{49}AlBF_{15}N_2$ (M_r =1180.89): C, 66.11; H, 4.18; N, 2.37. Found: C, 66.34; H, 4.29; N, 2.28.

$L^3Al(C{\equiv}CPh)_2$ (L^3 = tBuC(NCy)$_2$, Cy = $cyclo$-C_6H_{11}, **8**) A suspension of PhCCLi, freshly prepared from the reaction of PhCCH (1.022 g, 10 mmol) and nBuLi (4.17 mL 2.4 M n-hexane solution, 10 mmol) in toluene (30 mL) from –20 °C to room temperature in the period of 10 h, was added drop by drop to a solution of L^3AlCl_2 (1.8 g, 5 mmol) in toluene (30 mL) at room temperature under stirring. After addition, the mixture was allowed to heat at reflux for 24 h. Then the reaction mixture was cooled to room temperature, from which the insoluble LiCl was removed. The filtrate was evaporated to dryness under reduced pressure, producing an off-white solid of **8** that was washed with cooled n-hexane (10 mL at –20 °C). Yield: 2.0 g, 81%. Mp: 168 °C. ^{1}H NMR (400 MHz, C_6D_6, 298 K, ppm): δ = 0.90–2.10 (m, 20 H, CH(CH_2)$_5$), 1.07 (s, 9 H, CMe_3), 3.53 (m, 2 H, CH(CH$_2$)$_5$), 6.88–6.95 (m), 7.57–7.60 (m) (10 H, C_6H_5). ^{13}C{^{1}H} NMR (100 MHz, C_6D_6, 298 K, ppm): δ = 25.6, 25.8, 29.1 (CH(CH$_2$)$_5$), 37.2 (CMe_3), 39.8 (CMe$_3$), 54.5 (CH(CH$_2$)$_5$), 102.7 (AlC≡), 108.0 (PhC≡), 125.7, 128.4, 132.5, 137.9 (C_6H_5), 182.7 (NCN). Anal. calcd (%) for $C_{33}H_{41}AlN_2$ (M_r = 492.69): C, 80.45; H, 8.39; N, 5.69. Found: C, 80.18; H, 8.54; N, 5.48.

$L^3AlC(Ph){=}C(C_6F_5)B(C_6F_5)_2(C{\equiv}CPh)$ (L^3 = tBuC(NCy)$_2$, Cy = $cyclo$-C_6H_{11}, **9**) At room temperature, toluene (20 mL) was added to a mixture of **8** (98 mg, 0.2 mmol) and $B(C_6F_5)_3$ (100 mg, 0.2 mmol). This solution was stirred at this temperature for 12 h. Then the solution was concentrated to ca. 5 mL. By storing at 4 °C for 2 days, colorless crystals **9** were collected by filtration then washed with n-hexane. Yield: 134 mg, 67%. Mp: 186 °C (dec.). ^{1}H NMR (400 MHz, C_6D_6, 298 K, ppm): δ = 0.65 (s, 9 H, CMe_3), 0.90–1.80 (m, 20 H, NCH(CH_2)$_5$), 3.26 (m, 2 H, NCH(CH$_2$)$_5$), 7.32–6.73 (m, 10 H, C_6H_5). ^{13}C{^{1}H} NMR (100 MHz, C_6D_6, 298 K, ppm): δ = 24.7, 25.1, 25.3, 27.9 (CMe_3), 35.2, 36.9, 39.1 (CMe$_3$), 54.3 (NCH(CH$_2$)$_5$), 118.2 (PhC≡), 123.2 (≡CB(C_6F_5)$_2$), 131.7 (AlC=), 159.0 (=CB(C_6F_5)$_2$), 125.7, 126.3, 128.0, 128.3, 132.0, 133.3, 145.6 (C_6H_5) 187.0 (NCN). 120.0 (m, br), 135.8 (m, br), 138.3 (m, br), 141.8 (m, br), 144.2 (m, br), 147.3 (m, br), 149.7 (m, br) (C_6F_5 and B(C_6F_5)$_2$). ^{19}F NMR (376 MHz, 298, C_6D_6, ppm): δ = –129.4 (m, 4 F, o-F_{BC6F5}), –138.9 (m, 2 F, o-F_{CC6F5}), –158.4 (t, $^3J_{FF}$ = 21.4 Hz, 1 F, p-F_{CC6F5}), –159.0 (t, $^3J_{FF}$ = 21.0 Hz, 2 F, p-F_{BC6F5}), –164.7 (m, 2 F, m-F_{CC6F5}), –164.9 (m, 4 F, m-F_{BC6F5}). ^{11}B NMR (128 MHz, 298 K, C_6D_6, ppm): δ = –20.2. Anal. calcd (%) for $C_{51}H_{41}AlBF_{15}N_2$ (M_r = 1004.67): C, 60.97; H, 4.11; N, 2.79. Found: C, 61.26; H, 4.24; N, 2.36.

II. NMR spectra-detected reactions

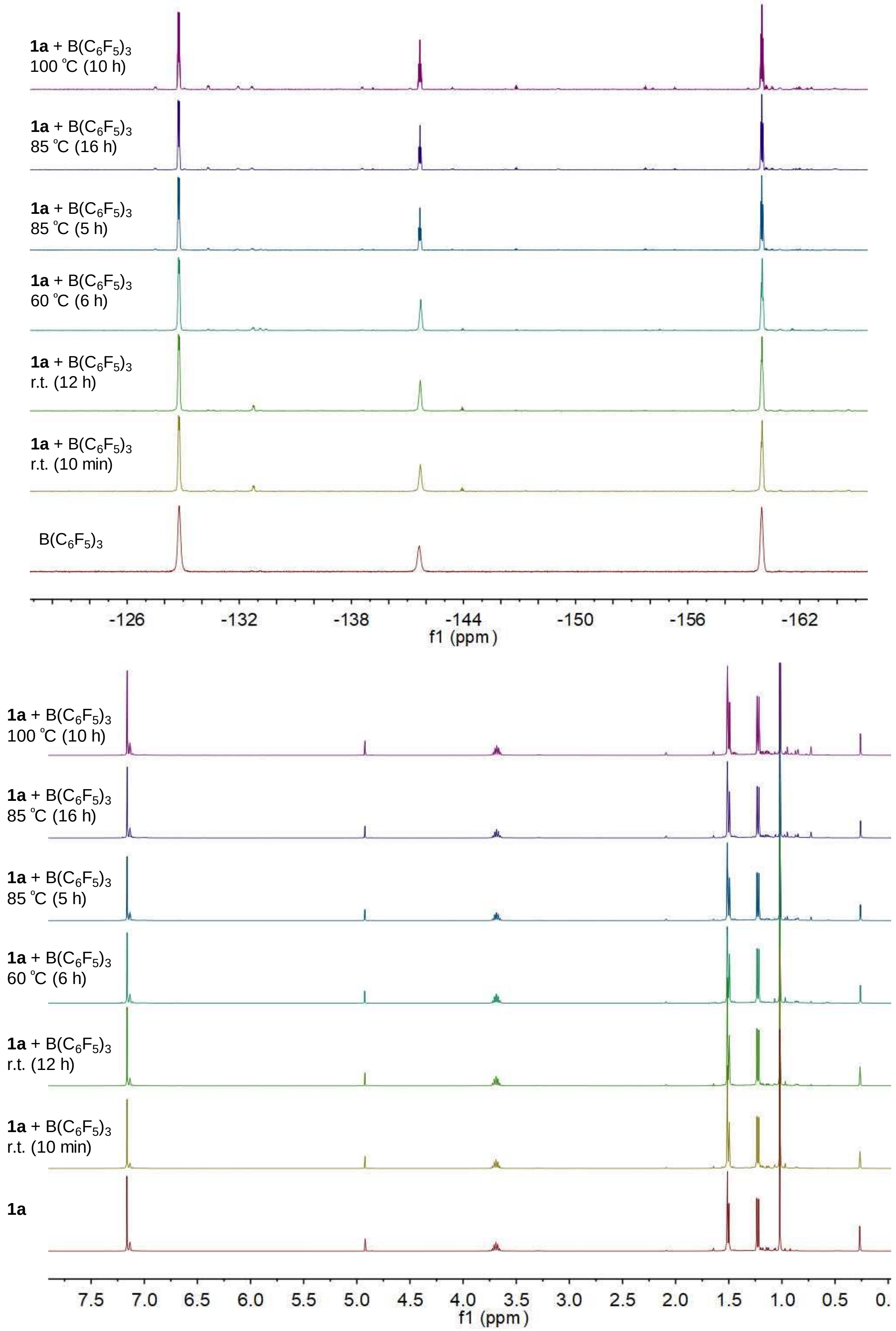

Figure S2-1. ^{19}F NMR (up) and ^{1}H NMR (down) spectra timely traced for reaction of **1a** and equivalent $B(C_6F_5)_3$ in C_6D_6.

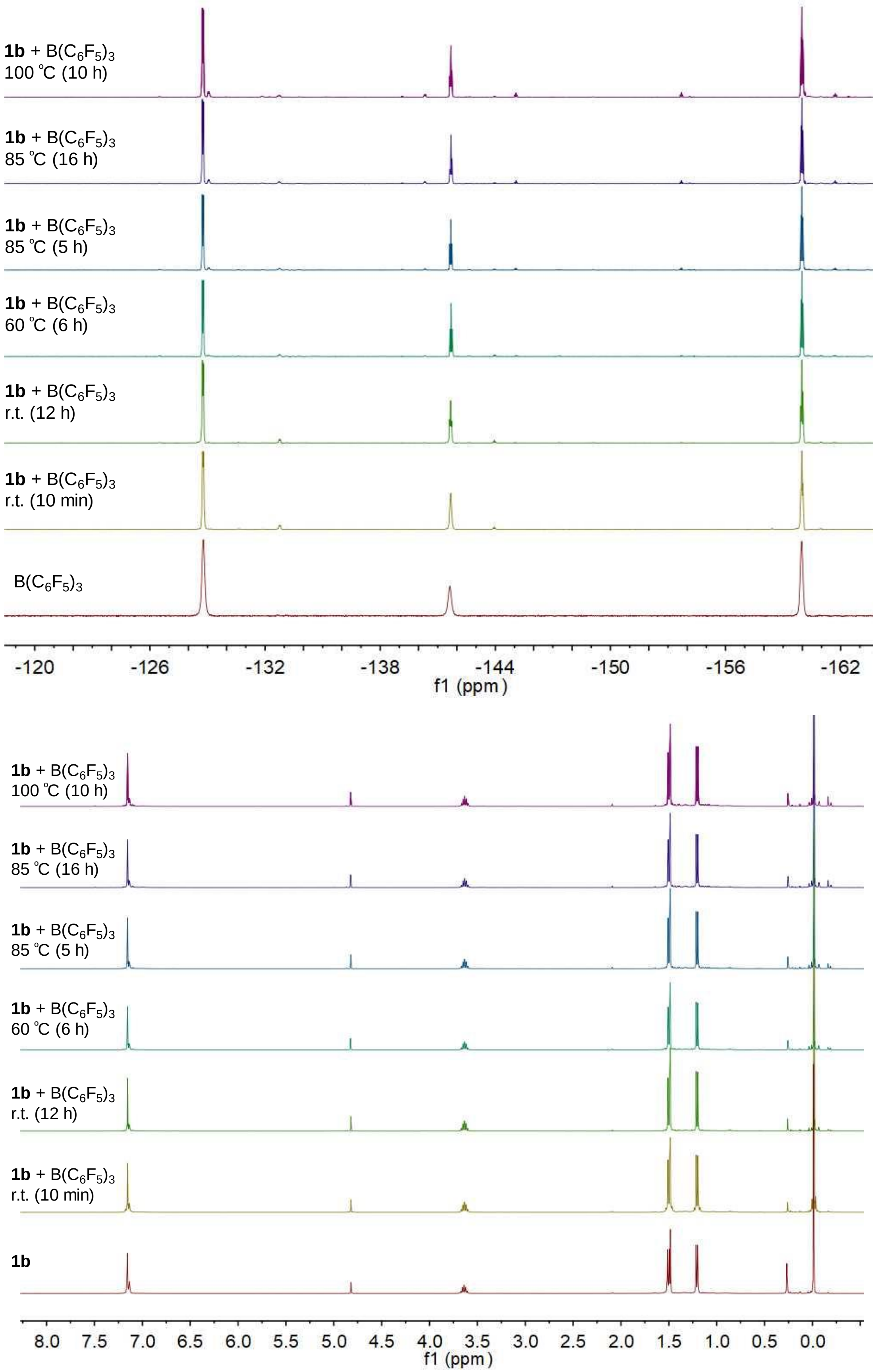

Figure S2-2. ^{19}F NMR (up) and ^{1}H NMR (down) spectra timely traced for reaction of **1b** and equivalent $B(C_6F_5)_3$ in C_6D_6.

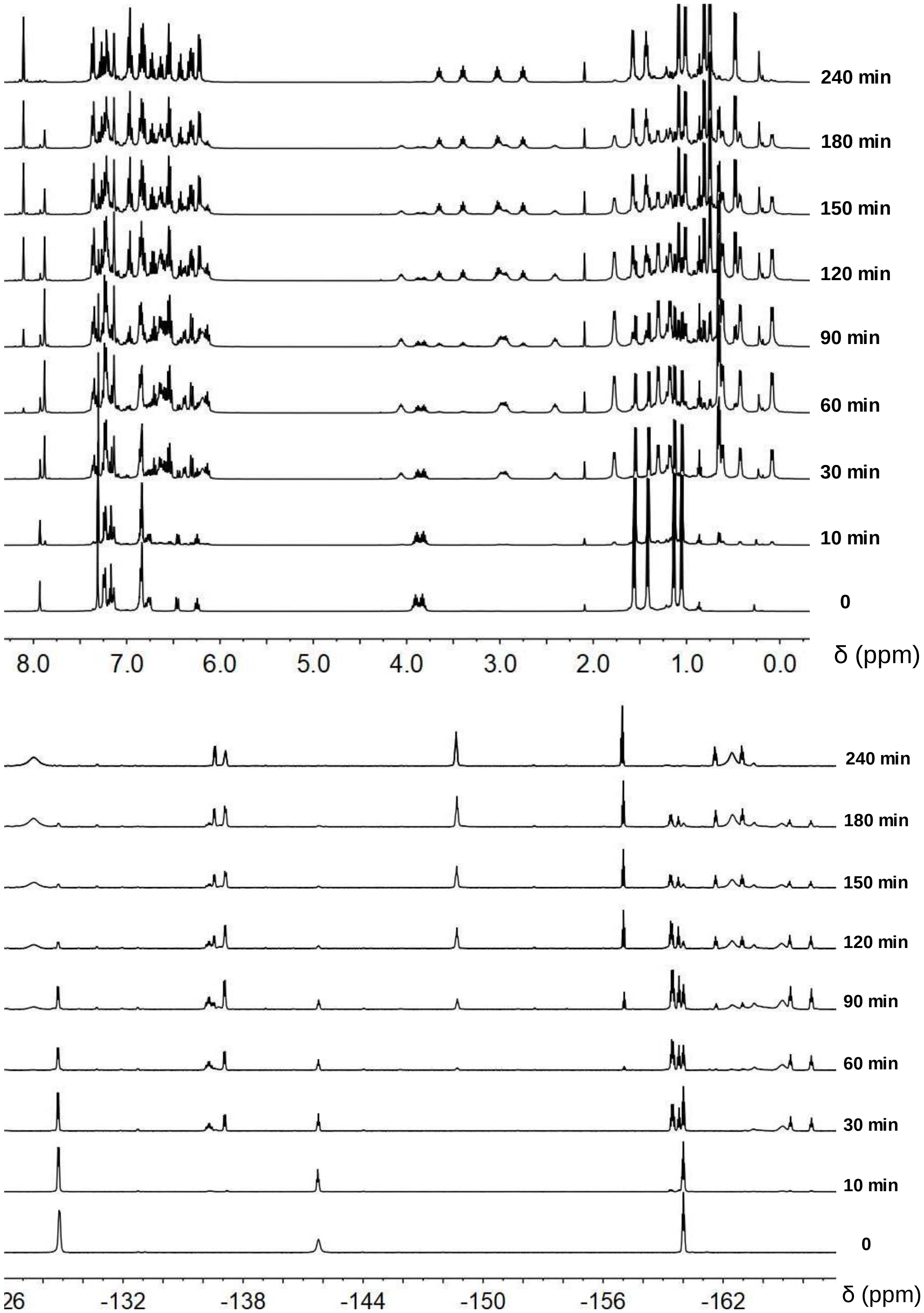

Figure S2-3. ^{1}H NMR (up) and ^{19}F NMR (down) spectra timely traced for reaction of **3** and equivalent $B(C_6F_5)_3$ in C_6D_6 at 65 ℃.

III. X-Ray crystallographic details

X-ray Crystallographic Analysis Crystallographic data for compounds **2**, **4** $\cdot 0.5C_6H_6$, **6** $\cdot C_6H_6$, **7** $\cdot$1.25toluene and **9** were collected on an Oxford Gemini S Ultra system. During measurements a graphite-monochromatic Cu-Kα radiation (λ = 1.54178 Å) was applied for **2**, **4** $\cdot 0.5C_6H_6$, **6** $\cdot C_6H_6$ and **7** $\cdot 1.45C_6H_5$, and the Mo-Kα radiation (λ = 0.71073 Å) was used for **9**. Absorption corrections were all employed using the spherical harmonics program (multi-scan type). All the structures were solved by direct methods (SHELXS-97)[S5] and refined against F^2 using SHELXL-2014.[S6] In general, the non-hydrogen atoms were located by difference Fourier synthesis and refined anisotropically, and hydrogen atoms were included using a riding mode with Uiso tied to the Uiso of the parent atoms unless otherwise specified. In **4** $\cdot 0.5C_6H_6$, half benzene was determined. In **6** $\cdot C_6H_6$, two half benzene were determined and located in disorder, which were both treated by the PART method. For the one half C_6H_6 molecular, the final refinement gave two parts as C(62)C(63)C(64) with occupation of 0.83241 and C(62A)C(63A)C(64A) with occupation of 0.16759. For the another half C_6H_6 molecular, the final refinement gave two parts as C(65)C(66)C(67) with occupation of 0.81751 and C(65A)C(66A)C(67A) with occupation of 0.18249. In **7** $\cdot$1.25 toluene, one toluene was located in disorder and treated by the PART method, where final refinement gave two parts as C(71)C(72)C(73)C(74)C(75)C(76)(C77) with occupation of 0.65853 and C(71A)C(72A)C(73A)C(74A)C(75A)C(76A)(C77A) with occupation of 0.34147. A summary of cell parameters, data collection, and structure solution and refinements is given in Table S3-1. CCDC-1966872 (**2**), -1966873 (**4** $\cdot 0.5C_6H_6$), -1966874 (**6** $\cdot C_6H_6$), -1966875 (**7** $\cdot$1.25toluene), and -1966876 (**9**) contain the supplementary crystallographic data for this paper. These data can be obtained free of charge at www.ccdc.cam.ac.uk/conts/retrieving.html

Table S3-1. Crystal data and refinements

	**2**	**4** 0.5 C_6H_6	**6** C_6H_6
Empirical formula	$C_{63}H_{51}AlBF_{15}N_2$	$C_{68}H_{52}AlBF_{15}N_2$	$C_{67}H_{51}AlBF_{15}N_2S_2$
formula weight	1158.85	1219.91	1271.01
crystal system	monoclinic	triclinic	triclinic
space group	*Cc*	*P-1*	*P-1*
a/Å	11.9379(3)	12.3832(6)	12.0881(4)
b/Å	26.3271(6)	12.9434(5)	12.4312(4)
c/Å	17.9162(4)	18.2397(7)	21.1569(6)
α/deg	90	94.726(3)	102.037(3)
β/deg	97.694(2)	90.688(3)	93.533(2)
γ/deg	90	98.759(4)	101.957(3)
V/Å^3	5580.2(2)	2878.7(2)	3023.56(16)
Z	4	2	2
ρ_{calcd}/g·cm^{-3}	1.379	1.407	1.396
μ/mm^{-1}	1.131	1.127	1.725
F(000)	2384	1254	1304
crystal size/mm^3	0.40×0.20×0.40	0.40×0.40×0.40	0.40×0.20×0.20
θ range/deg	3.36–62.09	3.47–65.76	3.73–62.17
index ranges	$-8 \le h \le 13$	$-14 \le h \le 12$	$-13 \le h \le 13$
	$-27 \le k \le 29$	$-15 \le k \le 15$	$-14 \le k \le 14$
	$-20 \le l \le 16$	$-21 \le l \le 17$	$-23 \le l \le 24$
collected data	9179	18375	24411
unique data	5077 (R_{int} = 0.0265)	9679 (R_{int} = 0.0329)	9485 (R_{int} = 0.0353)
completeness to θ	98.9	96.9	99.2
data/restraints/parameters	5077/2/749	9679/0/792	9485/200/857
GOF on F^2	1.016	1.032	1.039
final R indices [$I > 2$ (I)]	R_1 = 0.0325 wR_2 = 0.0851	R_1 = 0.0406 wR_2 = 0.1055	R_1 = 0.0539 wR_2 = 0.1523
R indices (all data)	R_1 = 0.0334 wR_2 = 0.0868	R_1 = 0.0457 wR_2 = 0.1109	R_1 = 0.0590 wR_2 = 0.1594
Largest diff peak/hole (e·Å^{-3})	0.183/–0.190	0.298/–0.298	0.759/–0.693

[a] $R_1 = \sum(||F_o|-|F_c||)/\sum|F_o|$, $wR_2 = [\sum w(F_o^2-F_c^2)^2/\sum w(F_o^2)]^{1/2}$, GOF $= [\sum w(F_o^2-F_c^2)^2/(N_o-N_p)]^{1/2}$.

(continued)

	**7**·1.25 toluene	**9**
Empirical formula	$C_{73.75}H_{58.50}AlBF_{15}N_2$	$C_{51}H_{41}AlBF_{15}N_2$
formula weight	1295.52	1004.65
crystal system	monoclinic	monoclinic
space group	*C*2/c	*P*2(1)/n
a/Å	48.5599(9)	12.3222(6)
b/Å	13.4073(3)	15.3284(8)
c/Å	22.3296(4)	25.2138(13)
α/deg	90	90
β/deg	117.127(2)	95.065(4)
γ/deg	90	90
V/Å^3	12938.6(4)	4743.8(4)
Z	8	4
ρ_{calcd}/g·cm^{-3}	1.330	1.407
μ/mm^{-1}	1.035	0.140
F(000)	5344	2056
crystal size/mm^3	0.24 x 0.21 x 0.18	0.30×0.20×0.20
θ range/deg	3.45–62.16	3.06–26.00
index ranges	$-52 \le h \le 55$<br>$-15 \le k \le 15$<br>$-25 \le l \le 25$	$-14 \le h \le 15$<br>$-18 \le k \le 16$<br>$-30 \le l \le 31$
collected data	48579	22322
unique data	10183 (R_{int} = 0.0413)	9305 (R_{int} = 0.1097)
completeness to θ	99.6	99.8
data/restraints/parameters	10183/1/813	9305/0/634
GOF on F^2	1.040	0.996
final R indices [I > 2 (I)]	R_1 = 0.0617<br>wR_2 = 0.1801	R_1 = 0.0911<br>wR_2 = 0.1978
R indices (all data)	R_1 = 0.0639<br>wR_2 = 0.1926	R_1 = 0.1637<br>wR_2 = 0.2419
Largest diff peak/hole (e·Å^{-3})	0.604/–0.641	0.492/–0.378

[a] $R_1 = \sum(||F_o|-|F_c||)/\sum|F_o|$, $wR_2 = [\sum w(F_o^2-F_c^2)^2/\sum w(F_o^2)]^{1/2}$, GOF $= [\sum w(F_o^2-F_c^2)^2/(N_o-N_p)]^{1/2}$.

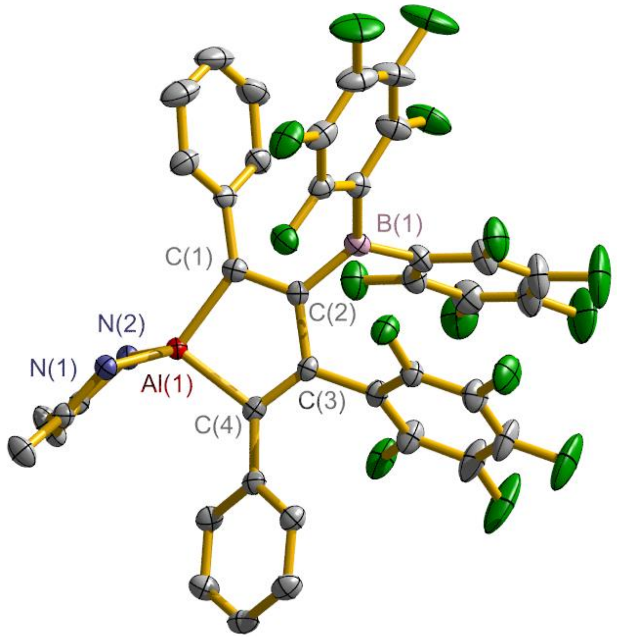

Figure S3-1. X-ray crystal structure of **2** with thermal ellipsoids at 50% probability level. H atoms and Ar groups are omitted for clarity. Selected bond lengths (Å) and angles (°): Al(1)-N(1) 1.897(2), Al(1)-N(2) 1.905(2), Al(1)-C(1) 1.979(3), Al(1)-C(4) 2.001(2), B(1)-C(2) 1.563(4), C(1)-C(2) 1.366(4), C(2)-C(3) 1.522(3), C(3)-C(4) 1.360(4); N(1)-Al(1)-N(2) 97.15(9), C(1)-Al(1)-C(4) 90.30(11), C(2)-C(1)-Al(1) 106.64(18), C(1)-C(2)-C(3) 118.4(2), C(4)-C(3)-C(2) 118.5(2), C(3)-C(4)-Al(1) 106.14(18).

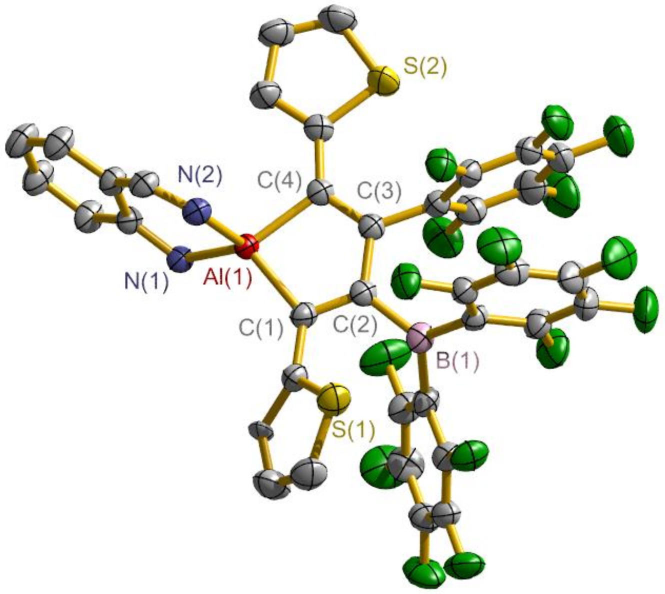

Figure S3-2. X-ray crystal structure of **6** with thermal ellipsoids at 50% probability level. H atoms and Ar groups are omitted for clarity. Selected bond lengths (Å) and angles (°): Al(1)-N(1) 1.8629(19), Al(1)-N(2) 1.9318(19), Al(1)-C(1) 1.972(2), Al(1)-C(4) 2.000(2), B(1)-C(2) 1.572(4), C(1)-C(2) 1.359(3), C(2)-C(3) 1.450(3), C(3)-C(4) 1.354(3); N(1)-Al(1)-N(2) 95.99(8), C(1)-Al(1)-C(4) 89.74(10),

C(2)-C(1)-Al(1) 107.15(16), C(1)-C(2)-C(3) 118.0(2), C(4)-C(3)-C(2) 119.2(2), C(3)-C(4)-Al(1) 105.84(16).

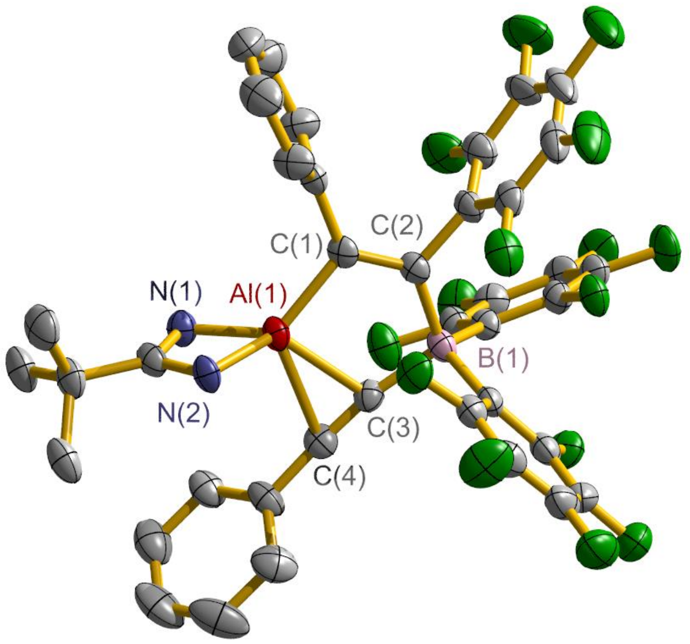

Figure S3-3. X-ray crystal structure of **9** with thermal ellipsoids at 50% probability level. H atoms and Cy groups are omitted for clarity. Selected bond lengths (Å) and angles (°): Al(1)−N(1) 1.876(3), Al(1)−N(2) 1.875(3), Al(1)−C(1) 1.939(4), C(1)−C(2) 1.354(5), B(1)−C(2) 1.619(6), B(1)−C(3) 1.632(6), C(3)−C(4) 1.217(5), Al(1)−C(3) 2.194(4), Al(1)−C(4) 2.352(5), N(1)−Al(1)−N(2) 70.54(15), C(3)−Al(1)−C(4) 28.5(1).

IV. The UV-vis absorption/emission spectra and luminescence lifetime measurements

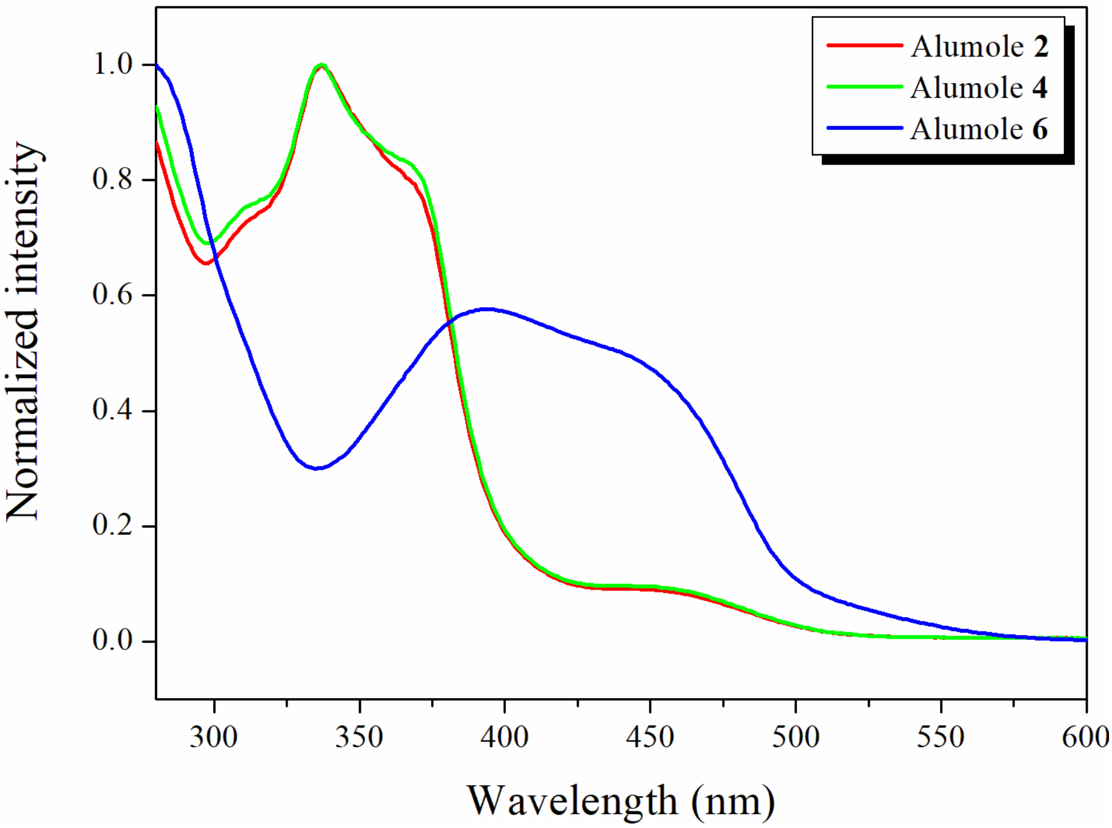

Figure S4-1. Normalized UV-vis absorption spectra of **2**, **4**, and **6** in benzene

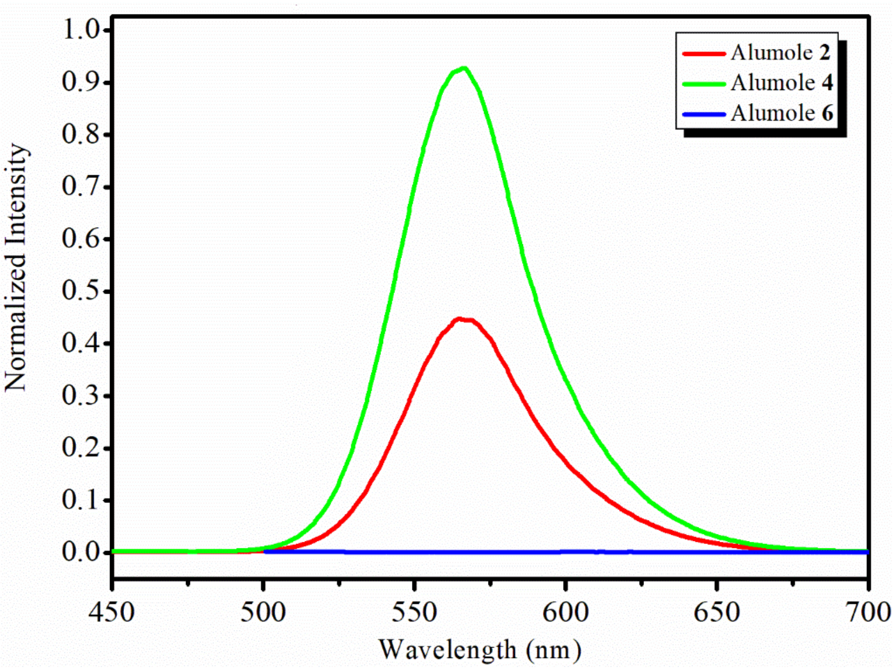

Figure S4-2. Normalized emission spectra of **2**, **4** and **6** in the solid state

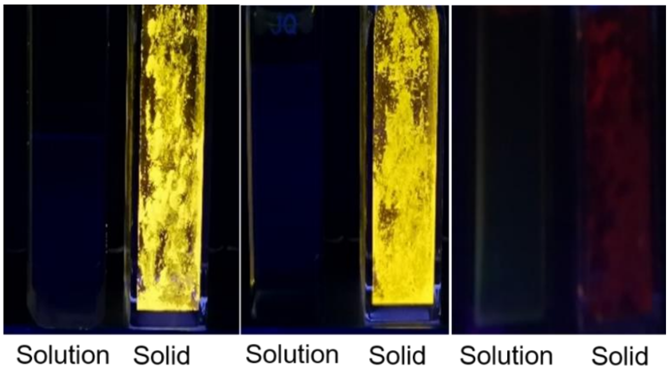

Figure S4-3. The solution (in benzene) and solid state emission pictures of **2**, **4** and **6** (from left to right, excitation at 365 nm)

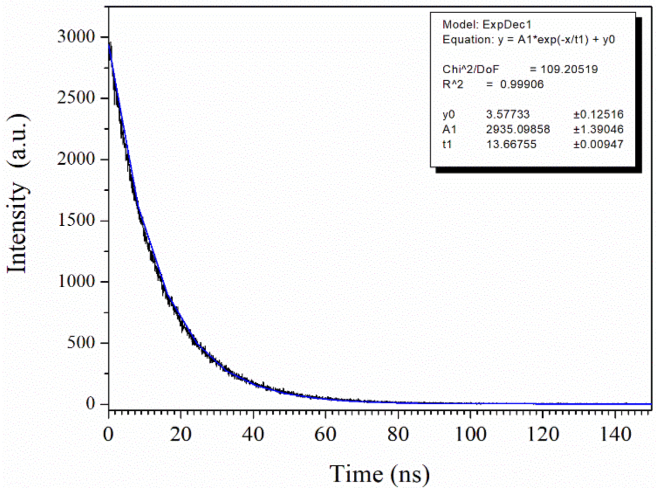

Figure S4-4. The emission decay curve of solid **2**.

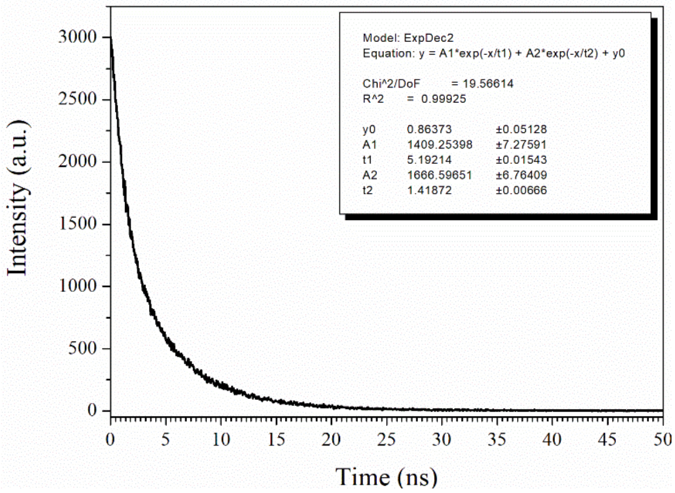

Figure S4-5. The emission decay curve of solid **4**.

V. Theoretical calculation study

Computational Details All structure optimizations were carried out by using M06-2X functional as implemented in the Gaussian 09 program.[S7] This functional is selected due to its good performance in studying thermochemistry, kinetics, and non-covalent interactions (such as van der Waals interactions) for main group elements.[S8] All structures were fully optimized at M06-2X/6-31G* level of theory. Harmonic frequency calculations were performed at the same level of theory as the optimizations to estimate the zero-point energies, as well as the thermal and entropic corrections. The single-point calculations were performed at large basis set of M06-2X/6-311G**, in conjunction with the SMD[S9] continuum solvation model (solvent, toluene). TD-DFT calculations were performed at B3LYP/6-31G* level of theory, in conjunction with the SMD solvation model (solvent, benzene).

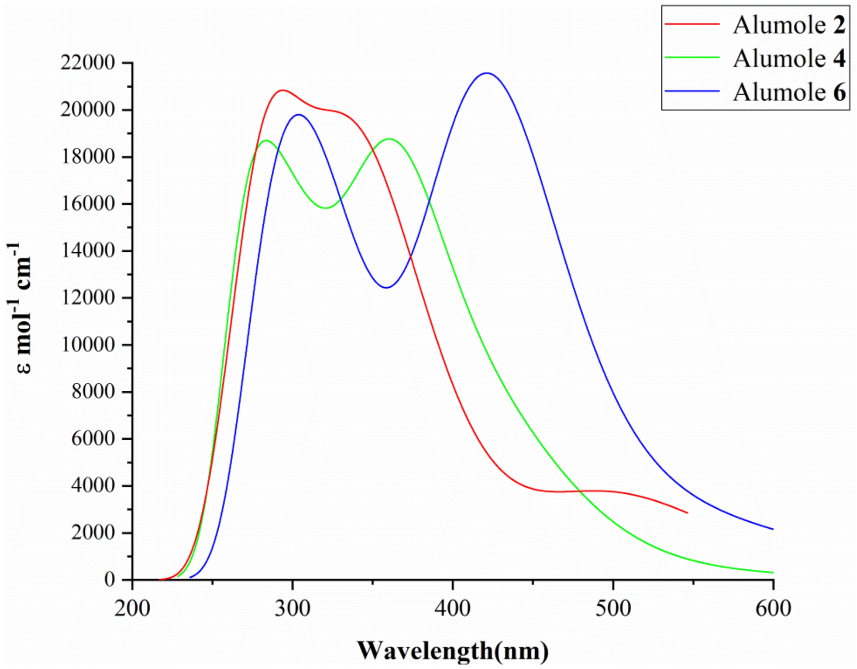

Figure S5-1. TD-DFT calculated UV–vis spectra of **2**, **4** and **6** in benzene.

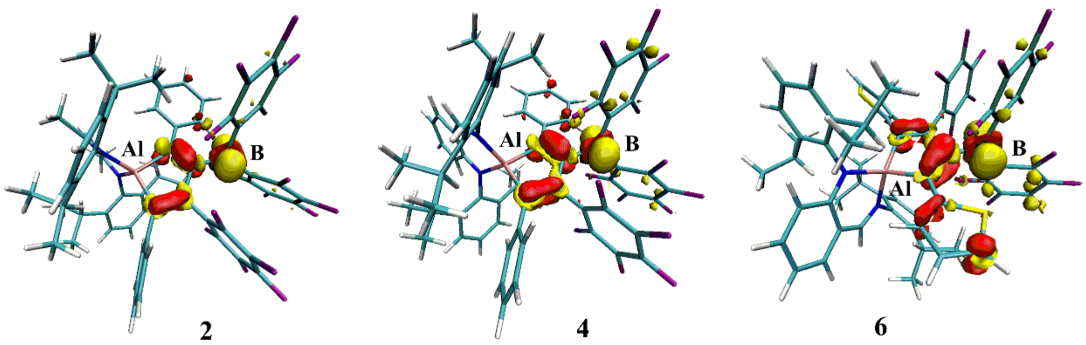

Figure S5-2. The calculated charge density configurations showing difference between the first lowest singlet excited state (S1) and the ground state (the loss in red and the gain in yellow) for **2**, **4** and **6**.

RC

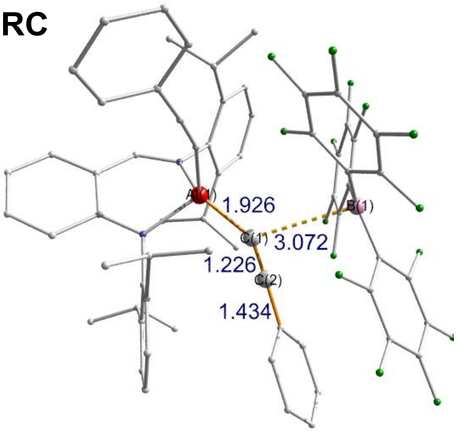

TS1

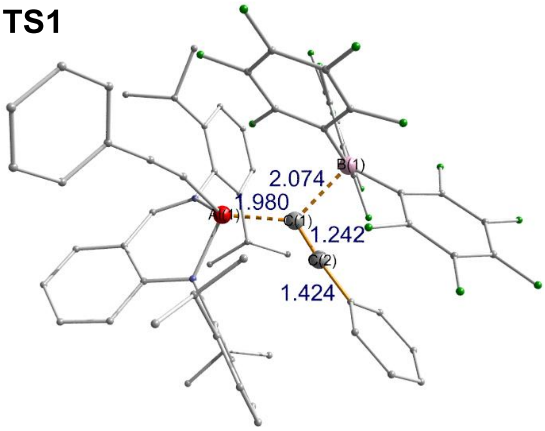

Int1

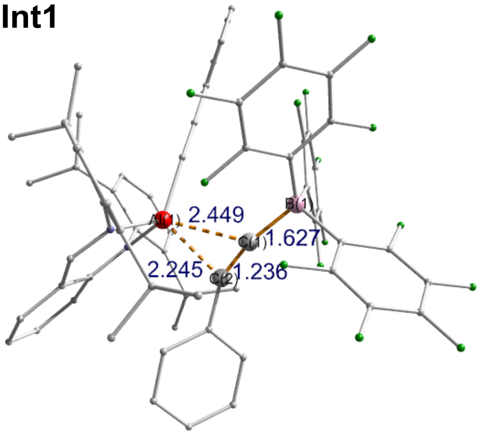

TS2

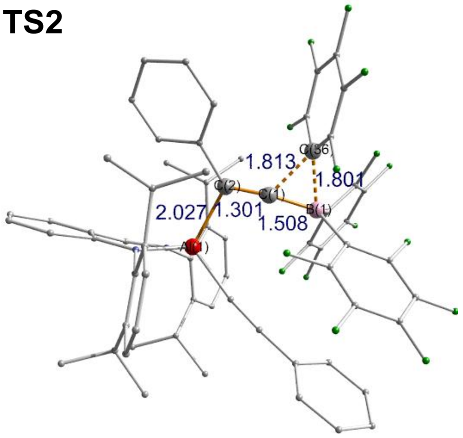

Int2

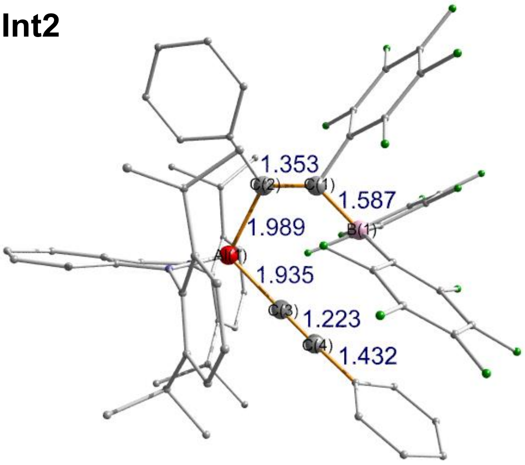

TS3

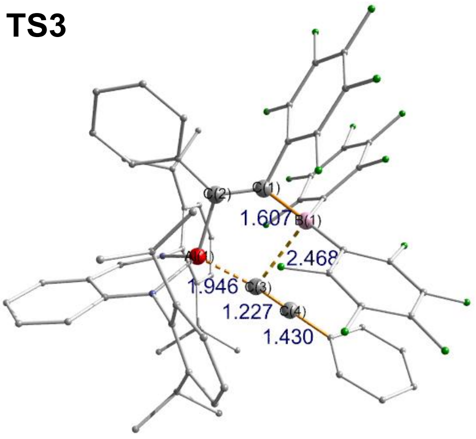

7

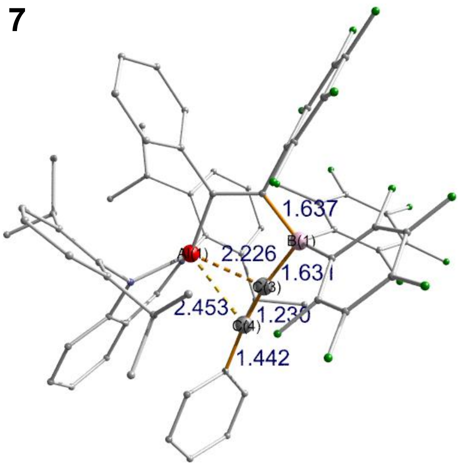

TS4

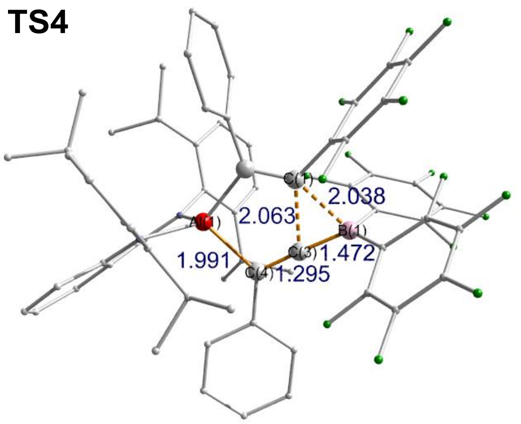

4

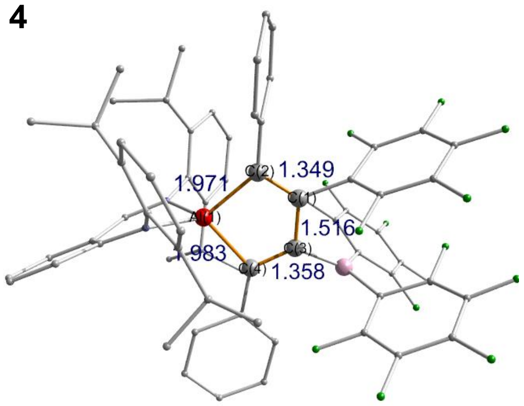

Figure S5-3. Optimized geometries of species involved in the alumole synthesis from aluminum dialkynyl and $B(C_6F_5)_3$ at the M06-2X/6-31G* level of theory. The key distances are given in angstrom.

Cartesian coordinates of all computed species, as well as the total Gibbs energies at M06-2X/6-311G**

RC

Total Gibbs energy= -4378.330193 a.u.

5	-2.323665000	-0.990343000	1.376534000
13	1.436046000	0.149333000	-0.587445000
9	-2.406637000	-0.281302000	4.224039000
9	-0.506369000	-0.823372000	5.994630000
9	1.763770000	-2.102262000	5.225235000
9	2.123376000	-2.796851000	2.638404000
9	0.269149000	-2.169182000	0.800217000
9	-3.974399000	-2.357898000	-3.104611000
9	-3.166173000	-4.912898000	-2.731408000
9	-1.905185000	-5.617593000	-0.422838000
9	-1.581825000	-3.860314000	1.516849000
9	-3.557201000	-0.526389000	-1.222350000

9	-5.198063000	-1.075657000	0.723321000
9	-6.891210000	0.951139000	1.008224000
9	-6.046989000	3.311278000	2.055426000
9	-3.462239000	3.598977000	2.848045000
9	-1.728112000	1.620330000	2.497390000
7	1.854974000	-1.109086000	-1.993633000
7	2.283888000	1.636195000	-1.384035000
6	-0.438859000	0.574802000	-0.476469000
6	-1.304394000	1.436549000	-0.581083000
6	3.484354000	-0.369607000	1.702473000
6	2.554646000	-0.336087000	0.910430000
6	2.968954000	-0.929772000	-2.644771000
1	3.253684000	-1.715098000	-3.351400000
6	3.847420000	0.198708000	-2.578773000
6	3.469528000	1.469951000	-2.027801000
6	4.381878000	2.544971000	-2.223748000
1	4.116201000	3.522791000	-1.837087000
6	5.587595000	2.357704000	-2.860317000
1	6.262224000	3.203376000	-2.962699000
6	5.965562000	1.103901000	-3.378379000
1	6.917653000	0.975251000	-3.879882000
6	5.087966000	0.057284000	-3.247151000
1	5.333940000	-0.917507000	-3.663032000
6	1.065166000	-2.262856000	-2.364131000
6	1.339404000	-3.511064000	-1.780521000
6	0.639861000	-4.616449000	-2.272495000
1	0.832383000	-5.598443000	-1.852782000
6	-0.300907000	-4.483096000	-3.284620000
1	-0.826505000	-5.357826000	-3.654129000
6	-0.602254000	-3.224857000	-3.792706000
1	-1.368776000	-3.129067000	-4.556402000
6	0.066084000	-2.085987000	-3.337514000
6	1.805853000	2.979498000	-1.200502000
6	2.027908000	3.661535000	0.011870000
6	1.535473000	4.962166000	0.149868000
1	1.700290000	5.493766000	1.083650000
6	0.847904000	5.584869000	-0.883656000
1	0.471589000	6.596226000	-0.760698000
6	0.644334000	4.904636000	-2.076967000
1	0.111387000	5.395163000	-2.887322000
6	1.113655000	3.602099000	-2.257970000
6	-1.886734000	3.686572000	0.115309000
1	-0.925819000	3.724644000	0.620722000
6	-2.771585000	4.755374000	0.178830000

1	-2.499901000	5.642763000	0.741931000
6	-4.009444000	4.678333000	-0.457518000
1	-4.707773000	5.506974000	-0.391813000
6	-4.347577000	3.538334000	-1.186264000
1	-5.305965000	3.482121000	-1.693670000
6	-3.465769000	2.466148000	-1.264256000
1	-3.724729000	1.582095000	-1.834014000
6	-2.235799000	2.526522000	-0.593983000
6	4.582616000	-0.301352000	2.622269000
6	4.628437000	-1.098237000	3.774262000
1	3.829633000	-1.804522000	3.967229000
6	5.692004000	-0.978929000	4.661352000
1	5.717173000	-1.597975000	5.552844000
6	6.718815000	-0.071134000	4.412817000
1	7.545734000	0.019226000	5.110415000
6	6.682703000	0.720211000	3.265935000
1	7.482305000	1.426996000	3.066040000
6	5.624208000	0.606728000	2.372941000
1	5.589989000	1.213047000	1.471781000
6	-1.157148000	-1.188378000	2.415318000
6	0.041357000	-1.818813000	2.074234000
6	1.019428000	-2.146256000	2.995822000
6	0.835220000	-1.797733000	4.326336000
6	-0.324207000	-1.145713000	4.717438000
6	-1.293893000	-0.860089000	3.767316000
6	-3.155919000	-1.774120000	-0.963084000
6	-3.385066000	-2.704820000	-1.962139000
6	-2.973424000	-4.014964000	-1.774126000
6	-2.339641000	-4.371626000	-0.595556000
6	-2.154095000	-3.416291000	0.392550000
6	-2.540366000	-2.078833000	0.254238000
6	-3.364799000	0.167747000	1.584051000
6	-4.712042000	0.064224000	1.223760000
6	-5.619756000	1.101403000	1.373812000
6	-5.194005000	2.306248000	1.916579000
6	-3.874932000	2.450260000	2.319845000
6	-2.993687000	1.395770000	2.141530000
6	2.374495000	-3.691686000	-0.680112000
1	2.476886000	-2.744938000	-0.138956000
6	3.746920000	-4.053071000	-1.264777000
1	4.143300000	-3.260064000	-1.906813000
1	3.681058000	-4.970150000	-1.861308000
1	4.469091000	-4.218634000	-0.459135000
6	1.947273000	-4.758681000	0.334526000

1	0.922552000	-4.594641000	0.683069000
1	2.614450000	-4.733277000	1.199428000
1	2.005955000	-5.765478000	-0.092937000
6	-0.225191000	-0.718305000	-3.938159000
1	0.102896000	0.044070000	-3.222197000
6	0.576510000	-0.527657000	-5.232421000
1	1.653790000	-0.632633000	-5.061205000
1	0.399017000	0.468428000	-5.652912000
1	0.281284000	-1.272836000	-5.980017000
6	-1.717872000	-0.484721000	-4.175760000
1	-2.269295000	-0.507240000	-3.232485000
1	-2.156359000	-1.225869000	-4.850545000
1	-1.872046000	0.497731000	-4.629236000
6	2.768994000	3.016108000	1.167360000
1	3.248284000	2.108860000	0.791601000
6	1.779555000	2.604468000	2.265879000
1	0.976939000	1.970536000	1.870625000
1	1.313714000	3.492742000	2.711001000
1	2.293395000	2.046900000	3.056372000
6	3.870148000	3.914401000	1.739516000
1	4.573033000	4.225163000	0.959164000
1	4.428620000	3.370691000	2.509187000
1	3.461021000	4.817370000	2.205515000
6	0.893451000	2.917190000	-3.595973000
1	1.240794000	1.882131000	-3.508981000
6	-0.591275000	2.886580000	-3.970123000
1	-1.173653000	2.331431000	-3.227174000
1	-0.726582000	2.411233000	-4.948192000
1	-1.009396000	3.896671000	-4.038242000
6	1.719807000	3.595410000	-4.696262000
1	2.785286000	3.595860000	-4.446162000
1	1.402326000	4.635124000	-4.836543000
1	1.590502000	3.072287000	-5.650257000

int1

Total Gibbs energy= -4378.311229 a.u.

5	2.247024000	0.072012000	-0.235343000
13	-1.380829000	-0.410874000	0.077115000
9	4.722449000	0.882654000	-1.850905000
9	6.495911000	-0.800989000	-2.877067000
9	6.218202000	-3.484383000	-2.554543000
9	4.088676000	-4.461300000	-1.144609000
9	2.302725000	-2.783540000	-0.088272000
9	0.895779000	-1.624126000	4.306820000

9	3.168143000	-0.600456000	5.429139000
9	4.966884000	0.711089000	3.868812000
9	4.498110000	1.016980000	1.232566000
9	0.467864000	-1.382903000	1.682802000
9	2.327927000	2.626619000	1.436343000
9	2.077599000	5.072677000	0.482663000
9	1.845451000	5.511660000	-2.197786000
9	1.923022000	3.389122000	-3.917273000
9	2.229097000	0.908996000	-2.975640000
7	-2.195517000	-1.942291000	0.970801000
7	-2.884456000	0.013520000	-0.976004000
6	-0.623501000	2.245048000	1.487472000
6	-0.861642000	1.124641000	1.064545000
6	0.887233000	-0.596325000	-0.826786000
6	0.032079000	-1.212165000	-1.473164000
6	-3.329678000	-2.436224000	0.537197000
1	-3.730693000	-3.272229000	1.115646000
6	-4.117457000	-2.044506000	-0.580533000
6	-3.916266000	-0.821297000	-1.287806000
6	-4.875239000	-0.500791000	-2.286796000
1	-4.785746000	0.444061000	-2.808195000
6	-5.897379000	-1.366152000	-2.601863000
1	-6.590315000	-1.089351000	-3.391480000
6	-6.070058000	-2.591089000	-1.926337000
1	-6.885550000	-3.256127000	-2.185557000
6	-5.196253000	-2.902012000	-0.916513000
1	-5.315418000	-3.822044000	-0.348393000
6	-1.765695000	-2.592959000	2.208630000
6	-1.028171000	-3.784222000	2.126525000
6	-0.770929000	-4.459413000	3.319684000
1	-0.202222000	-5.383275000	3.295769000
6	-1.215212000	-3.961917000	4.539724000
1	-1.007051000	-4.507128000	5.455029000
6	-1.891316000	-2.751995000	4.590516000
1	-2.200859000	-2.349169000	5.549972000
6	-2.166450000	-2.031650000	3.425553000
6	-3.086427000	1.405911000	-1.320107000
6	-2.682242000	1.911914000	-2.565530000
6	-2.874499000	3.272665000	-2.825647000
1	-2.549001000	3.679210000	-3.779491000
6	-3.488788000	4.100865000	-1.899034000
1	-3.636981000	5.153705000	-2.120046000
6	-3.935763000	3.571214000	-0.693054000
1	-4.442665000	4.217990000	0.017230000

6	-3.747511000	2.225551000	-0.379180000
6	0.305087000	3.874830000	3.096123000
1	0.722717000	3.054194000	3.670896000
6	0.491940000	5.192322000	3.492250000
1	1.054225000	5.408722000	4.395098000
6	-0.030363000	6.235234000	2.729199000
1	0.125073000	7.263922000	3.039781000
6	-0.743440000	5.959651000	1.564828000
1	-1.143667000	6.771727000	0.965662000
6	-0.939119000	4.644412000	1.161850000
1	-1.489477000	4.410889000	0.254726000
6	-0.413568000	3.592963000	1.927073000
6	-0.487530000	-1.962385000	-2.602261000
6	0.145580000	-1.779352000	-3.840020000
1	0.990329000	-1.100910000	-3.903098000
6	-0.326003000	-2.454224000	-4.958836000
1	0.163030000	-2.308920000	-5.916984000
6	-1.421070000	-3.312252000	-4.854919000
1	-1.789417000	-3.829722000	-5.735153000
6	-2.032221000	-3.514193000	-3.620346000
1	-2.875880000	-4.191142000	-3.526587000
6	-1.560439000	-2.847628000	-2.495064000
1	-2.017174000	-3.022075000	-1.529783000
6	3.391806000	-0.870957000	-0.954474000
6	3.315159000	-2.252266000	-0.797705000
6	4.234561000	-3.146950000	-1.324270000
6	5.316257000	-2.653148000	-2.038614000
6	5.451959000	-1.282795000	-2.203714000
6	4.503090000	-0.421624000	-1.661318000
6	1.586422000	-0.813210000	2.191368000
6	1.788608000	-0.977432000	3.555353000
6	2.940352000	-0.458889000	4.124990000
6	3.858338000	0.211993000	3.323929000
6	3.600665000	0.352015000	1.969664000
6	2.445196000	-0.125903000	1.358979000
6	2.246688000	1.628521000	-0.723791000
6	2.229437000	2.744656000	0.105577000
6	2.100433000	4.046938000	-0.367059000
6	1.987108000	4.273333000	-1.729340000
6	2.034880000	3.194038000	-2.601113000
6	2.175115000	1.917337000	-2.082504000
6	-0.500878000	-4.313345000	0.799986000
1	-0.324714000	-3.450203000	0.143989000
6	-1.506183000	-5.252617000	0.118150000

1	-2.466924000	-4.770137000	-0.093919000
1	-1.707229000	-6.116874000	0.761115000
1	-1.101388000	-5.617473000	-0.831754000
6	0.841541000	-5.036501000	0.955616000
1	1.543018000	-4.446091000	1.549866000
1	1.292983000	-5.201956000	-0.026205000
1	0.712887000	-6.013931000	1.432870000
6	-2.898393000	-0.701819000	3.495723000
1	-2.643675000	-0.125691000	2.600079000
6	-4.416407000	-0.919923000	3.513720000
1	-4.769564000	-1.409402000	2.597864000
1	-4.945010000	0.034608000	3.606899000
1	-4.700723000	-1.550299000	4.363752000
6	-2.445295000	0.147499000	4.686712000
1	-1.353408000	0.201471000	4.731317000
1	-2.810229000	-0.254190000	5.638078000
1	-2.836533000	1.164371000	4.585708000
6	-2.089704000	1.034238000	-3.653835000
1	-2.141057000	-0.007995000	-3.319132000
6	-0.622497000	1.396290000	-3.882090000
1	-0.037753000	1.178569000	-2.985958000
1	-0.507122000	2.460941000	-4.113320000
1	-0.200809000	0.825805000	-4.715512000
6	-2.873747000	1.136434000	-4.968380000
1	-3.939744000	0.931006000	-4.829615000
1	-2.479519000	0.410002000	-5.686492000
1	-2.777366000	2.131521000	-5.415632000
6	-4.340299000	1.672283000	0.908897000
1	-3.872143000	0.703275000	1.113563000
6	-4.092391000	2.575461000	2.119502000
1	-3.025714000	2.762721000	2.266745000
1	-4.488829000	2.102216000	3.024204000
1	-4.601712000	3.539559000	2.016965000
6	-5.846223000	1.425693000	0.729197000
1	-6.046997000	0.738247000	-0.098209000
1	-6.363598000	2.368713000	0.518705000
1	-6.275220000	0.999771000	1.642992000

int2

Total Gibbs energy= -4378.325014 a.u.

5	1.701830000	0.429409000	-0.025468000
13	-1.594069000	-0.452253000	0.059557000
9	2.342106000	-3.059505000	-0.362973000
9	4.647425000	-3.900731000	-1.474690000

9	5.845187000	-2.421985000	-3.410334000
9	4.717371000	-0.090704000	-4.237065000
9	2.399131000	0.746533000	-3.148397000
9	2.275814000	-2.605820000	3.887869000
9	4.982339000	-2.550451000	3.612863000
9	6.114537000	-1.071489000	1.631288000
9	4.561183000	0.363192000	-0.030591000
9	0.728628000	-1.195454000	2.221492000
9	3.529275000	2.307812000	1.373697000
9	3.728161000	4.885925000	0.875308000
9	2.168059000	6.053829000	-1.016813000
9	0.407593000	4.551088000	-2.444289000
9	0.207204000	1.940616000	-1.988092000
7	-2.483027000	-1.667389000	1.312900000
7	-3.176704000	-0.007060000	-0.861939000
6	-0.407228000	2.029789000	1.598945000
6	-0.862477000	1.020006000	1.080446000
6	1.059973000	-0.631299000	-1.015628000
6	-0.170085000	-1.185184000	-1.119343000
6	-3.676965000	-2.135920000	1.070577000
1	-4.082181000	-2.830701000	1.811981000
6	-4.534847000	-1.838170000	-0.029048000
6	-4.316778000	-0.734806000	-0.915951000
6	-5.376064000	-0.422410000	-1.814240000
1	-5.265896000	0.443487000	-2.457278000
6	-6.509893000	-1.197736000	-1.882413000
1	-7.281306000	-0.934462000	-2.601054000
6	-6.698833000	-2.313965000	-1.041607000
1	-7.601163000	-2.910626000	-1.107145000
6	-5.726338000	-2.600742000	-0.118728000
1	-5.856547000	-3.426418000	0.577569000
6	-1.931719000	-2.051591000	2.599659000
6	-1.145464000	-3.212030000	2.685893000
6	-0.701487000	-3.598456000	3.949353000
1	-0.084860000	-4.485507000	4.050485000
6	-1.006797000	-2.846129000	5.077435000
1	-0.650062000	-3.164109000	6.052296000
6	-1.738443000	-1.673702000	4.954439000
1	-1.944142000	-1.077354000	5.837710000
6	-2.207811000	-1.241876000	3.710688000
6	-3.148958000	1.295596000	-1.475113000
6	-2.651894000	1.470352000	-2.779741000
6	-2.521906000	2.776140000	-3.263601000
1	-2.125914000	2.934175000	-4.263032000

6	-2.882086000	3.871169000	-2.492579000
1	-2.755923000	4.876160000	-2.883795000
6	-3.428638000	3.676878000	-1.227888000
1	-3.753494000	4.536716000	-0.647662000
6	-3.580608000	2.395298000	-0.699410000
6	1.172880000	3.290140000	3.014672000
1	1.523332000	2.353469000	3.438275000
6	1.766530000	4.496240000	3.365352000
1	2.581660000	4.507950000	4.081854000
6	1.330988000	5.686564000	2.783716000
1	1.807302000	6.624835000	3.050270000
6	0.294099000	5.671731000	1.852413000
1	-0.039663000	6.598027000	1.394713000
6	-0.300800000	4.468388000	1.489974000
1	-1.088776000	4.433426000	0.741067000
6	0.140151000	3.267535000	2.065905000
6	-0.514046000	-2.273185000	-2.082901000
6	0.137056000	-2.513964000	-3.305399000
1	0.960751000	-1.883384000	-3.615749000
6	-0.288203000	-3.518947000	-4.168809000
1	0.235267000	-3.668905000	-5.108602000
6	-1.383454000	-4.317029000	-3.847867000
1	-1.713181000	-5.097089000	-4.527003000
6	-2.061654000	-4.084138000	-2.655940000
1	-2.933353000	-4.675182000	-2.389846000
6	-1.633305000	-3.074713000	-1.802121000
1	-2.191046000	-2.907345000	-0.886407000
6	2.282644000	-1.140340000	-1.737295000
6	2.938054000	-0.396028000	-2.713848000
6	4.128706000	-0.817453000	-3.291905000
6	4.705720000	-2.006893000	-2.870414000
6	4.086462000	-2.766825000	-1.884423000
6	2.894430000	-2.325334000	-1.330991000
6	2.053225000	-1.097252000	2.079003000
6	2.837646000	-1.845284000	2.948351000
6	4.216254000	-1.831199000	2.800803000
6	4.793774000	-1.066407000	1.793956000
6	3.974838000	-0.319912000	0.961782000
6	2.588053000	-0.290044000	1.085919000
6	1.905567000	1.954468000	-0.338753000
6	2.776643000	2.783003000	0.378571000
6	2.892281000	4.147109000	0.148751000
6	2.099539000	4.744433000	-0.816843000
6	1.199187000	3.973978000	-1.542011000

6	1.120137000	2.613296000	-1.292901000
6	-0.740235000	-3.983724000	1.440083000
1	-0.608291000	-3.251019000	0.634619000
6	-1.811611000	-4.993172000	1.007390000
1	-2.775056000	-4.520951000	0.786783000
1	-1.975276000	-5.730848000	1.801201000
1	-1.488991000	-5.522300000	0.104512000
6	0.601496000	-4.703110000	1.598934000
1	1.361079000	-4.038917000	2.020148000
1	0.949754000	-5.050333000	0.622675000
1	0.513643000	-5.578352000	2.252110000
6	-2.990674000	0.056877000	3.587730000
1	-2.862887000	0.427973000	2.565930000
6	-4.489000000	-0.165951000	3.827803000
1	-4.918109000	-0.869244000	3.105375000
1	-5.033778000	0.780847000	3.737151000
1	-4.663916000	-0.566751000	4.832762000
6	-2.446651000	1.147668000	4.513920000
1	-1.368848000	1.275221000	4.373172000
1	-2.637141000	0.922397000	5.568726000
1	-2.933546000	2.101503000	4.291657000
6	-2.327722000	0.300607000	-3.698408000
1	-2.383223000	-0.623437000	-3.112726000
6	-0.926775000	0.393317000	-4.313144000
1	-0.145558000	0.358848000	-3.551607000
1	-0.802253000	1.321555000	-4.881808000
1	-0.774203000	-0.442483000	-5.003556000
6	-3.367555000	0.206409000	-4.826153000
1	-4.384117000	0.098052000	-4.438849000
1	-3.153256000	-0.660908000	-5.459173000
1	-3.338080000	1.102566000	-5.456655000
6	-4.295547000	2.205948000	0.631762000
1	-4.114856000	1.181031000	0.974997000
6	-3.812378000	3.151884000	1.733011000
1	-2.748685000	3.009892000	1.942718000
1	-4.372627000	2.956808000	2.654880000
1	-3.981458000	4.202510000	1.472059000
6	-5.810729000	2.369589000	0.432720000
1	-6.197065000	1.670188000	-0.314092000
1	-6.042185000	3.387674000	0.098653000
1	-6.340206000	2.192025000	1.375349000

7

Total Gibbs energy= -4378.341476 a.u.

13	-1.392951000	-0.043967000	0.081710000
7	-2.844315000	-1.162704000	-0.269353000
7	-2.457172000	1.516193000	0.536773000
6	-2.039529000	1.034296000	-3.104244000
6	-2.268036000	2.294222000	-3.676264000
6	-3.309015000	2.468002000	-4.580354000
1	-3.476189000	3.444715000	-5.023284000
6	-4.128129000	1.393668000	-4.921325000
1	-4.944507000	1.535798000	-5.622587000
6	-3.886699000	0.133367000	-4.377814000
1	-4.512500000	-0.712073000	-4.646697000
6	-2.840792000	-0.050216000	-3.483871000
1	-2.645112000	-1.026516000	-3.059636000
6	-3.640827000	1.651414000	0.007790000
1	-4.130160000	2.619022000	0.152837000
6	-4.401723000	0.667539000	-0.696707000
6	-5.616860000	1.111363000	-1.270523000
1	-5.840089000	2.175198000	-1.236348000
6	-6.500142000	0.236069000	-1.852734000
1	-7.423866000	0.588902000	-2.296622000
6	-6.192222000	-1.136922000	-1.827013000
1	-6.890778000	-1.850374000	-2.255374000
6	-5.021752000	-1.606820000	-1.271374000
1	-4.812301000	-2.670751000	-1.263107000
6	-4.053671000	-0.718784000	-0.732387000
6	-2.786683000	-2.554850000	0.118570000
6	-3.436602000	-4.278633000	1.676085000
1	-3.931423000	-4.580877000	2.595002000
6	-3.420510000	-2.929263000	1.322571000
6	-2.297180000	-3.531465000	-3.186553000
1	-3.296860000	-3.085716000	-3.124144000
1	-2.424063000	-4.618731000	-3.241264000
6	-4.076442000	-1.929713000	2.263768000
1	-3.868221000	-0.918358000	1.895888000
6	-5.599701000	-2.108698000	2.294976000
1	-6.035693000	-2.013926000	1.295894000
1	-5.865253000	-3.095568000	2.690930000
1	-6.057057000	-1.353155000	2.943338000
6	-3.489131000	-2.044762000	3.676524000
1	-2.417699000	-1.820075000	3.686900000
1	-3.999575000	-1.354392000	4.356481000
1	-3.619511000	-3.052829000	4.083078000
6	-1.941055000	2.629482000	1.311510000
6	-1.495594000	3.793993000	0.664659000

6	-0.941645000	4.802550000	1.457086000
1	-0.578572000	5.710591000	0.983269000
6	-0.839323000	4.666304000	2.831296000
1	-0.381007000	5.452987000	3.421748000
6	-1.331528000	3.525163000	3.451501000
1	-1.273798000	3.441997000	4.530841000
6	-1.896053000	2.488009000	2.711781000
6	-1.661767000	4.044180000	-0.823719000
1	-1.999010000	3.116152000	-1.289763000
6	-0.354280000	4.457133000	-1.508094000
1	-0.542309000	4.744217000	-2.548857000
1	0.102823000	5.323204000	-1.018808000
6	-2.746231000	5.104586000	-1.064783000
1	-3.689638000	4.843874000	-0.573262000
1	-2.430178000	6.077985000	-0.674036000
1	-2.937496000	5.216458000	-2.137927000
6	-2.544698000	1.303282000	3.412404000
1	-2.279708000	0.385099000	2.867952000
6	-2.085567000	1.127589000	4.861995000
1	-2.515372000	1.902999000	5.506274000
1	-2.419681000	0.157085000	5.239388000
6	-4.074310000	1.469845000	3.391990000
1	-4.490946000	1.454218000	2.380122000
1	-4.558533000	0.669785000	3.960374000
1	-4.345322000	2.425252000	3.854594000
5	1.509146000	0.453304000	-0.805863000
9	2.816030000	0.811986000	2.672447000
9	5.145656000	-0.111169000	3.594957000
9	6.211429000	-2.408690000	2.599160000
9	4.871116000	-3.789930000	0.668502000
9	2.518353000	-2.906974000	-0.235448000
9	4.402865000	0.249946000	-0.682738000
9	5.954133000	-1.438583000	-2.013602000
9	4.915378000	-3.025853000	-3.976421000
9	2.249950000	-2.864616000	-4.550655000
9	0.676856000	-1.233642000	-3.230101000
9	3.243365000	2.098281000	-2.573410000
9	4.165341000	4.530264000	-2.083636000
9	3.553642000	5.800799000	0.248381000
9	1.984549000	4.565500000	2.080309000
9	0.944700000	2.165404000	1.576416000
6	0.120729000	-0.774433000	1.065471000
6	1.325463000	-0.457892000	0.541772000
6	0.033834000	0.688765000	-1.461135000

6	-0.963895000	0.845868000	-2.163219000
6	0.023260000	-1.569241000	2.322271000
6	0.234088000	-0.928924000	3.550221000
1	0.471947000	0.133001000	3.545728000
6	0.170939000	-1.642421000	4.744776000
6	-0.290867000	-3.658303000	3.510701000
1	-0.495195000	-4.724982000	3.484513000
6	-0.233763000	-2.944936000	2.316224000
1	-0.384310000	-3.457576000	1.369481000
1	-1.614800000	3.121910000	-3.424408000
6	2.578734000	-0.998187000	1.164614000
6	3.139122000	-2.188414000	0.708450000
6	4.359155000	-2.665790000	1.165503000
6	5.045215000	-1.961111000	2.143262000
6	4.500908000	-0.789057000	2.646879000
6	3.287625000	-0.326259000	2.155489000
6	2.105248000	1.953892000	-0.491253000
6	1.975534000	-1.222532000	-2.874118000
6	2.781793000	-2.096111000	-3.595352000
6	4.132392000	-2.184022000	-3.307177000
6	4.656927000	-1.371685000	-2.311917000
6	3.817467000	-0.505918000	-1.627720000
6	2.442606000	-0.409244000	-1.847905000
6	2.908456000	2.646024000	-1.397938000
6	3.401105000	3.925339000	-1.174438000
6	3.088486000	4.576948000	0.008325000
6	2.282095000	3.941038000	0.938684000
6	1.787336000	2.670697000	0.663163000
6	-2.133884000	-3.511525000	-0.682349000
6	-2.175453000	-4.850362000	-0.280591000
1	-1.691782000	-5.603507000	-0.894584000
6	-2.826832000	-5.238570000	0.881236000
6	-1.423606000	-3.162556000	-1.978554000
1	-1.243831000	-2.082241000	-1.994414000
1	-1.824215000	-3.195412000	-4.115807000
6	-0.053864000	-3.838433000	-2.093781000
1	0.602834000	-3.533815000	-1.274390000
1	-0.137671000	-4.929431000	-2.081023000
1	0.419219000	-3.561451000	-3.038411000
1	0.371359000	3.639551000	-1.502702000
1	-0.997573000	1.167617000	4.955797000
1	-2.852604000	-6.285300000	1.168942000
1	0.338156000	-1.130791000	5.688719000
6	-0.094697000	-3.010473000	4.728385000

1	-0.142900000	-3.568259000	5.658721000

4

Total Gibbs energy= -4378.346767 a.u.

13	-1.843914000	0.168212000	-0.285200000
7	-3.344382000	-0.770366000	-0.899012000
7	-2.827290000	1.809791000	0.059945000
6	-0.144264000	1.187759000	-2.718584000
6	1.050794000	1.604812000	-3.330435000
6	1.064085000	2.177421000	-4.594810000
1	2.007030000	2.490600000	-5.033195000
6	-0.126987000	2.354688000	-5.298611000
1	-0.116939000	2.803940000	-6.286843000
6	-1.324809000	1.941323000	-4.723409000
1	-2.260959000	2.061617000	-5.260512000
6	-1.326232000	1.363687000	-3.457722000
1	-2.269732000	1.031918000	-3.024437000
6	-3.884177000	2.099793000	-0.639132000
1	-4.281651000	3.114671000	-0.537827000
6	-4.625411000	1.217035000	-1.487732000
6	-5.717528000	1.787312000	-2.186224000
1	-5.850308000	2.865600000	-2.132606000
6	-6.598959000	1.016869000	-2.902365000
1	-7.426629000	1.467662000	-3.437495000
6	-6.412546000	-0.379456000	-2.907664000
1	-7.103662000	-1.009155000	-3.461075000
6	-5.370129000	-0.972974000	-2.232571000
1	-5.245912000	-2.050345000	-2.257716000
6	-4.410277000	-0.199240000	-1.523290000
6	-3.418361000	-2.168602000	-0.579617000
6	-4.362571000	-3.936740000	0.755772000
1	-5.026960000	-4.268255000	1.549653000
6	-4.274877000	-2.573816000	0.463105000
6	-2.334426000	-2.280676000	-3.638605000
1	-3.083166000	-1.497552000	-3.491766000
1	-2.838637000	-3.163732000	-4.049050000
6	-5.082085000	-1.587054000	1.296681000
1	-4.794862000	-0.570784000	1.001356000
6	-6.587749000	-1.742747000	1.047187000
1	-6.834631000	-1.617167000	-0.010964000
1	-6.932188000	-2.734397000	1.362800000
1	-7.146513000	-0.995263000	1.621058000
6	-4.771250000	-1.735483000	2.792230000
1	-3.714801000	-1.546258000	3.007713000

1	-5.378866000	-1.031267000	3.372302000
1	-5.007065000	-2.742913000	3.151813000
6	-2.278233000	2.818324000	0.935360000
6	-1.525275000	3.870386000	0.393211000
6	-0.947610000	4.780286000	1.284236000
1	-0.350991000	5.600128000	0.894080000
6	-1.115780000	4.648515000	2.652600000
1	-0.647678000	5.357497000	3.328522000
6	-1.894987000	3.614422000	3.163040000
1	-2.036915000	3.539005000	4.235486000
6	-2.495348000	2.678834000	2.321826000
6	-1.353362000	4.079081000	-1.101306000
1	-1.708064000	3.188052000	-1.625557000
6	0.112087000	4.265481000	-1.498414000
1	0.197352000	4.332579000	-2.588066000
1	0.537725000	5.177765000	-1.064682000
6	-2.191687000	5.272632000	-1.578630000
1	-3.252350000	5.150059000	-1.334403000
1	-1.849988000	6.199587000	-1.104501000
1	-2.099787000	5.389704000	-2.663352000
6	-3.420412000	1.600123000	2.872373000
1	-3.168698000	0.648830000	2.380263000
6	-3.286512000	1.393421000	4.383248000
1	-3.729372000	2.232608000	4.932451000
1	-3.819035000	0.483400000	4.675604000
6	-4.890424000	1.935482000	2.564236000
1	-5.112012000	1.953137000	1.494149000
1	-5.551737000	1.193095000	3.023537000
1	-5.141974000	2.917158000	2.980947000
5	2.343391000	0.434368000	-0.849004000
9	1.869963000	0.701199000	2.951690000
9	3.975351000	-0.429848000	4.175901000
9	4.954534000	-2.807456000	3.298057000
9	3.778438000	-4.064517000	1.186553000
9	1.641075000	-2.965936000	-0.010235000
9	4.810885000	-0.550360000	0.232096000
9	5.933592000	-2.884560000	-0.379297000
9	4.936018000	-4.394488000	-2.414513000
9	2.757737000	-3.539503000	-3.795436000
9	1.567311000	-1.244173000	-3.136778000
9	5.005276000	1.401499000	-1.577123000
9	6.131462000	3.647696000	-0.687796000
9	4.789743000	5.255043000	1.036537000
9	2.292167000	4.591354000	1.863899000

9	1.151714000	2.357369000	1.020683000
6	-0.752095000	-0.665444000	1.128849000
6	0.514375000	-0.494496000	0.698099000
6	0.812739000	0.218726000	-0.605363000
6	-0.242538000	0.580782000	-1.379720000
6	-1.038674000	-1.460897000	2.341119000
6	-0.945535000	-0.889313000	3.614593000
1	-0.625073000	0.146466000	3.697340000
6	-1.246040000	-1.635444000	4.752425000
6	-1.692570000	-3.555937000	3.371336000
1	-1.980290000	-4.598335000	3.265534000
6	-1.403672000	-2.809559000	2.233378000
1	-1.461208000	-3.268995000	1.249182000
1	2.003567000	1.503432000	-2.811875000
6	1.678377000	-1.096799000	1.427888000
6	2.181583000	-2.333948000	1.036457000
6	3.282126000	-2.916619000	1.645674000
6	3.895802000	-2.267032000	2.708080000
6	3.399019000	-1.047838000	3.147945000
6	2.305347000	-0.479137000	2.506853000
6	3.034020000	1.735106000	-0.301715000
6	2.667107000	-1.588449000	-2.466017000
6	3.263529000	-2.791363000	-2.817466000
6	4.377119000	-3.230970000	-2.115331000
6	4.901363000	-2.448375000	-1.094318000
6	4.307432000	-1.231311000	-0.805117000
6	3.166233000	-0.763492000	-1.457836000
6	4.314142000	2.136590000	-0.705283000
6	4.917018000	3.304681000	-0.267907000
6	4.230709000	4.131382000	0.613651000
6	2.956890000	3.784949000	1.040241000
6	2.382242000	2.606554000	0.583611000
6	-2.597725000	-3.094220000	-1.259037000
6	-2.713296000	-4.443616000	-0.918477000
1	-2.096310000	-5.177804000	-1.424705000
6	-3.598915000	-4.867667000	0.067416000
6	-1.615104000	-2.640486000	-2.332255000
1	-1.113777000	-1.730137000	-1.977703000
1	-1.610368000	-1.921203000	-4.377333000
6	-0.509797000	-3.661155000	-2.609376000
1	0.004006000	-3.957013000	-1.689227000
1	-0.905211000	-4.562727000	-3.090919000
1	0.227624000	-3.226861000	-3.287913000
1	0.705625000	3.409769000	-1.169110000

1	-2.246648000	1.288288000	4.699938000
1	-3.676613000	-5.923687000	0.308617000
1	-1.176079000	-1.172621000	5.733124000
6	-1.627022000	-2.970051000	4.634139000
1	-1.864369000	-3.550859000	5.520183000

TS1

Total Gibbs energy= -4378.31696638 a.u.

5	1.810117000	0.583608000	1.357753000
13	-1.225227000	-0.015400000	-0.577390000
9	1.821478000	-0.333320000	4.262294000
9	-0.110062000	0.118786000	5.992940000
9	-2.324519000	1.535465000	5.279972000
9	-2.552353000	2.492330000	2.751118000
9	-0.615019000	2.010876000	0.948691000
9	4.316572000	2.773298000	-2.349721000
9	3.474036000	5.256373000	-1.642288000
9	1.864407000	5.557577000	0.537905000
9	1.199640000	3.503318000	2.015765000
9	3.506429000	0.640161000	-0.988975000
9	4.822301000	0.998966000	1.256262000
9	6.622089000	-0.884040000	1.613051000
9	5.885206000	-3.411358000	2.322867000
9	3.241093000	-3.975887000	2.705425000
9	1.404479000	-2.100934000	2.336892000
7	-1.515898000	1.403230000	-1.864572000
7	-1.861766000	-1.413802000	-1.686669000
6	0.673671000	-0.332113000	-0.115877000
6	1.375627000	-1.283235000	-0.496670000
6	-3.661452000	0.026845000	1.389565000
6	-2.644085000	0.137982000	0.723807000
6	-2.602910000	1.289377000	-2.573507000
1	-2.883655000	2.155045000	-3.179642000
6	-3.450186000	0.141872000	-2.693110000
6	-3.042616000	-1.185315000	-2.332382000
6	-3.920134000	-2.240508000	-2.708366000
1	-3.637050000	-3.260275000	-2.472342000
6	-5.119950000	-1.990454000	-3.337122000
1	-5.765685000	-2.829438000	-3.581546000
6	-5.524640000	-0.684571000	-3.670054000
1	-6.470077000	-0.506314000	-4.168813000
6	-4.679322000	0.352084000	-3.362490000
1	-4.944315000	1.372069000	-3.632121000
6	-0.751433000	2.617225000	-2.061921000

6	-1.141497000	3.800183000	-1.413292000
6	-0.435861000	4.966537000	-1.726740000
1	-0.713969000	5.899409000	-1.246456000
6	0.615020000	4.953810000	-2.631867000
1	1.155112000	5.869475000	-2.851072000
6	0.999290000	3.760890000	-3.234784000
1	1.843930000	3.759496000	-3.917098000
6	0.325844000	2.568394000	-2.966013000
6	-1.384667000	-2.769437000	-1.684049000
6	-1.721441000	-3.647689000	-0.631932000
6	-1.256309000	-4.963579000	-0.684312000
1	-1.508975000	-5.646898000	0.122185000
6	-0.489902000	-5.414782000	-1.752219000
1	-0.138212000	-6.442119000	-1.777571000
6	-0.188841000	-4.548261000	-2.794182000
1	0.391381000	-4.908507000	-3.639801000
6	-0.633920000	-3.224390000	-2.785986000
6	1.836159000	-3.636192000	-0.149511000
1	0.892061000	-3.702687000	0.381047000
6	2.677920000	-4.734090000	-0.251018000
1	2.387541000	-5.676925000	0.200897000
6	3.902029000	-4.612169000	-0.906274000
1	4.570955000	-5.465499000	-0.963742000
6	4.274633000	-3.400924000	-1.491271000
1	5.227521000	-3.314997000	-2.003214000
6	3.432995000	-2.300042000	-1.415005000
1	3.712737000	-1.355130000	-1.865324000
6	2.212047000	-2.412076000	-0.727862000
6	-4.855033000	-0.209860000	2.147035000
6	-5.059243000	0.387069000	3.398697000
1	-4.310806000	1.064152000	3.795580000
6	-6.212340000	0.105376000	4.122354000
1	-6.362124000	0.568144000	5.092925000
6	-7.170608000	-0.766623000	3.609882000
1	-8.068005000	-0.984967000	4.180506000
6	-6.975198000	-1.359690000	2.363568000
1	-7.720092000	-2.038798000	1.960222000
6	-5.826165000	-1.083750000	1.633181000
1	-5.663861000	-1.535092000	0.657907000
6	0.678575000	0.777729000	2.485317000
6	-0.469460000	1.508796000	2.195462000
6	-1.477000000	1.787290000	3.098422000
6	-1.362421000	1.299209000	4.391250000
6	-0.240245000	0.568622000	4.746703000

6	0.755845000	0.330527000	3.805309000
6	3.076867000	1.839869000	-0.562706000
6	3.529961000	2.930419000	-1.283143000
6	3.117076000	4.201032000	-0.916533000
6	2.300328000	4.348252000	0.188101000
6	1.920059000	3.228171000	0.919284000
6	2.258322000	1.920623000	0.564908000
6	2.991905000	-0.457440000	1.722586000
6	4.357317000	-0.212036000	1.585871000
6	5.334384000	-1.184189000	1.779917000
6	4.966205000	-2.466034000	2.152386000
6	3.623214000	-2.747165000	2.356808000
6	2.686252000	-1.748098000	2.155495000
6	-2.312007000	3.872018000	-0.442757000
1	-2.533747000	2.864289000	-0.076858000
6	-3.567143000	4.411496000	-1.143386000
1	-3.893536000	3.763654000	-1.963603000
1	-3.377761000	5.407492000	-1.559510000
1	-4.393590000	4.490154000	-0.429994000
6	-1.984658000	4.745732000	0.774880000
1	-1.028218000	4.465614000	1.225505000
1	-2.767781000	4.637176000	1.529039000
1	-1.935631000	5.805639000	0.503611000
6	0.675486000	1.286323000	-3.710835000
1	0.446281000	0.436339000	-3.054292000
6	-0.188638000	1.165493000	-4.975763000
1	-1.258733000	1.119642000	-4.748747000
1	0.071737000	0.259246000	-5.533648000
1	-0.018109000	2.028623000	-5.628949000
6	2.153573000	1.182208000	-4.089468000
1	2.804416000	1.240669000	-3.216099000
1	2.451491000	1.965603000	-4.793838000
1	2.336902000	0.223763000	-4.582002000
6	-2.579994000	-3.195105000	0.533990000
1	-3.084900000	-2.273646000	0.235855000
6	-1.706666000	-2.874576000	1.753518000
1	-0.926813000	-2.141534000	1.517932000
1	-1.208306000	-3.782462000	2.117660000
1	-2.316519000	-2.462462000	2.564561000
6	-3.668835000	-4.207387000	0.902985000
1	-4.274839000	-4.475009000	0.030736000
1	-4.330609000	-3.773195000	1.659741000
1	-3.251819000	-5.129314000	1.322296000
6	-0.361263000	-2.343689000	-3.993487000

1	-0.699951000	-1.330650000	-3.753910000
6	1.131264000	-2.278463000	-4.326494000
1	1.697357000	-1.821782000	-3.508359000
1	1.294468000	-1.687817000	-5.234744000
1	1.549484000	-3.274726000	-4.505778000
6	-1.168552000	-2.830101000	-5.204372000
1	-2.237931000	-2.868055000	-4.975459000
1	-0.849188000	-3.833636000	-5.508321000
1	-1.022952000	-2.156633000	-6.056284000

TS2

Total Gibbs energy= -4378.295054 a.u.

5	2.113144000	-0.243893000	-0.404859000
13	-1.648587000	-0.030731000	0.278167000
9	2.060595000	-1.067203000	-3.320334000
9	3.079356000	-3.291678000	-4.366786000
9	3.664378000	-5.403610000	-2.776348000
9	3.270123000	-5.254203000	-0.095289000
9	2.317302000	-3.001880000	0.987322000
9	2.531526000	1.726587000	4.136962000
9	4.836865000	0.387017000	4.645685000
9	5.882287000	-1.269393000	2.753903000
9	4.665035000	-1.562463000	0.401565000
9	1.200613000	1.307620000	1.847993000
9	4.617304000	1.302088000	-0.486804000
9	5.003934000	3.537140000	-1.857855000
9	3.128707000	4.420877000	-3.622936000
9	0.818012000	3.023461000	-3.945459000
9	0.380320000	0.804176000	-2.559199000
7	-2.169498000	-0.250047000	2.139380000
7	-3.366438000	-0.289295000	-0.424877000
6	-0.376734000	2.777580000	-0.250121000
6	-0.955870000	1.726461000	-0.028831000
6	0.796366000	-0.976435000	-0.456858000
6	-0.379724000	-1.512483000	-0.272930000
6	-3.330266000	-0.742277000	2.470184000
1	-3.538418000	-0.816803000	3.541662000
6	-4.370839000	-1.193294000	1.601148000
6	-4.401343000	-0.913273000	0.196772000
6	-5.587405000	-1.275762000	-0.499412000
1	-5.658397000	-1.036441000	-1.554463000
6	-6.620521000	-1.927022000	0.134561000
1	-7.496605000	-2.205925000	-0.444171000
6	-6.570924000	-2.235179000	1.508958000

1	-7.395911000	-2.745004000	1.992651000
6	-5.463462000	-1.848897000	2.220595000
1	-5.405169000	-2.038666000	3.290247000
6	-1.345767000	0.213216000	3.240677000
6	-0.445237000	-0.680490000	3.847218000
6	0.255725000	-0.224096000	4.964135000
1	0.960422000	-0.884493000	5.458182000
6	0.079723000	1.066681000	5.447327000
1	0.640095000	1.398480000	6.315818000
6	-0.773068000	1.942712000	4.793661000
1	-0.875715000	2.960368000	5.156741000
6	-1.494898000	1.541272000	3.667355000
6	-3.646499000	0.388542000	-1.665891000
6	-3.361380000	-0.217238000	-2.901351000
6	-3.621401000	0.506644000	-4.069014000
1	-3.408135000	0.051304000	-5.032769000
6	-4.157549000	1.784479000	-4.019994000
1	-4.355791000	2.328371000	-4.938616000
6	-4.448121000	2.363190000	-2.789928000
1	-4.880270000	3.359859000	-2.755707000
6	-4.198270000	1.686154000	-1.596932000
6	1.542191000	4.253535000	0.158008000
1	1.824876000	3.613251000	0.988014000
6	2.328661000	5.339901000	-0.211626000
1	3.240152000	5.556617000	0.337164000
6	1.959359000	6.136215000	-1.293770000
1	2.584404000	6.972327000	-1.591132000
6	0.792087000	5.852191000	-2.002496000
1	0.509234000	6.465924000	-2.851927000
6	-0.001590000	4.771915000	-1.638547000
1	-0.895606000	4.521532000	-2.201356000
6	0.374253000	3.958074000	-0.559394000
6	-0.866465000	-2.900170000	-0.457394000
6	-0.399823000	-3.759973000	-1.459831000
1	0.348016000	-3.412171000	-2.162041000
6	-0.929446000	-5.038297000	-1.607859000
1	-0.559906000	-5.680546000	-2.402012000
6	-1.934226000	-5.489786000	-0.755407000
1	-2.348273000	-6.485710000	-0.877015000
6	-2.410151000	-4.647487000	0.245476000
1	-3.200142000	-4.976497000	0.913970000
6	-1.885092000	-3.368120000	0.384530000
1	-2.272110000	-2.720513000	1.164306000
6	2.216326000	-1.901999000	-1.099787000

6	2.513160000	-3.028947000	-0.326659000
6	3.001327000	-4.207038000	-0.866666000
6	3.199023000	-4.287684000	-2.238965000
6	2.898128000	-3.201269000	-3.053180000
6	2.388269000	-2.044005000	-2.483866000
6	2.350317000	0.644665000	2.035163000
6	3.009586000	0.866493000	3.239343000
6	4.196866000	0.198728000	3.495115000
6	4.729199000	-0.642413000	2.527498000
6	4.063779000	-0.793660000	1.320491000
6	2.853061000	-0.172818000	1.029089000
6	2.454719000	0.952965000	-1.413436000
6	3.635429000	1.688265000	-1.312525000
6	3.873418000	2.853345000	-2.031534000
6	2.920638000	3.307002000	-2.928951000
6	1.747197000	2.586560000	-3.096582000
6	1.535912000	1.440017000	-2.345999000
6	-0.241358000	-2.097728000	3.330492000
1	-0.335511000	-2.070436000	2.238464000
6	-1.299375000	-3.065206000	3.881545000
1	-2.320398000	-2.779451000	3.608473000
1	-1.243604000	-3.100991000	4.975375000
1	-1.124478000	-4.074445000	3.493752000
6	1.151785000	-2.650633000	3.645293000
1	1.942697000	-1.966986000	3.318757000
1	1.292354000	-3.603376000	3.128725000
1	1.275607000	-2.838767000	4.717205000
6	-2.412396000	2.522301000	2.954483000
1	-2.540154000	2.176446000	1.924434000
6	-3.794658000	2.569291000	3.616205000
1	-4.292261000	1.593023000	3.587304000
1	-4.441338000	3.289772000	3.102759000
1	-3.711284000	2.873571000	4.665763000
6	-1.793576000	3.919689000	2.862321000
1	-0.780446000	3.866631000	2.452370000
1	-1.750682000	4.414812000	3.838461000
1	-2.395430000	4.549841000	2.201011000
6	-2.837162000	-1.638964000	-3.015104000
1	-2.693203000	-2.031711000	-2.004316000
6	-1.493681000	-1.686919000	-3.751952000
1	-0.710215000	-1.158932000	-3.200091000
1	-1.572955000	-1.224422000	-4.741893000
1	-1.181213000	-2.726534000	-3.896127000
6	-3.855113000	-2.549541000	-3.714956000

1	-4.822457000	-2.538356000	-3.203618000
1	-3.487964000	-3.581336000	-3.722389000
1	-4.016976000	-2.239982000	-4.753715000
6	-4.573917000	2.339052000	-0.275039000
1	-4.142308000	1.742729000	0.536658000
6	-4.017525000	3.760016000	-0.144843000
1	-2.927626000	3.764590000	-0.240542000
1	-4.283219000	4.173143000	0.835008000
1	-4.438834000	4.429995000	-0.902005000
6	-6.097644000	2.339623000	-0.089190000
1	-6.506713000	1.325911000	-0.138177000
1	-6.579066000	2.938616000	-0.870895000
1	-6.362995000	2.772848000	0.881612000

TS3

Total Gibbs energy= -4378.322353 a.u.

5	1.533304000	0.388772000	0.143968000
13	-1.626369000	-0.453718000	-0.006773000
9	2.342197000	-3.005541000	-0.567482000
9	4.695543000	-3.653315000	-1.697607000
9	5.855152000	-1.975803000	-3.494610000
9	4.639334000	0.364103000	-4.149020000
9	2.287275000	1.024198000	-3.025668000
9	2.099873000	-3.125711000	3.663126000
9	4.784333000	-3.236263000	3.200045000
9	5.897361000	-1.579683000	1.346763000
9	4.385249000	0.160757000	0.014498000
9	0.579260000	-1.403793000	2.337856000
9	3.576720000	2.042389000	1.581725000
9	3.978657000	4.622097000	1.197618000
9	2.517782000	5.996407000	-0.635831000
9	0.627684000	4.702515000	-2.108374000
9	0.191085000	2.105476000	-1.728468000
7	-2.595978000	-1.734707000	1.100245000
7	-3.176841000	0.233570000	-0.822429000
6	-0.216509000	1.724977000	1.802019000
6	-0.684786000	0.801628000	1.144325000
6	0.982494000	-0.570690000	-1.021342000
6	-0.239572000	-1.106884000	-1.244539000
6	-3.814438000	-2.101190000	0.807395000
1	-4.258221000	-2.864252000	1.453799000
6	-4.653213000	-1.604285000	-0.233137000
6	-4.363975000	-0.403069000	-0.959212000
6	-5.401756000	0.104818000	-1.790337000

1	-5.233124000	1.042335000	-2.307732000
6	-6.585371000	-0.575331000	-1.954165000
1	-7.338288000	-0.161881000	-2.619527000
6	-6.846189000	-1.785895000	-1.279046000
1	-7.786428000	-2.305893000	-1.420092000
6	-5.893525000	-2.265500000	-0.417122000
1	-6.078094000	-3.172237000	0.155016000
6	-2.064122000	-2.297774000	2.326968000
6	-1.326280000	-3.490042000	2.269822000
6	-0.896595000	-4.042493000	3.475419000
1	-0.317122000	-4.959788000	3.466479000
6	-1.163052000	-3.414176000	4.685845000
1	-0.815520000	-3.857340000	5.614080000
6	-1.836436000	-2.200943000	4.706122000
1	-2.002843000	-1.699701000	5.654392000
6	-2.289795000	-1.606733000	3.525782000
6	-3.054454000	1.587119000	-1.298639000
6	-2.561683000	1.854404000	-2.588889000
6	-2.336343000	3.188165000	-2.942175000
1	-1.937516000	3.415358000	-3.927203000
6	-2.597284000	4.223597000	-2.057007000
1	-2.392259000	5.250167000	-2.345213000
6	-3.145578000	3.944511000	-0.809432000
1	-3.400038000	4.763834000	-0.141515000
6	-3.395358000	2.631200000	-0.411110000
6	1.350627000	2.775088000	3.389920000
1	1.642655000	1.789426000	3.739804000
6	1.987323000	3.916985000	3.859795000
1	2.775243000	3.827889000	4.600510000
6	1.627059000	5.171216000	3.368745000
1	2.136719000	6.059368000	3.729021000
6	0.621766000	5.288075000	2.409488000
1	0.348326000	6.265342000	2.023483000
6	-0.012215000	4.151104000	1.923566000
1	-0.776081000	4.219060000	1.152446000
6	0.355279000	2.886663000	2.409468000
6	-0.577323000	-2.069082000	-2.333173000
6	0.130742000	-2.226926000	-3.537184000
1	1.004114000	-1.620020000	-3.739508000
6	-0.294219000	-3.117465000	-4.518368000
1	0.275824000	-3.203836000	-5.438910000
6	-1.444329000	-3.881069000	-4.337968000
1	-1.772464000	-4.572332000	-5.108031000
6	-2.177872000	-3.728344000	-3.165975000

1	-3.091664000	-4.293819000	-3.006869000
6	-1.750553000	-2.831593000	-2.193788000
1	-2.353842000	-2.723219000	-1.296221000
6	2.225293000	-0.974825000	-1.769703000
6	2.860179000	-0.131512000	-2.676762000
6	4.070967000	-0.457964000	-3.271212000
6	4.693338000	-1.653031000	-2.938697000
6	4.094458000	-2.513236000	-2.026893000
6	2.875632000	-2.168428000	-1.461782000
6	1.893750000	-1.365078000	2.100511000
6	2.667988000	-2.282811000	2.799846000
6	4.029989000	-2.353003000	2.555502000
6	4.596625000	-1.499341000	1.618437000
6	3.789524000	-0.587787000	0.955047000
6	2.415544000	-0.474753000	1.171004000
6	1.910639000	1.907097000	-0.119575000
6	2.857825000	2.624731000	0.616360000
6	3.084537000	3.984214000	0.443748000
6	2.338543000	4.688596000	-0.487718000
6	1.371891000	4.027445000	-1.232601000
6	1.175350000	2.670097000	-1.029903000
6	-0.952462000	-4.119841000	0.939091000
1	-0.823327000	-3.303448000	0.219225000
6	-2.050433000	-5.052787000	0.412828000
1	-3.001719000	-4.532199000	0.255301000
1	-2.227354000	-5.865586000	1.126159000
1	-1.748485000	-5.490006000	-0.544658000
6	0.382917000	-4.865996000	0.993050000
1	1.154022000	-4.266255000	1.485593000
1	0.719592000	-5.088084000	-0.023406000
1	0.293072000	-5.816385000	1.530933000
6	-2.983993000	-0.253651000	3.556324000
1	-2.848393000	0.211585000	2.574091000
6	-4.489840000	-0.397221000	3.807017000
1	-4.977277000	-0.997881000	3.030881000
1	-4.970895000	0.587724000	3.819581000
1	-4.674843000	-0.880476000	4.773047000
6	-2.347081000	0.692920000	4.577422000
1	-1.265636000	0.759345000	4.421544000
1	-2.531275000	0.368017000	5.606896000
1	-2.770163000	1.695981000	4.472118000
6	-2.330027000	0.760263000	-3.621190000
1	-2.447450000	-0.211500000	-3.129102000
6	-0.929401000	0.813622000	-4.240462000

1	-0.149264000	0.682638000	-3.488938000
1	-0.755385000	1.770564000	-4.745202000
1	-0.828671000	0.019375000	-4.987001000
6	-3.380956000	0.854016000	-4.738829000
1	-4.400364000	0.775781000	-4.351264000
1	-3.231729000	0.045944000	-5.462390000
1	-3.292155000	1.807387000	-5.272277000
6	-4.125874000	2.365736000	0.897794000
1	-4.058930000	1.294689000	1.118098000
6	-3.539706000	3.120057000	2.092253000
1	-2.501491000	2.831098000	2.276393000
1	-4.122186000	2.895902000	2.993296000
1	-3.575642000	4.205436000	1.946824000
6	-5.613919000	2.713546000	0.737716000
1	-6.068160000	2.158374000	-0.087857000
1	-5.736596000	3.784142000	0.536884000
1	-6.162793000	2.474471000	1.655200000

TS4

Total Gibbs energy= -4378.303548 a.u.

13	1.773249000	0.102769000	0.232915000
7	3.247382000	-1.009956000	0.536448000
7	2.841806000	1.667268000	-0.172512000
6	0.943707000	1.047185000	3.109895000
6	0.000004000	1.572062000	4.005783000
6	0.375955000	1.957139000	5.285951000
1	-0.367803000	2.362326000	5.965430000
6	1.700899000	1.822384000	5.701955000
1	1.990931000	2.123487000	6.703795000
6	2.645152000	1.293978000	4.827925000
1	3.677947000	1.174547000	5.141359000
6	2.266422000	0.909494000	3.544407000
1	3.006173000	0.480511000	2.874177000
6	4.022039000	1.810376000	0.356142000
1	4.492246000	2.792734000	0.248723000
6	4.802844000	0.807094000	1.010590000
6	6.041333000	1.229216000	1.552261000
1	6.267845000	2.292818000	1.539635000
6	6.943269000	0.335207000	2.072037000
1	7.884807000	0.671686000	2.490172000
6	6.623446000	-1.035551000	2.027873000
1	7.327630000	-1.761763000	2.423882000
6	5.434781000	-1.485956000	1.498406000
1	5.213307000	-2.547720000	1.483454000

6	4.456488000	-0.582373000	1.000576000
6	3.128468000	-2.392588000	0.158530000
6	3.663995000	-4.170779000	-1.373720000
1	4.164837000	-4.524309000	-2.271628000
6	3.780555000	-2.826896000	-1.012645000
6	2.601620000	-2.695532000	3.362482000
1	3.424168000	-2.006592000	3.148210000
1	3.029769000	-3.680623000	3.583063000
6	4.584765000	-1.892979000	-1.907689000
1	4.493862000	-0.872401000	-1.517856000
6	6.073043000	-2.265338000	-1.909998000
1	6.490496000	-2.261632000	-0.899145000
1	6.222609000	-3.263435000	-2.337372000
1	6.640680000	-1.552683000	-2.518198000
6	4.035212000	-1.885187000	-3.339378000
1	2.994293000	-1.549139000	-3.370956000
1	4.638915000	-1.220525000	-3.968304000
1	4.073793000	-2.884275000	-3.787367000
6	2.297194000	2.778955000	-0.920815000
6	1.696120000	3.847353000	-0.240834000
6	1.135411000	4.868975000	-1.012650000
1	0.651404000	5.705172000	-0.516121000
6	1.169489000	4.824870000	-2.396478000
1	0.708765000	5.619248000	-2.975335000
6	1.793233000	3.763850000	-3.045525000
1	1.827036000	3.752346000	-4.129324000
6	2.371547000	2.718364000	-2.327209000
6	1.659794000	3.954608000	1.274399000
1	2.033344000	3.021909000	1.706648000
6	0.237529000	4.157177000	1.805219000
1	0.255935000	4.238563000	2.896668000
1	-0.215138000	5.071090000	1.404550000
6	2.578246000	5.088660000	1.749930000
1	3.604810000	4.953094000	1.392794000
1	2.224653000	6.056915000	1.378659000
1	2.596034000	5.128935000	2.843638000
6	3.112536000	1.590194000	-3.036419000
1	2.823555000	0.640269000	-2.561347000
6	2.777826000	1.488311000	-4.527246000
1	3.252770000	2.301289000	-5.088439000
1	3.156166000	0.541615000	-4.924484000
6	4.636538000	1.752120000	-2.900679000
1	4.987914000	1.680849000	-1.868317000
1	5.149384000	0.972346000	-3.474200000

1	4.945133000	2.724836000	-3.299407000
5	-2.076790000	0.482890000	0.968499000
9	-2.286518000	1.069651000	-2.595066000
9	-4.353641000	0.039008000	-3.960452000
9	-5.251343000	-2.467045000	-3.404698000
9	-4.027965000	-3.946417000	-1.471190000
9	-1.935238000	-2.939829000	-0.125704000
9	-4.694263000	-0.419174000	-0.018080000
9	-5.991401000	-2.591691000	0.798096000
9	-5.016902000	-4.082474000	2.862705000
9	-2.700239000	-3.343077000	4.084681000
9	-1.378558000	-1.189632000	3.278980000
9	-4.708934000	1.522680000	1.728166000
9	-5.773359000	3.819767000	0.940062000
9	-4.403920000	5.476356000	-0.730303000
9	-1.931610000	4.764379000	-1.608848000
9	-0.852453000	2.455264000	-0.866039000
6	0.324913000	-0.529703000	-0.941276000
6	-0.924514000	-0.340303000	-0.496890000
6	-0.656136000	0.535375000	1.351186000
6	0.576108000	0.614108000	1.740048000
6	0.487926000	-1.249689000	-2.237994000
6	0.396243000	-0.542870000	-3.441066000
1	0.203563000	0.527480000	-3.401678000
6	0.519316000	-1.204121000	-4.660916000
6	0.803829000	-3.293073000	-3.496599000
1	0.962603000	-4.367724000	-3.507514000
6	0.688186000	-2.633800000	-2.275929000
1	0.738647000	-3.193232000	-1.344730000
1	-1.032973000	1.680160000	3.684717000
6	-2.073426000	-0.881430000	-1.275507000
6	-2.518081000	-2.183953000	-1.061513000
6	-3.592537000	-2.720824000	-1.753110000
6	-4.223584000	-1.964089000	-2.731100000
6	-3.770578000	-0.681186000	-3.005321000
6	-2.706526000	-0.159397000	-2.284028000
6	-2.748097000	1.830571000	0.422882000
6	-2.508673000	-1.492261000	2.627627000
6	-3.183581000	-2.621901000	3.073591000
6	-4.365088000	-3.000497000	2.454916000
6	-4.866045000	-2.232941000	1.411570000
6	-4.170352000	-1.104877000	1.006483000
6	-2.955730000	-0.709698000	1.564600000
6	-4.000519000	2.258837000	0.864792000

6	-4.574402000	3.464540000	0.481962000
6	-3.877083000	4.313118000	-0.363943000
6	-2.618030000	3.942888000	-0.813418000
6	-2.080280000	2.728354000	-0.413206000
6	2.330641000	-3.267820000	0.925578000
6	2.235321000	-4.598474000	0.511939000
1	1.626451000	-5.292077000	1.081492000
6	2.903301000	-5.053097000	-0.620191000
6	1.619458000	-2.796147000	2.187759000
1	1.223928000	-1.789104000	2.010344000
1	2.084154000	-2.334214000	4.257332000
6	0.422011000	-3.666584000	2.572946000
1	-0.278361000	-3.774618000	1.737602000
1	0.734569000	-4.665497000	2.896453000
1	-0.111324000	-3.205675000	3.407109000
1	-0.401088000	3.309951000	1.539120000
1	1.702767000	1.525807000	-4.717610000
1	2.819889000	-6.094040000	-0.918113000
1	0.447116000	-0.642195000	-5.588171000
6	0.725758000	-2.581895000	-4.692155000
1	0.821009000	-3.097357000	-5.642908000

IV. Collected ^{1}H, ^{13}C, ^{19}F, ^{29}Si and ^{11}B NMR spectra.

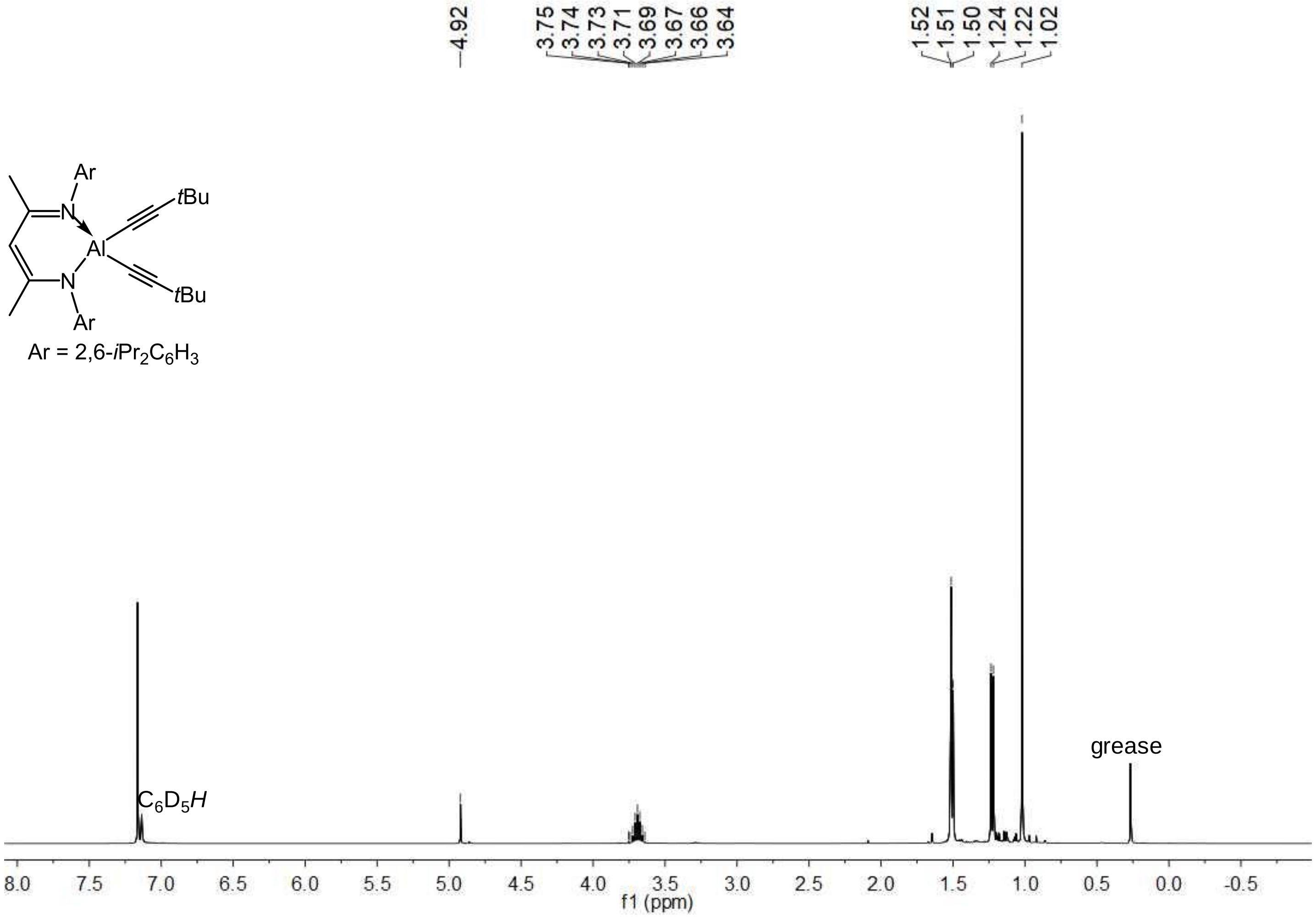

Figure S6-1. ^{1}H NMR spectrum of **1a** in C_6D_6

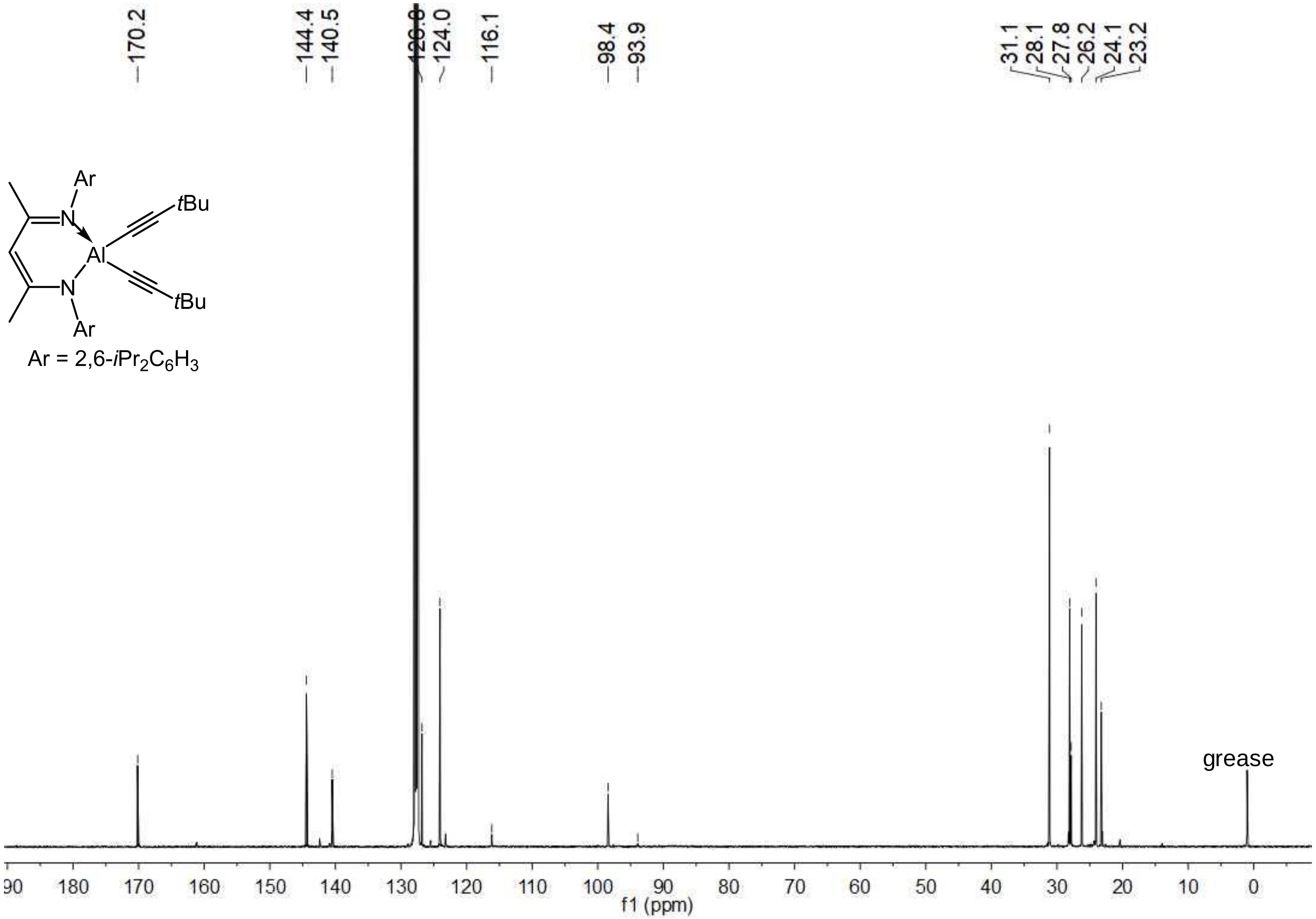

Figure S6-2. ^{13}C NMR spectrum of **1a** in C_6D_6

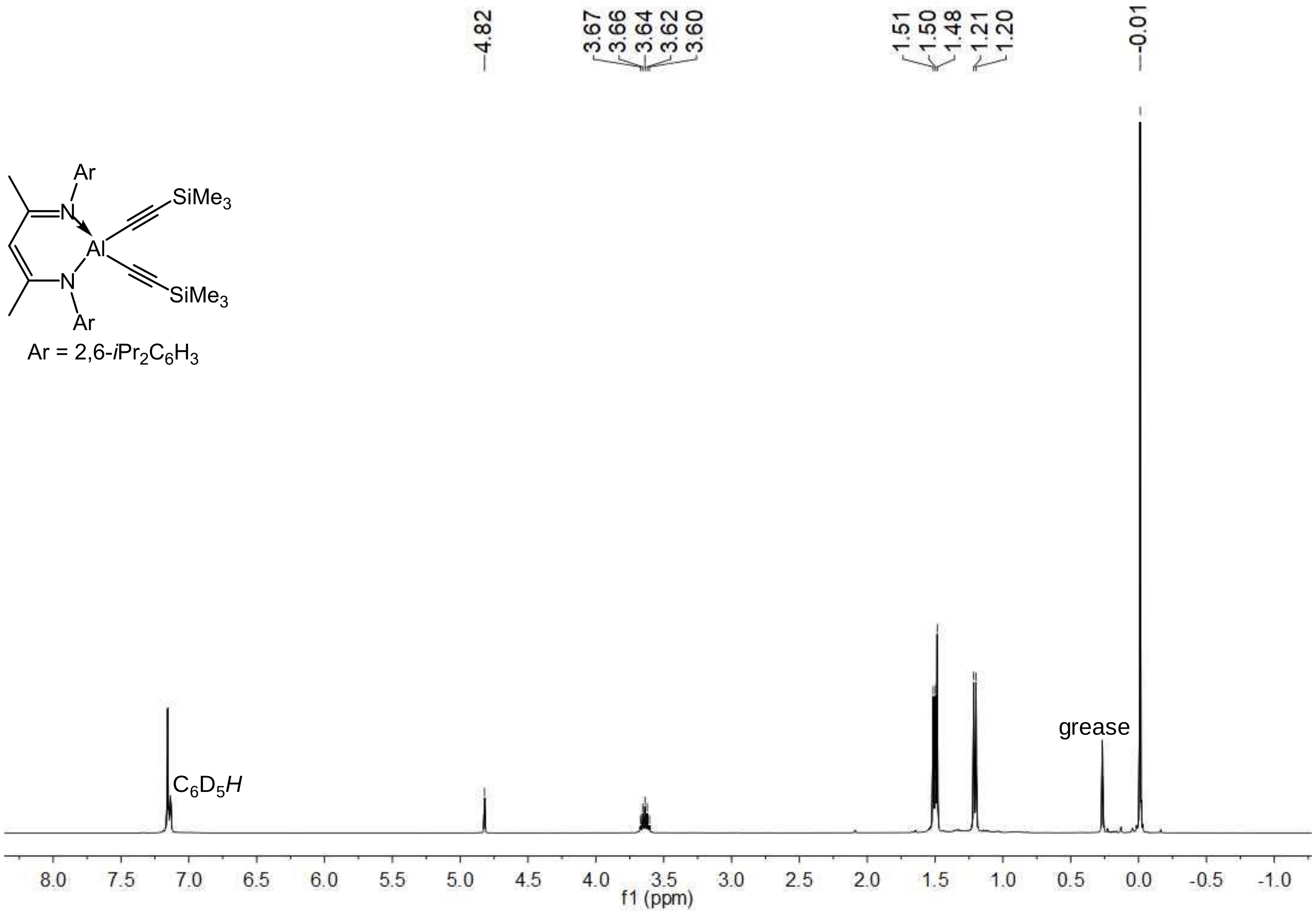

Figure S6-3. ^{1}H NMR spectrum of **1b** in C_6D_6

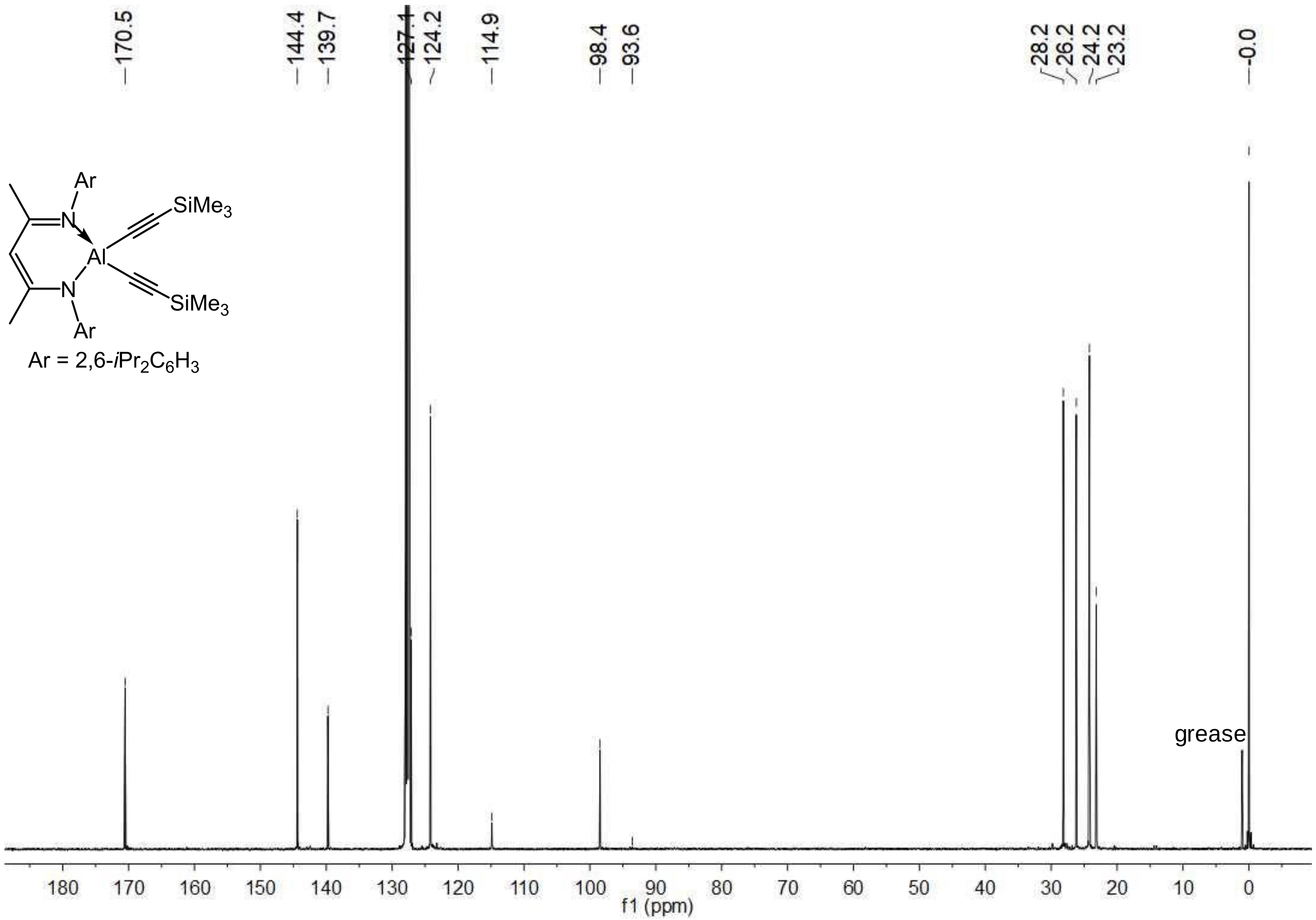

Figure S6-4. ^{13}C NMR spectrum of **1b** in C_6D_6

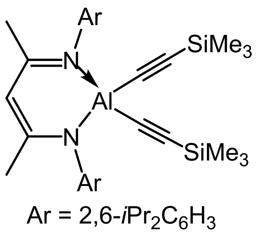

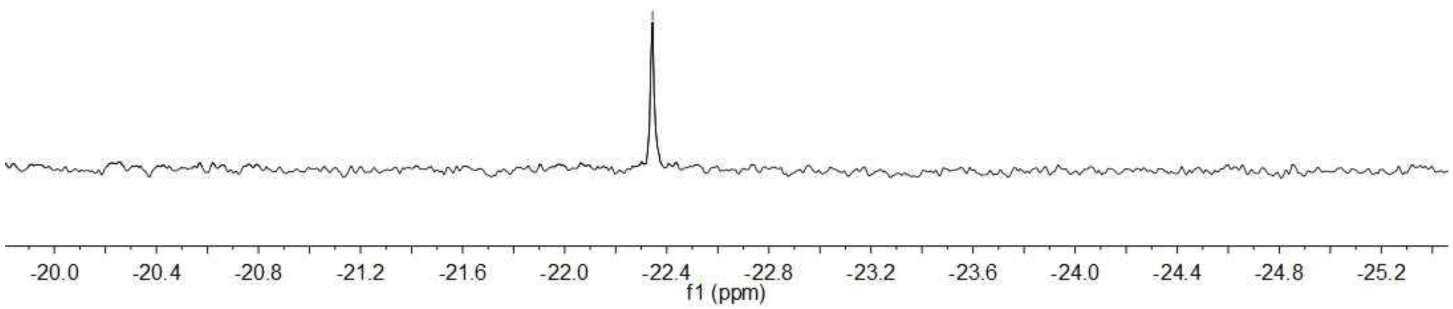

Figure S6-5. ^{29}Si NMR spectrum of **1b** in C_6D_6

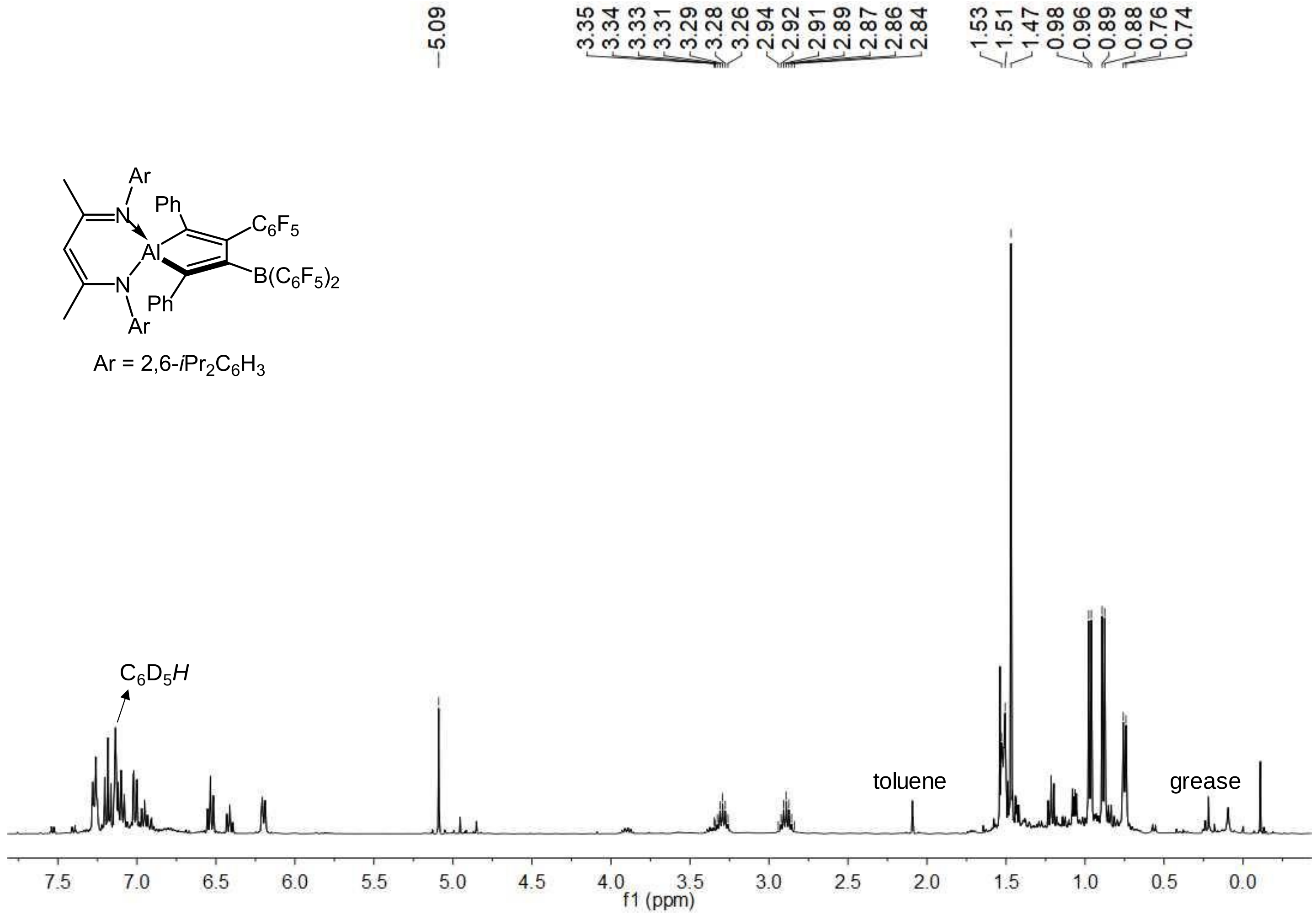

Figure S6-6. ^{1}H NMR spectrum of **2** in C_6D_6

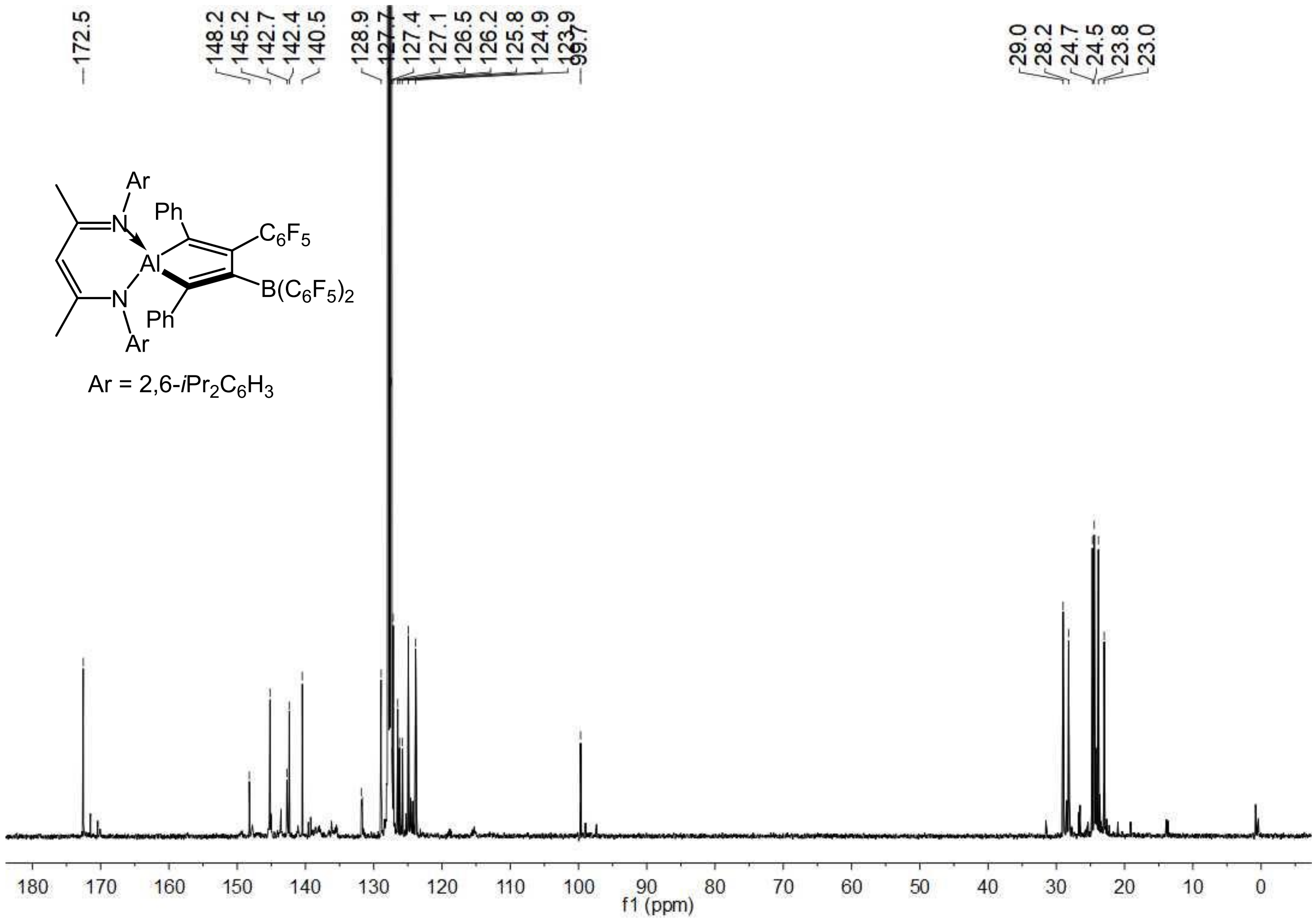

Figure S6-7. ^{13}C NMR spectrum of **2** in C_6D_6

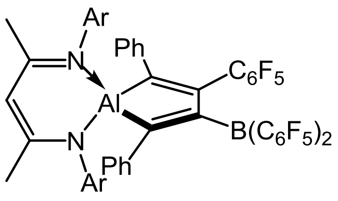

Ar = 2,6-iPr$_2$C$_6$H$_3$

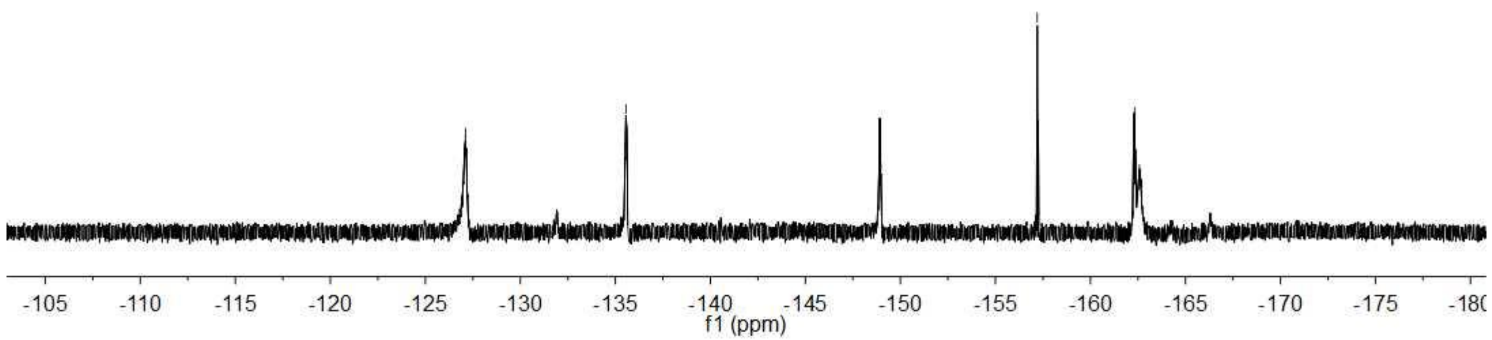

Figure S6-8. ^{19}F NMR spectrum of **2** in C_6D_6.

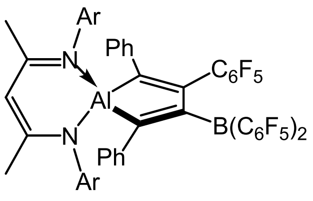

Ar = 2,6-iPr$_2$C$_6$H$_3$

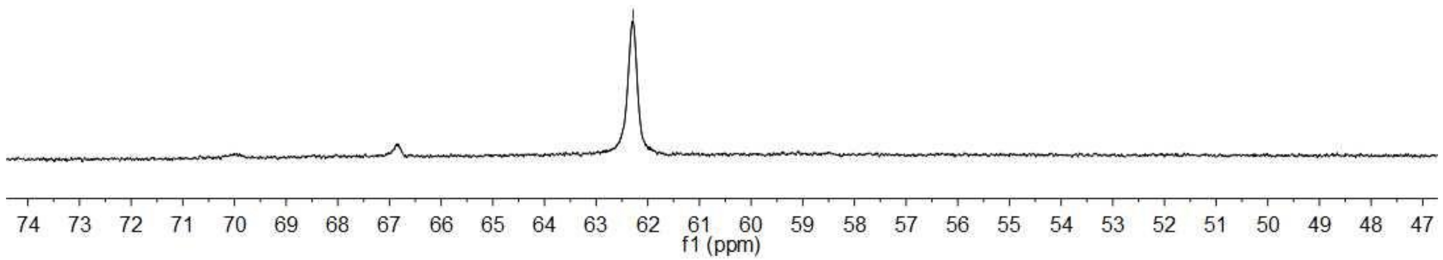

Figure S6-9. ^{11}B NMR spectrum of **2** in C_6D_6.

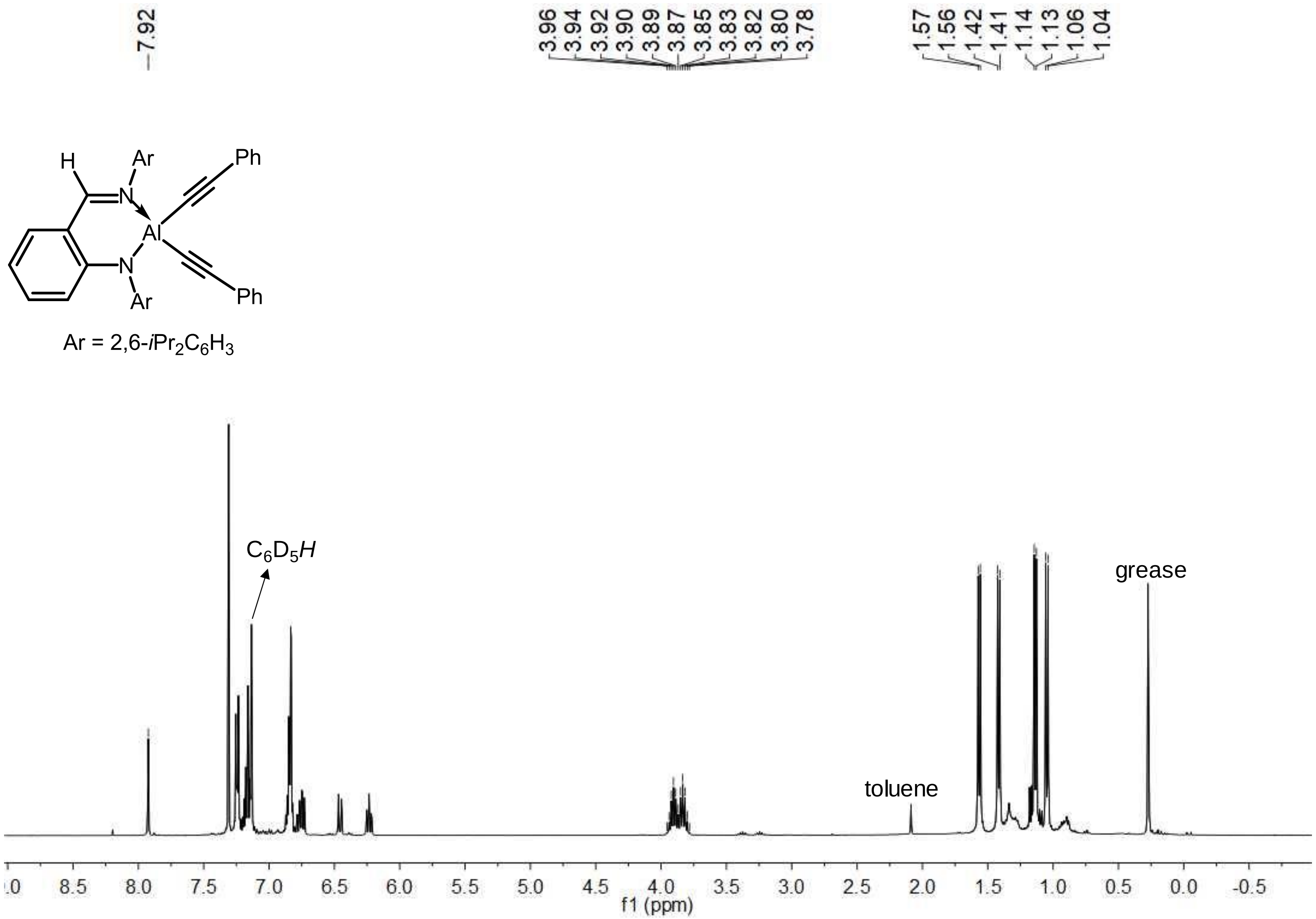

Figure S6-10. ^{1}H NMR spectrum of **3** in C_6D_6

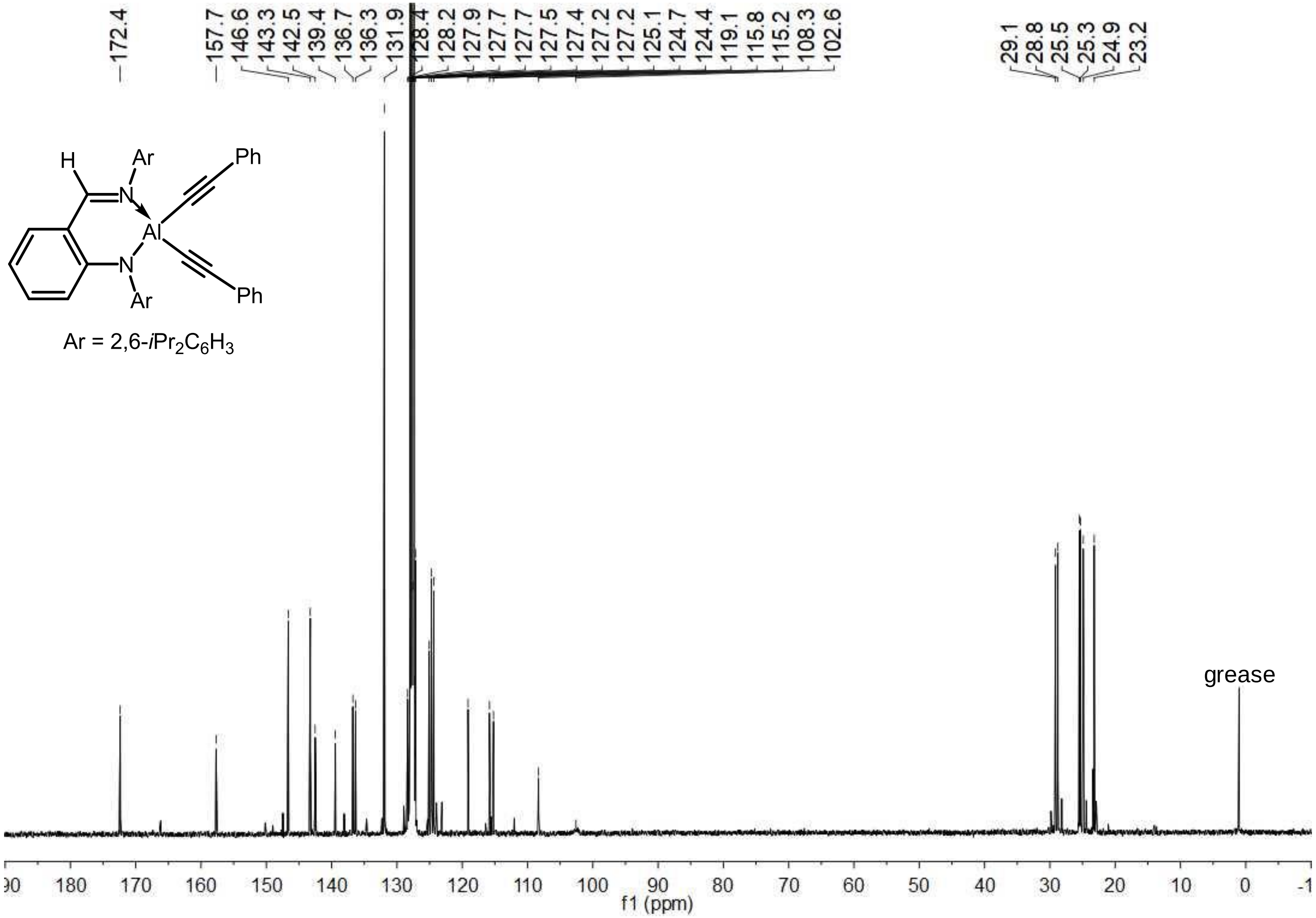

Figure S6-11. ^{13}C NMR spectrum of **3** in C_6D_6

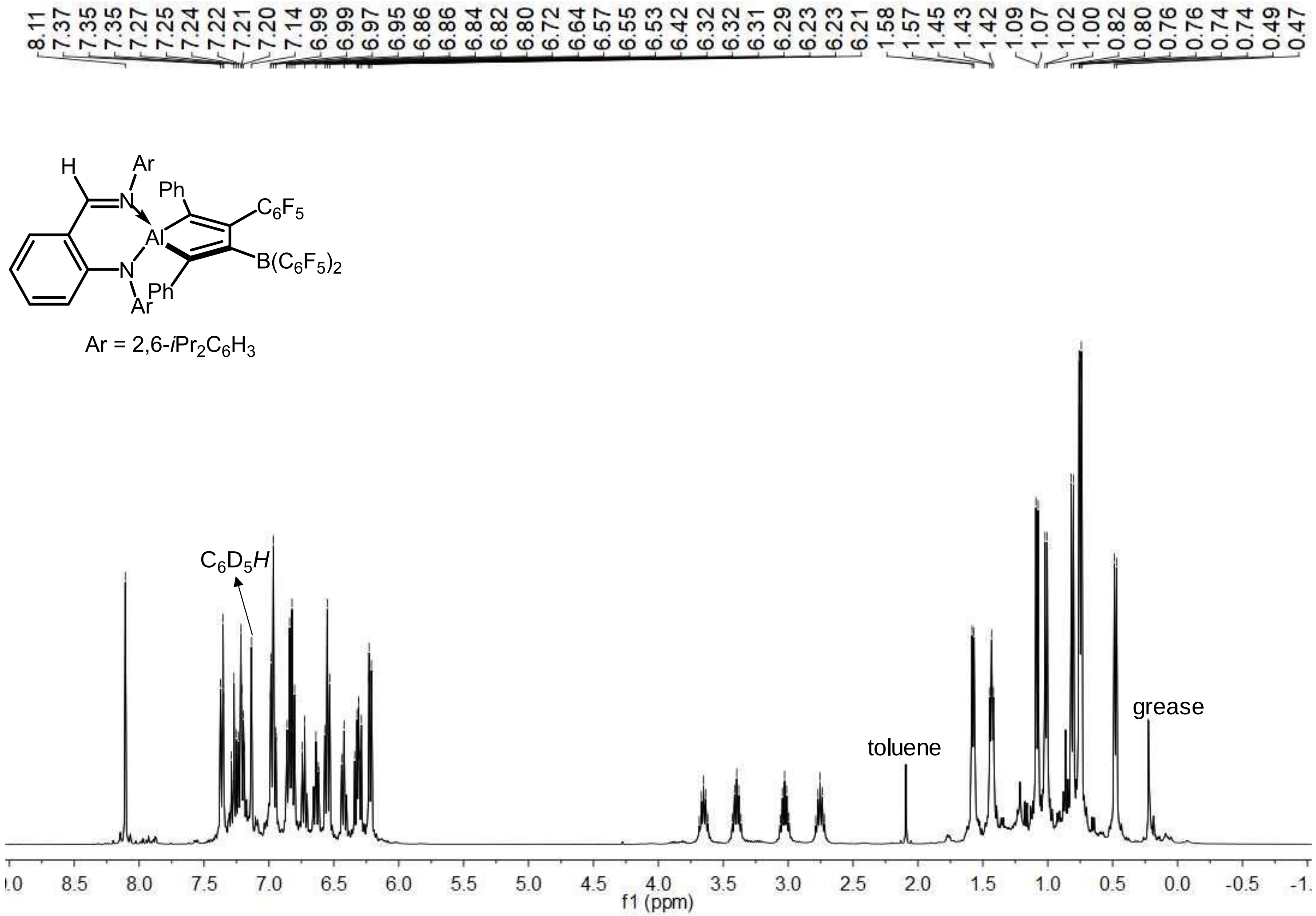

Figure S6-12. ^{1}H NMR spectrum of **4** in C_6D_6

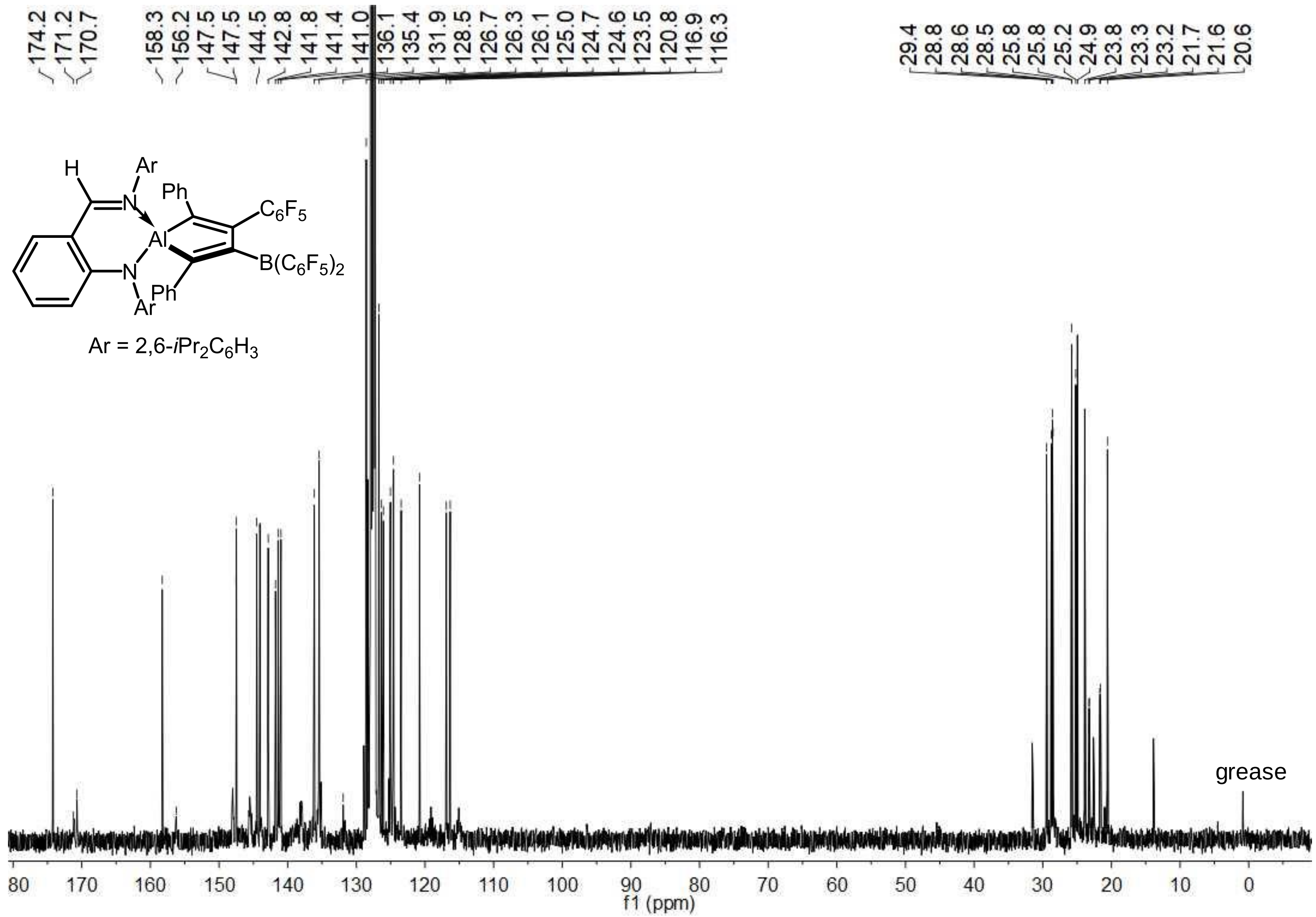

Figure S6-13. ^{13}C NMR spectrum of **4** in C_6D_6

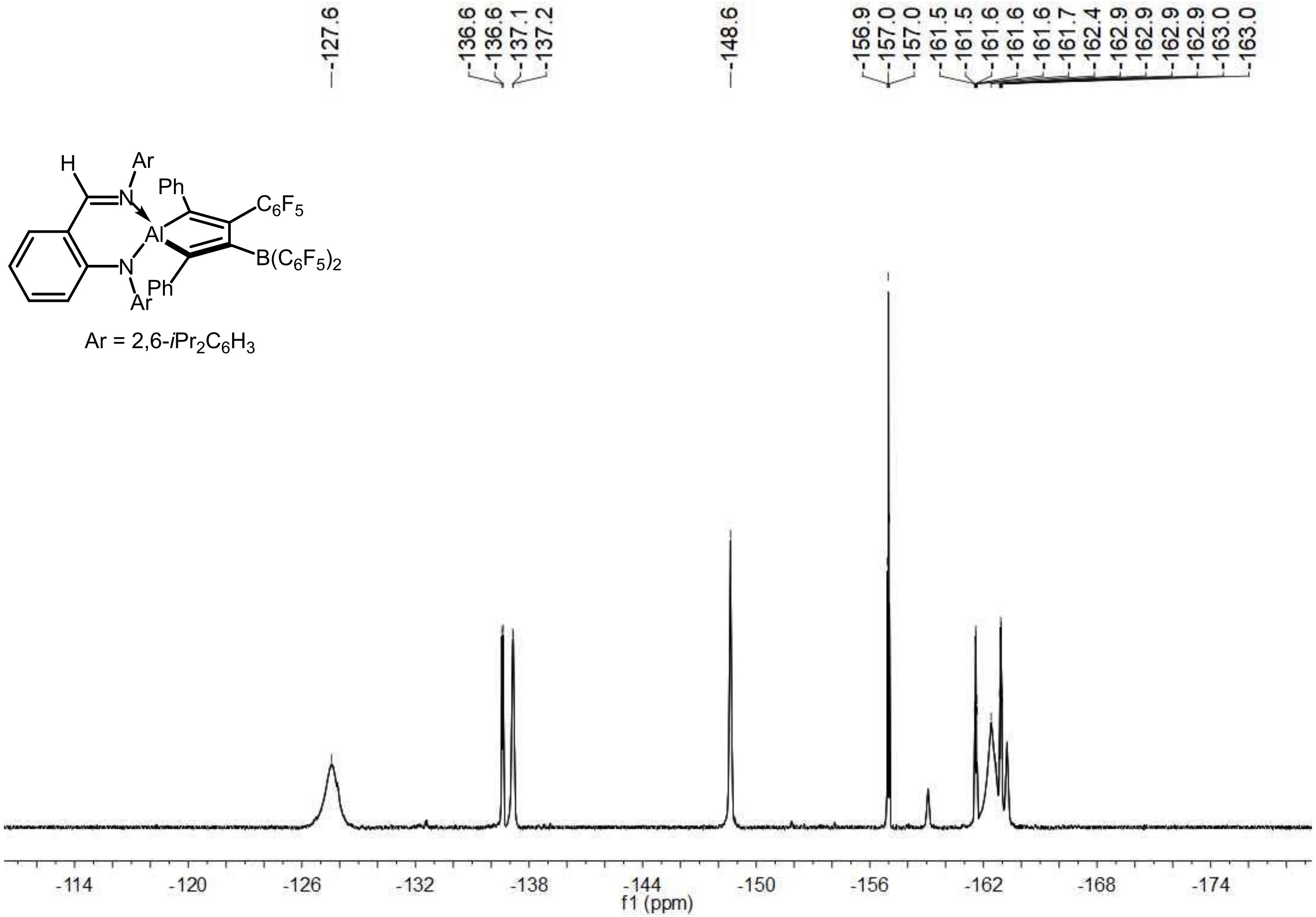

Figure S6-14. ^{19}F NMR spectrum of **4** in C_6D_6

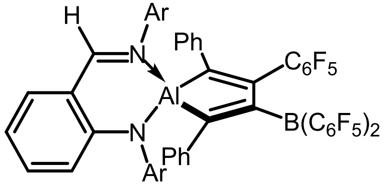

Ar = 2,6-*i*$Pr_2C_6H_3$

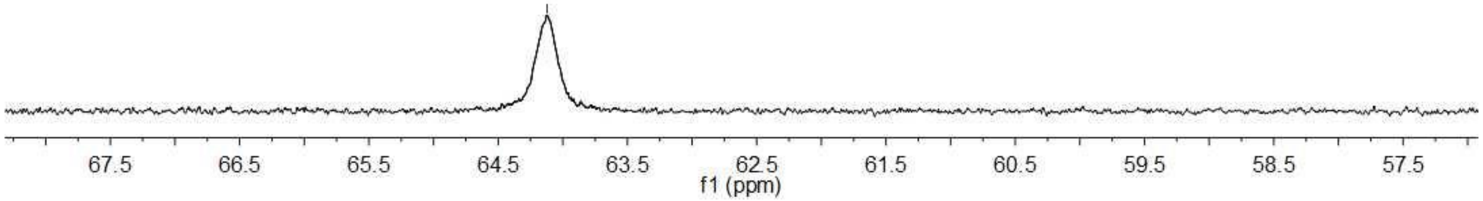

Figure S6-15. ^{11}B NMR spectrum of **4** in C_6D_6.

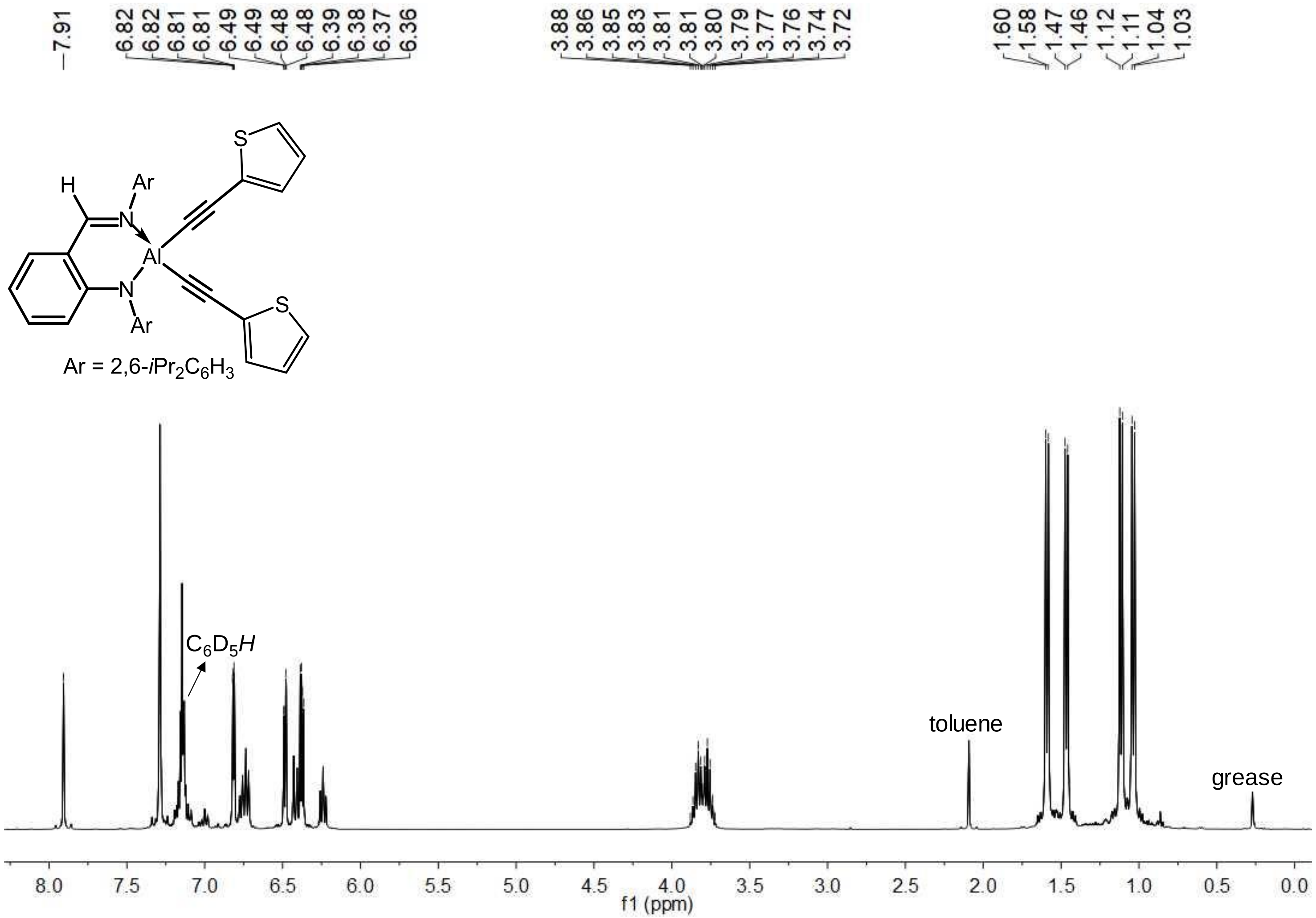

Figure S6-16. ^{1}H NMR spectrum of **5** in C_6D_6

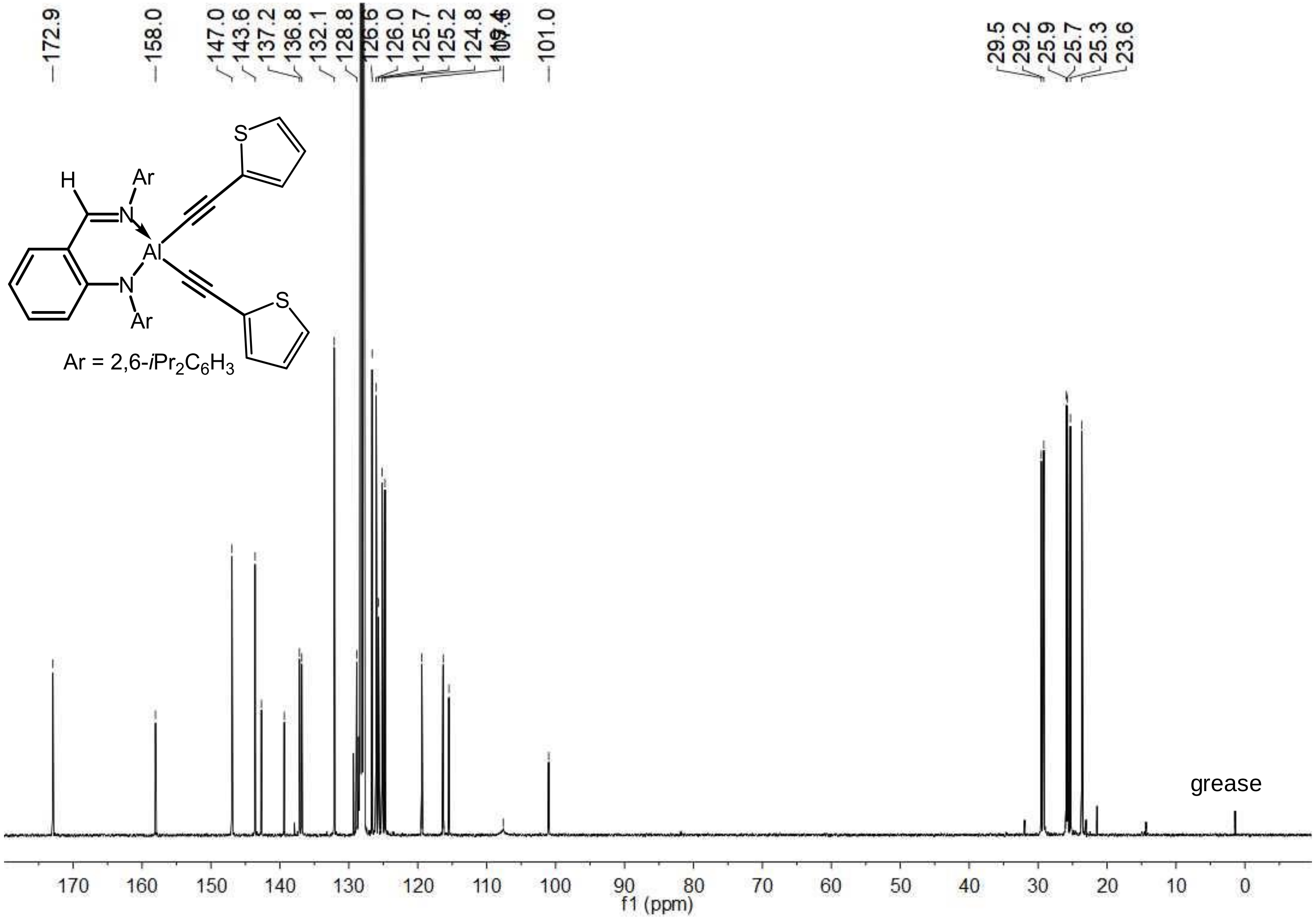

Figure S6-17. ^{13}C NMR spectrum of **5** in C_6D_6

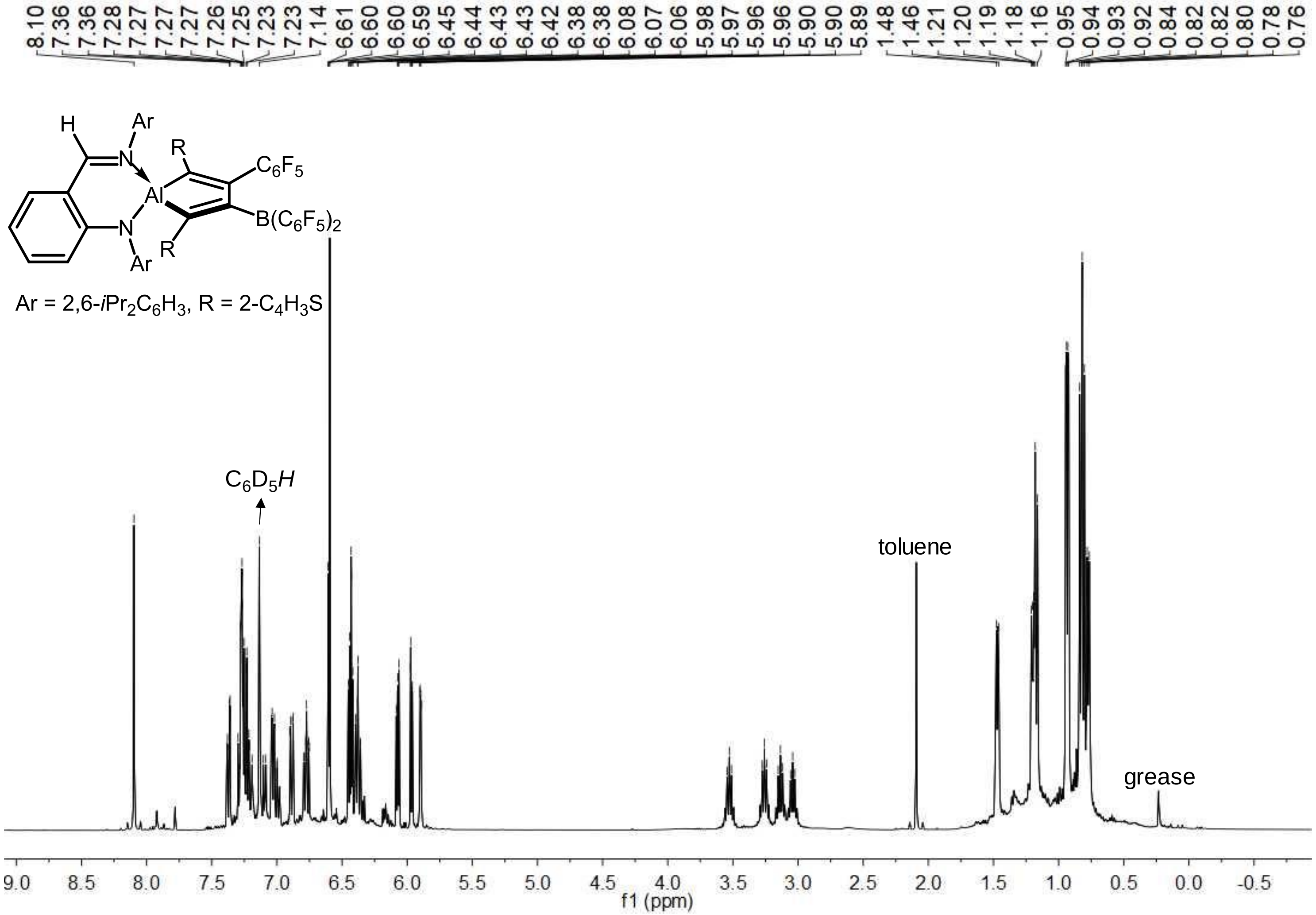

Figure S6-18. ^{1}H NMR spectrum of **6** in C_6D_6

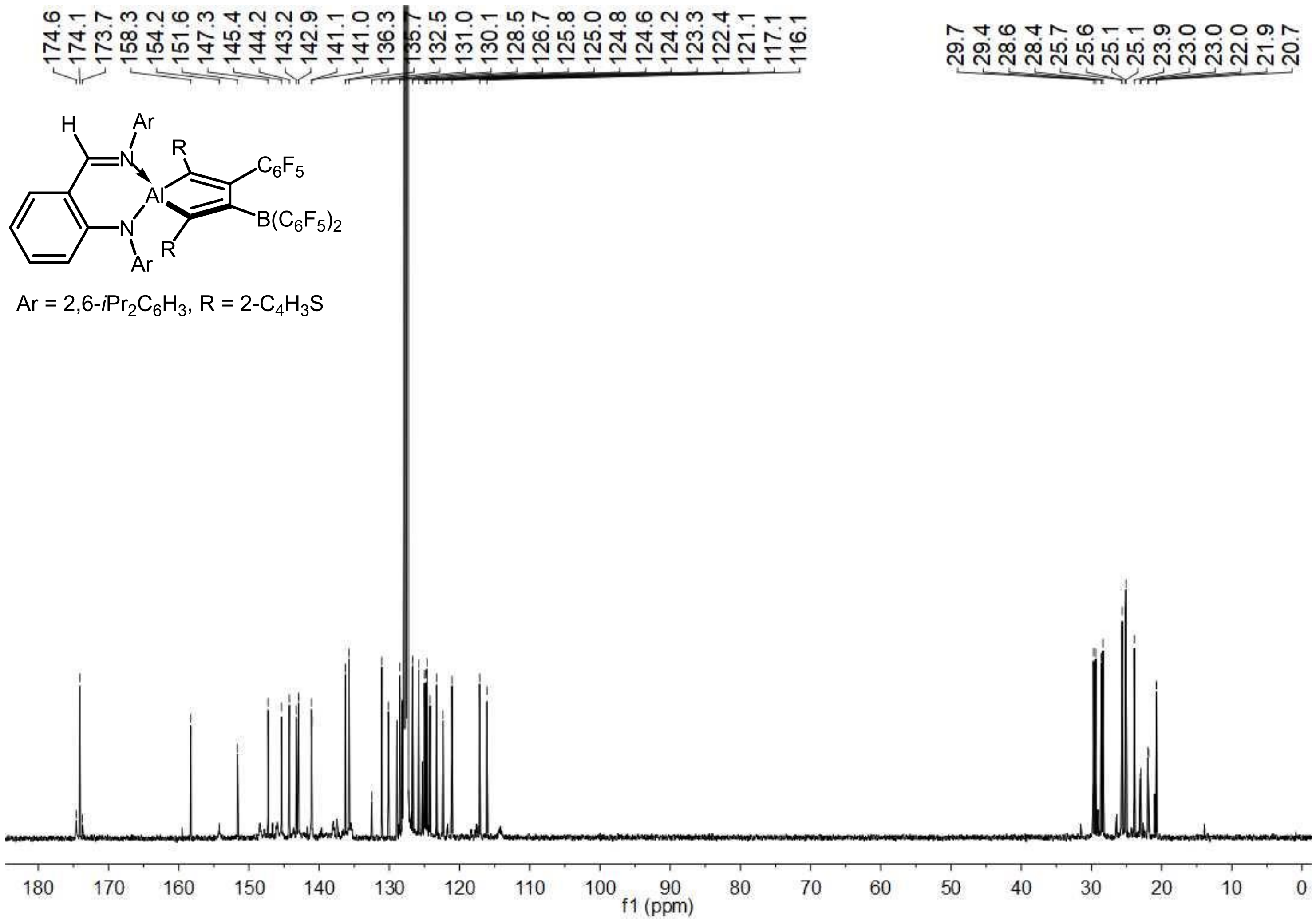

Figure S6-19. ^{13}C NMR spectrum of **6** in C_6D_6

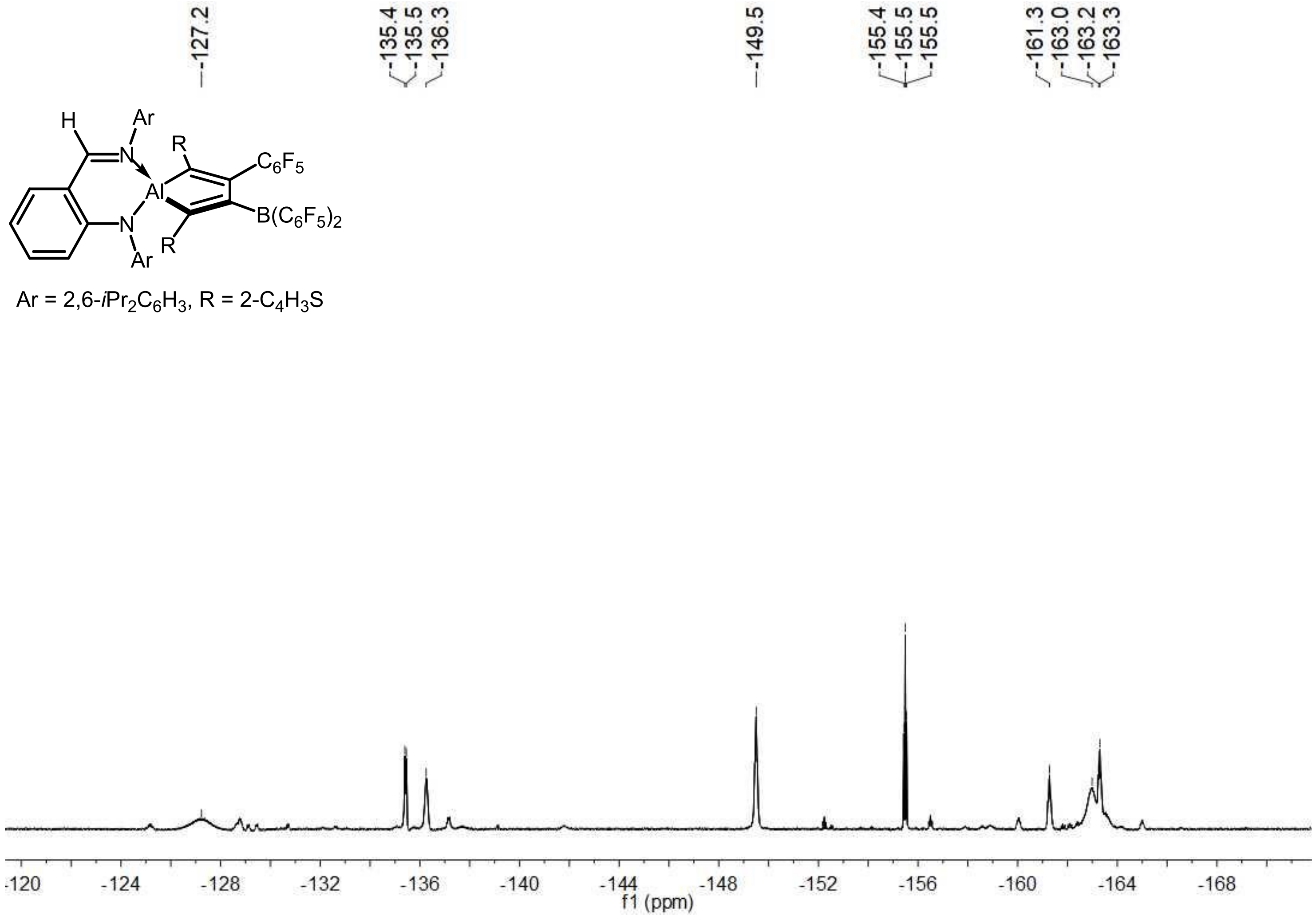

Figure S6-20. ^{19}F NMR spectrum of **6** in C_6D_6

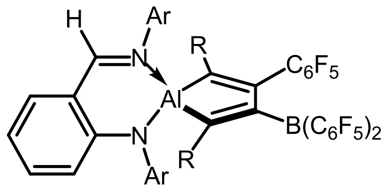

Ar = 2,6-*i*$Pr_2C_6H_3$, R = 2-C_4H_3S

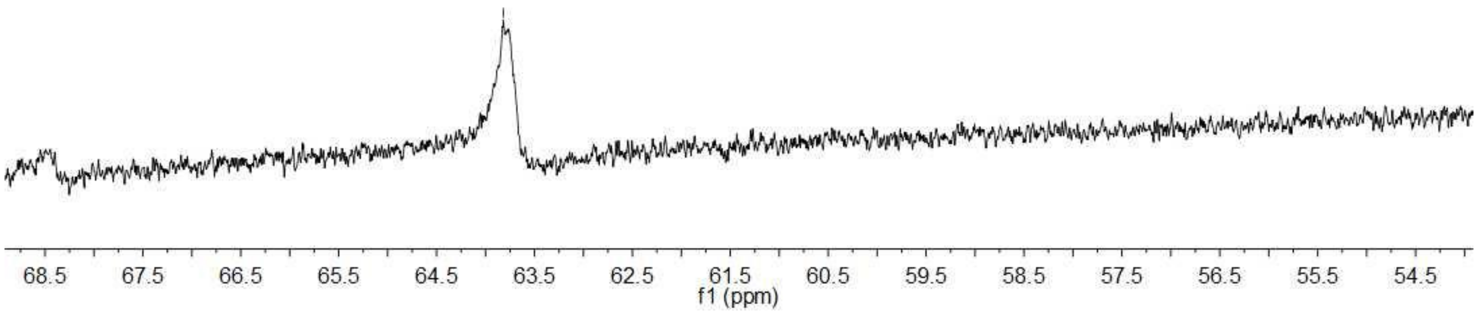

Figure S6-21. ^{11}B NMR spectrum of **6** in C_6D_6

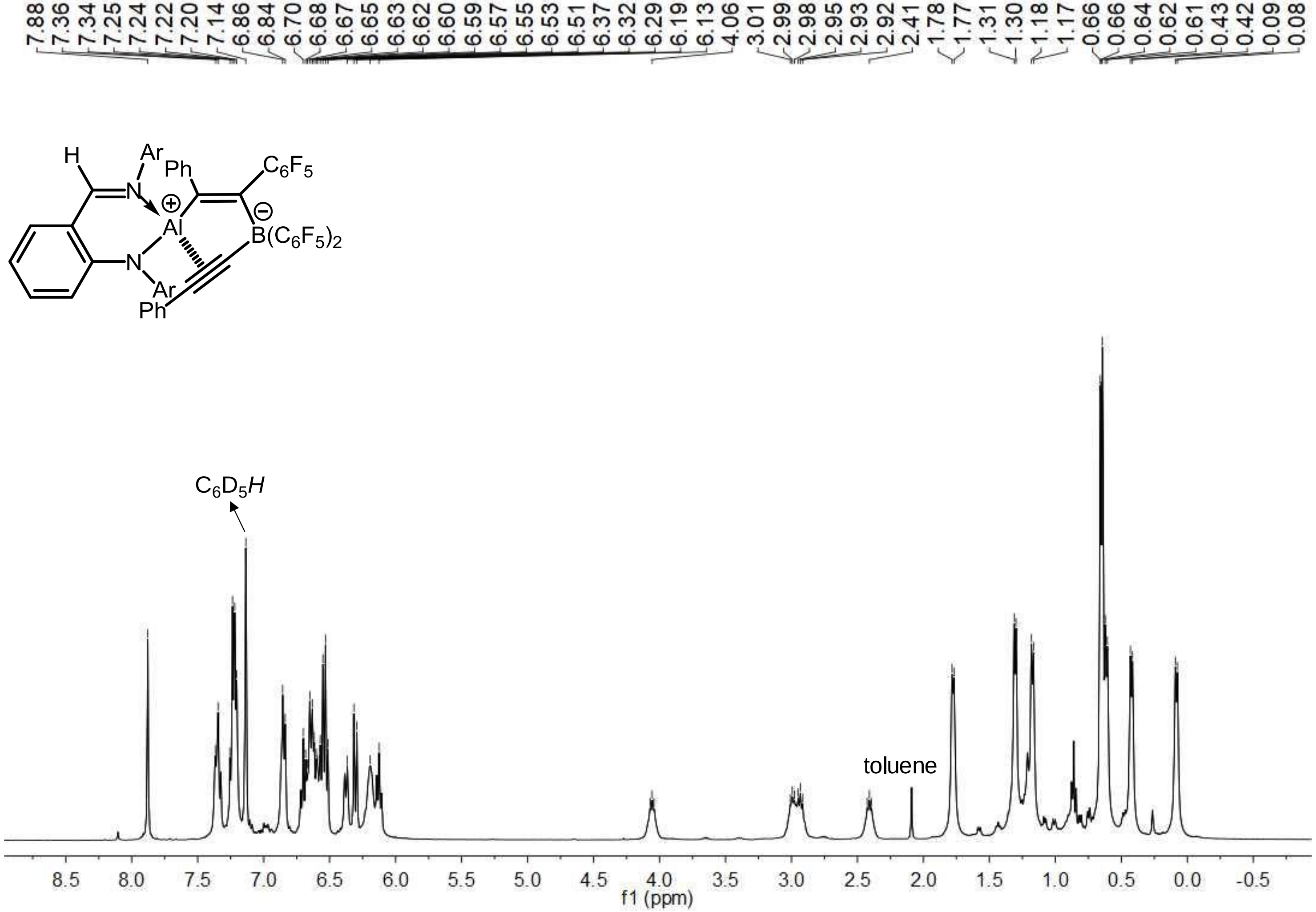

Figure S6-22. ^{1}H NMR spectrum of **7** in C_6D_6

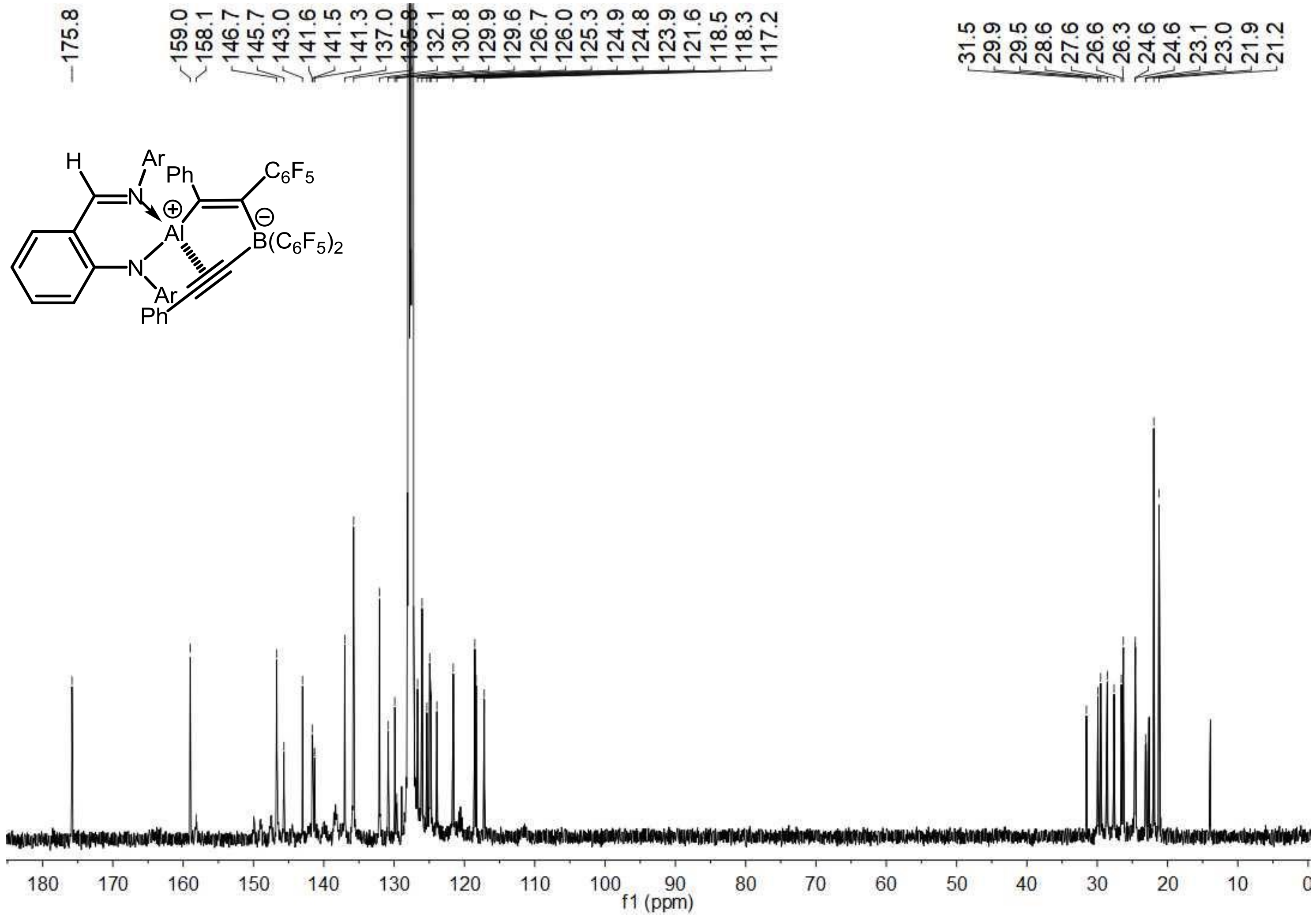

Figure S6-23. ^{13}C NMR spectrum of **7** in C_6D_6

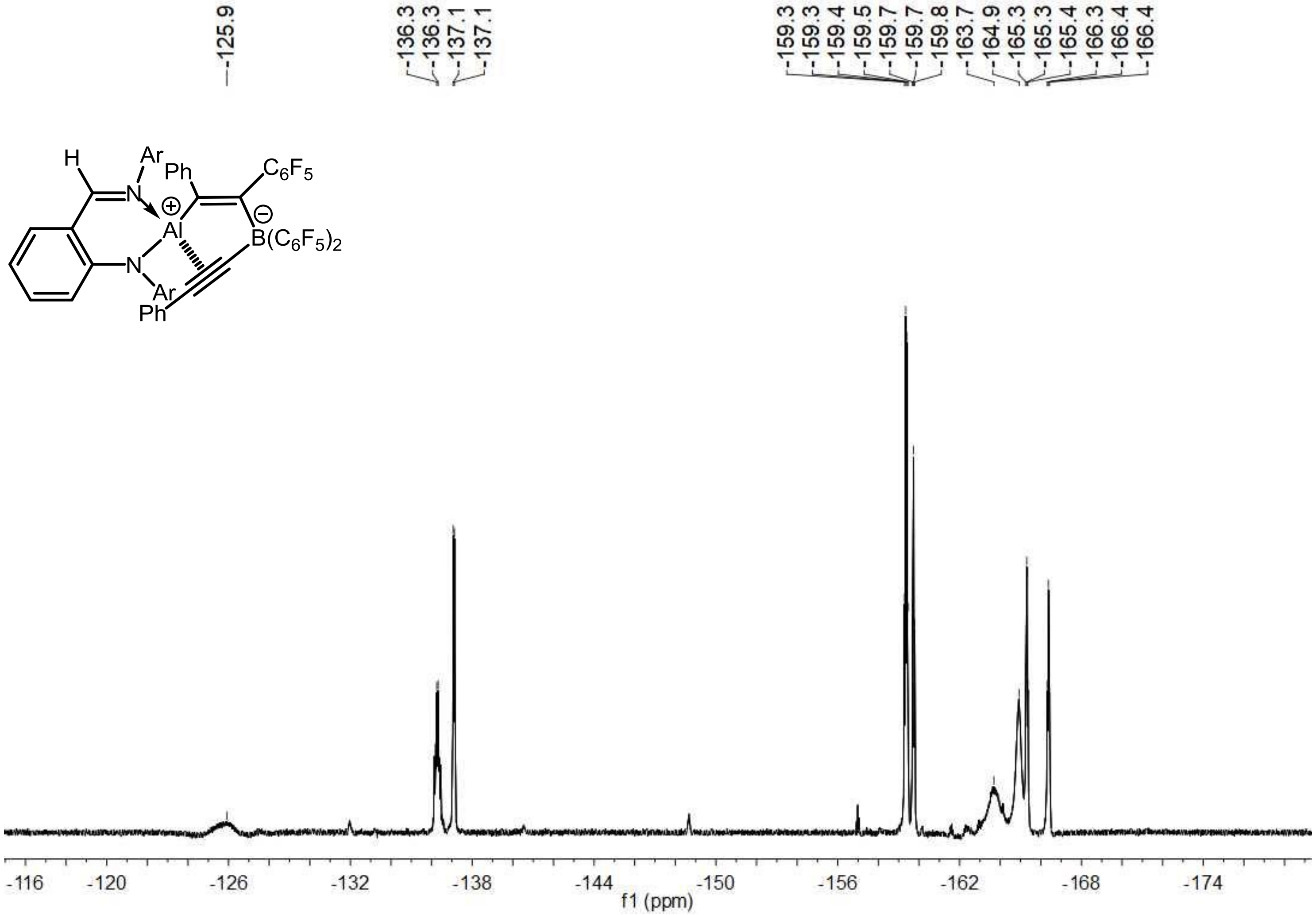

Figure S6-24. ^{19}F NMR spectrum of **7** in C_6D_6

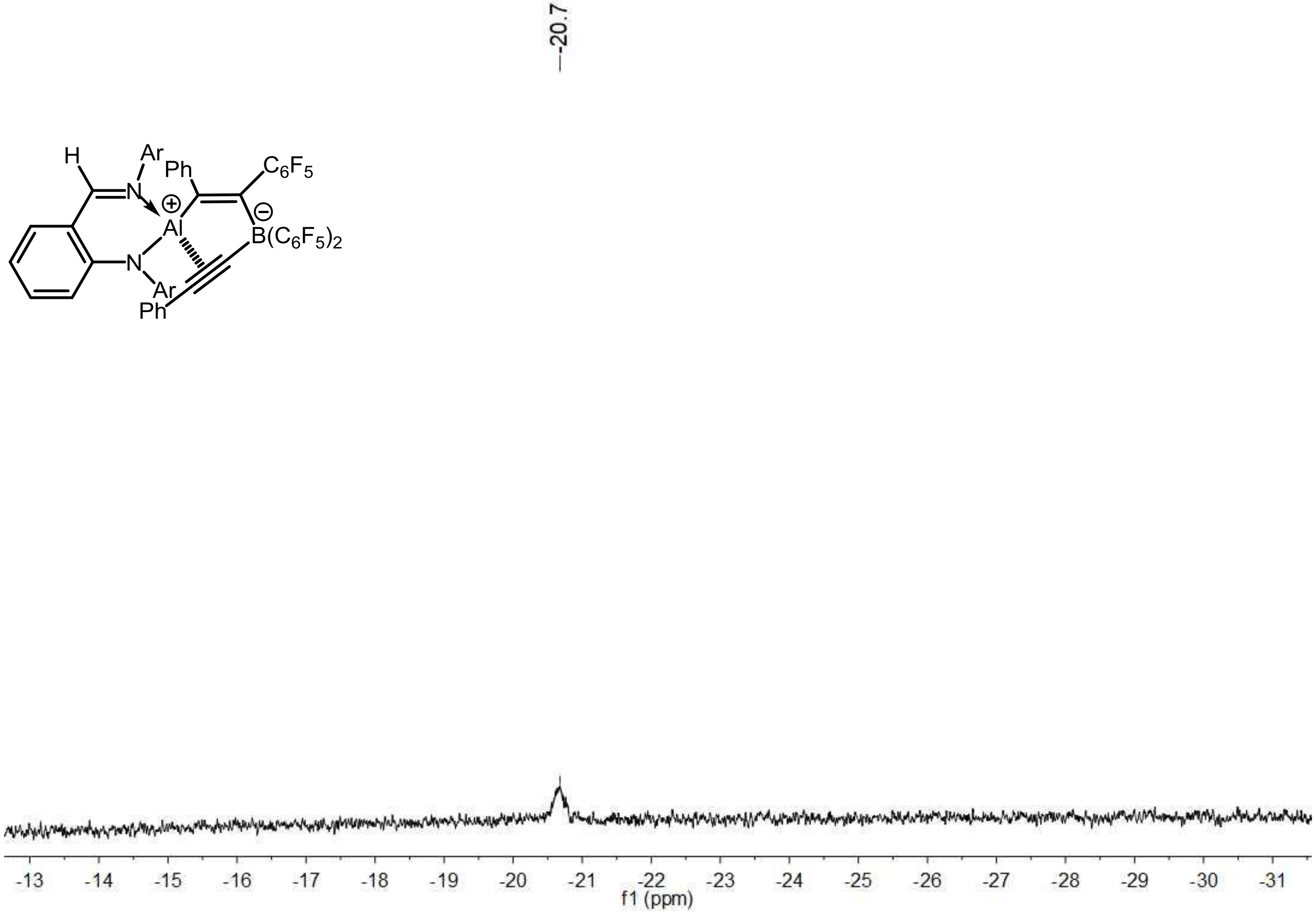

Figure S6-25. ^{11}B NMR spectrum of **7** in C_6D_6

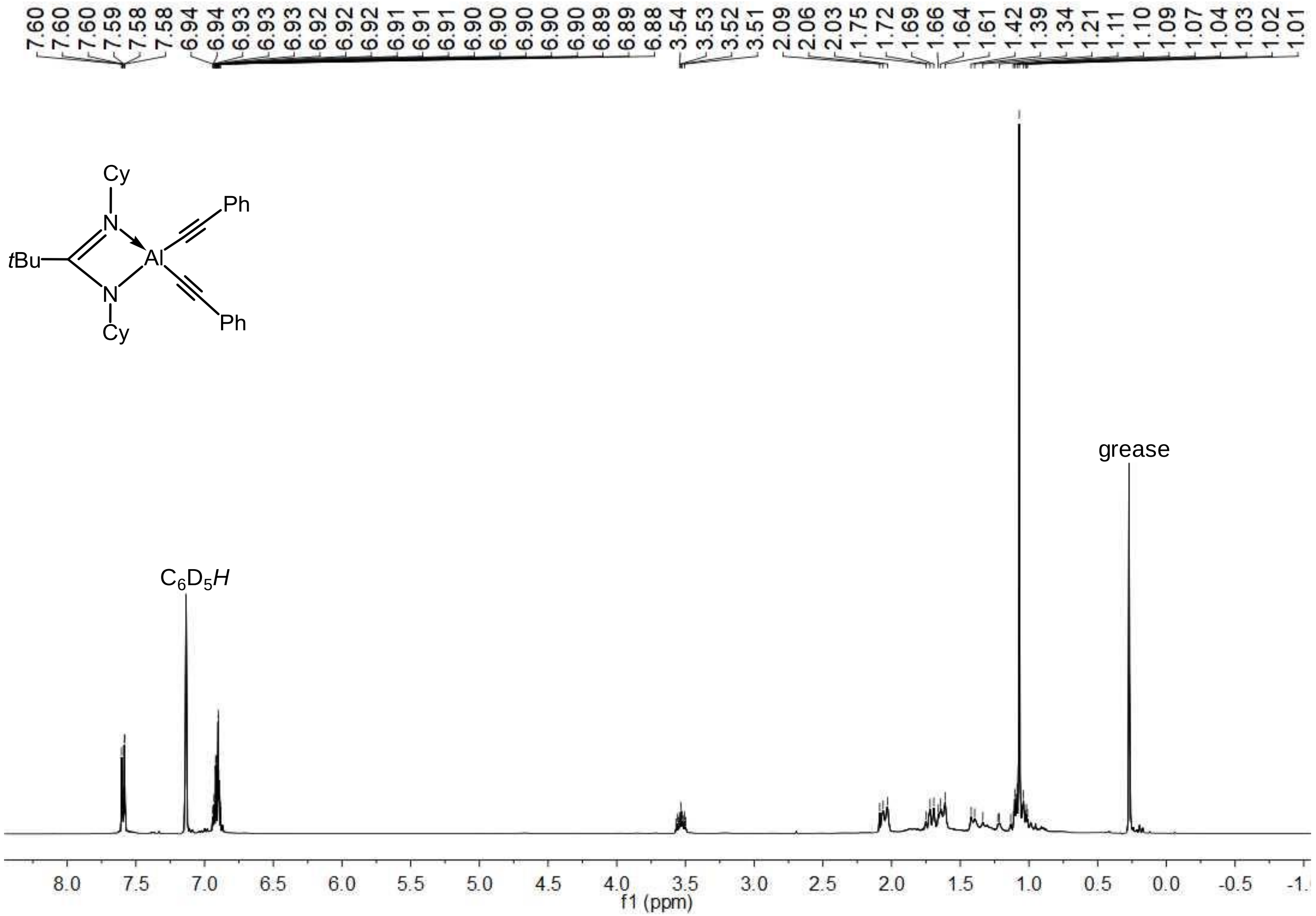

Figure S6-26. ^{1}H NMR spectrum of **8** in C_6D_6

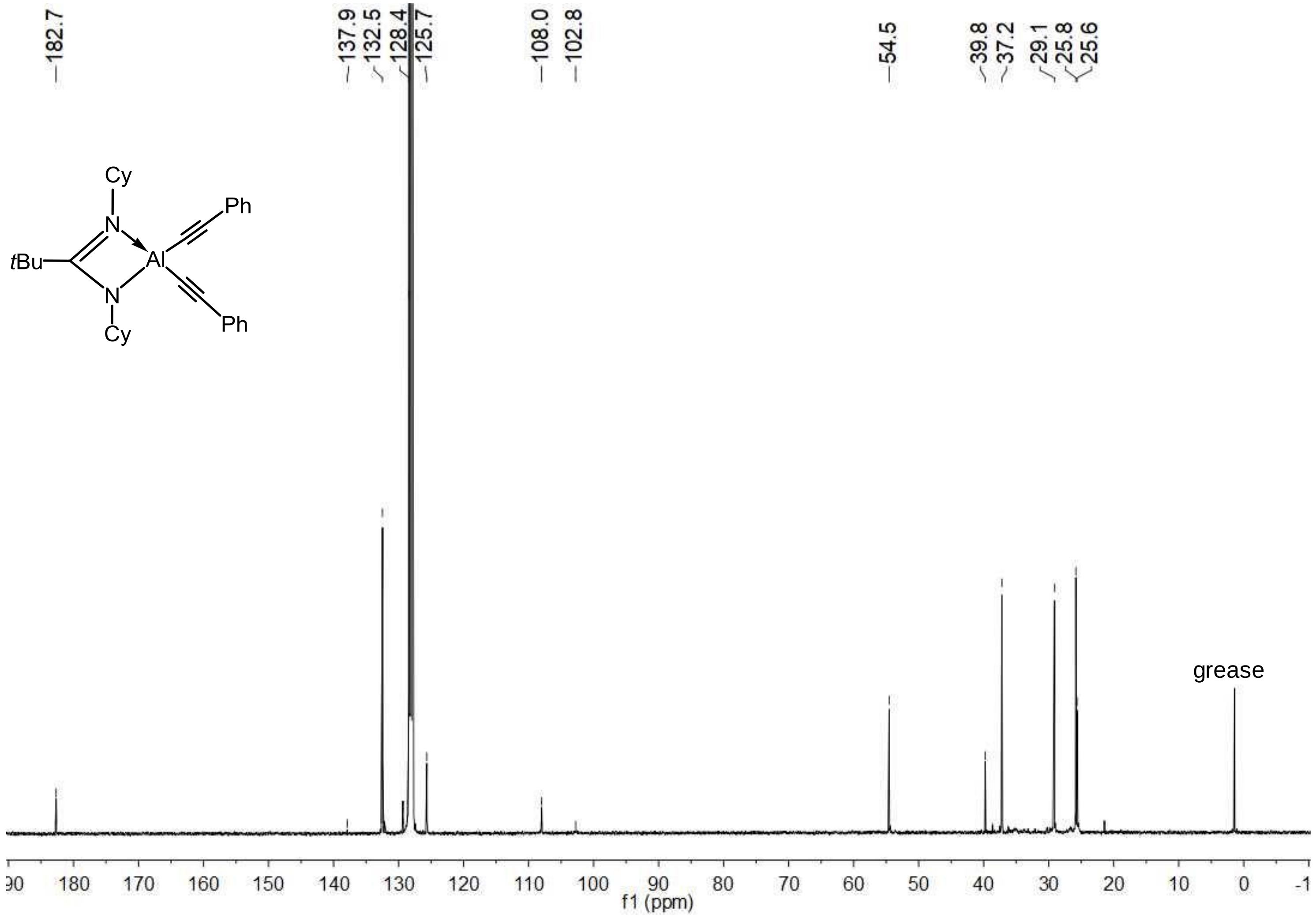

Figure S6-27. ^{13}C NMR spectrum of **8** in C_6D_6

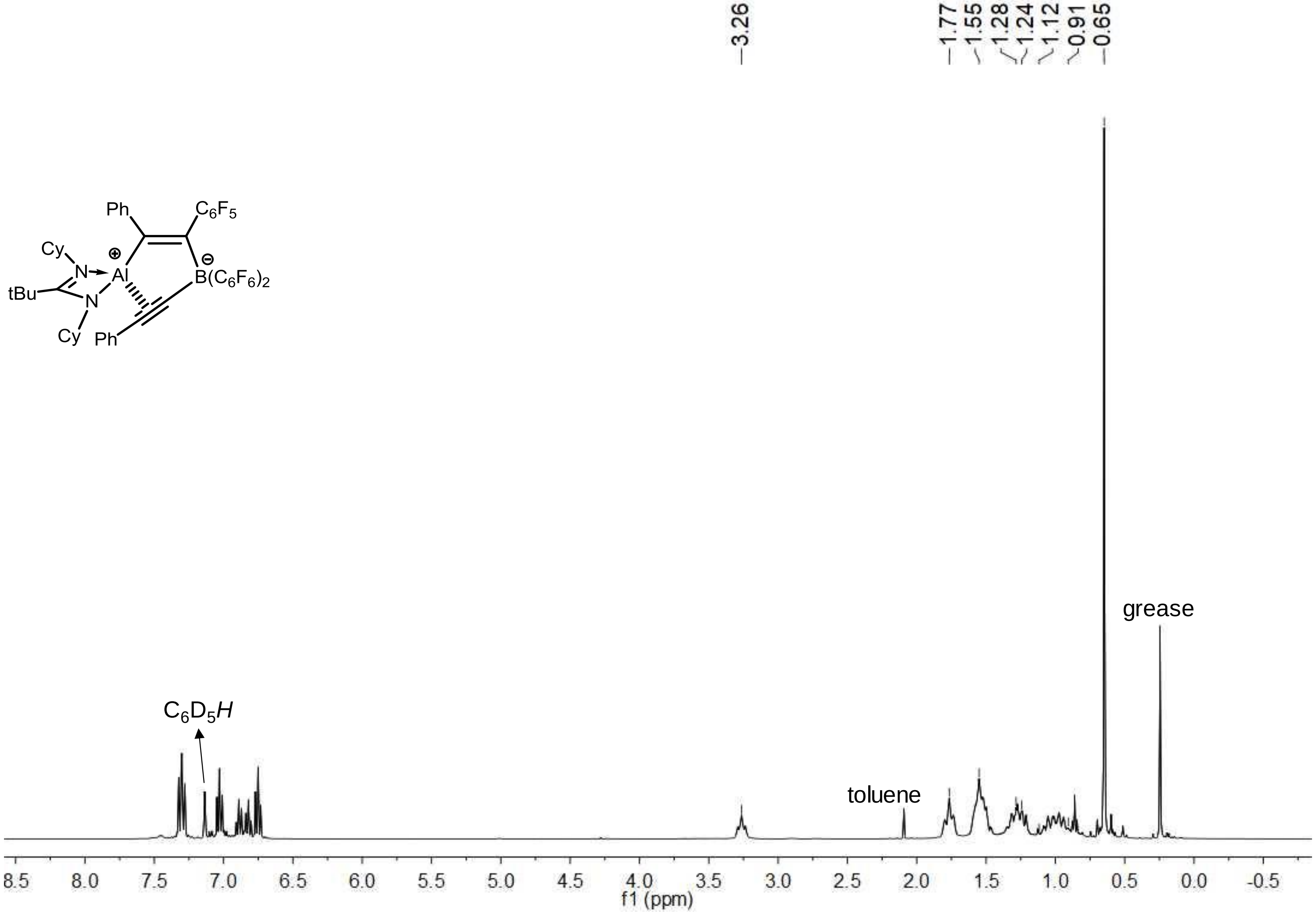

Figure S6-28. ^{1}H NMR spectrum of **9** in C_6D_6

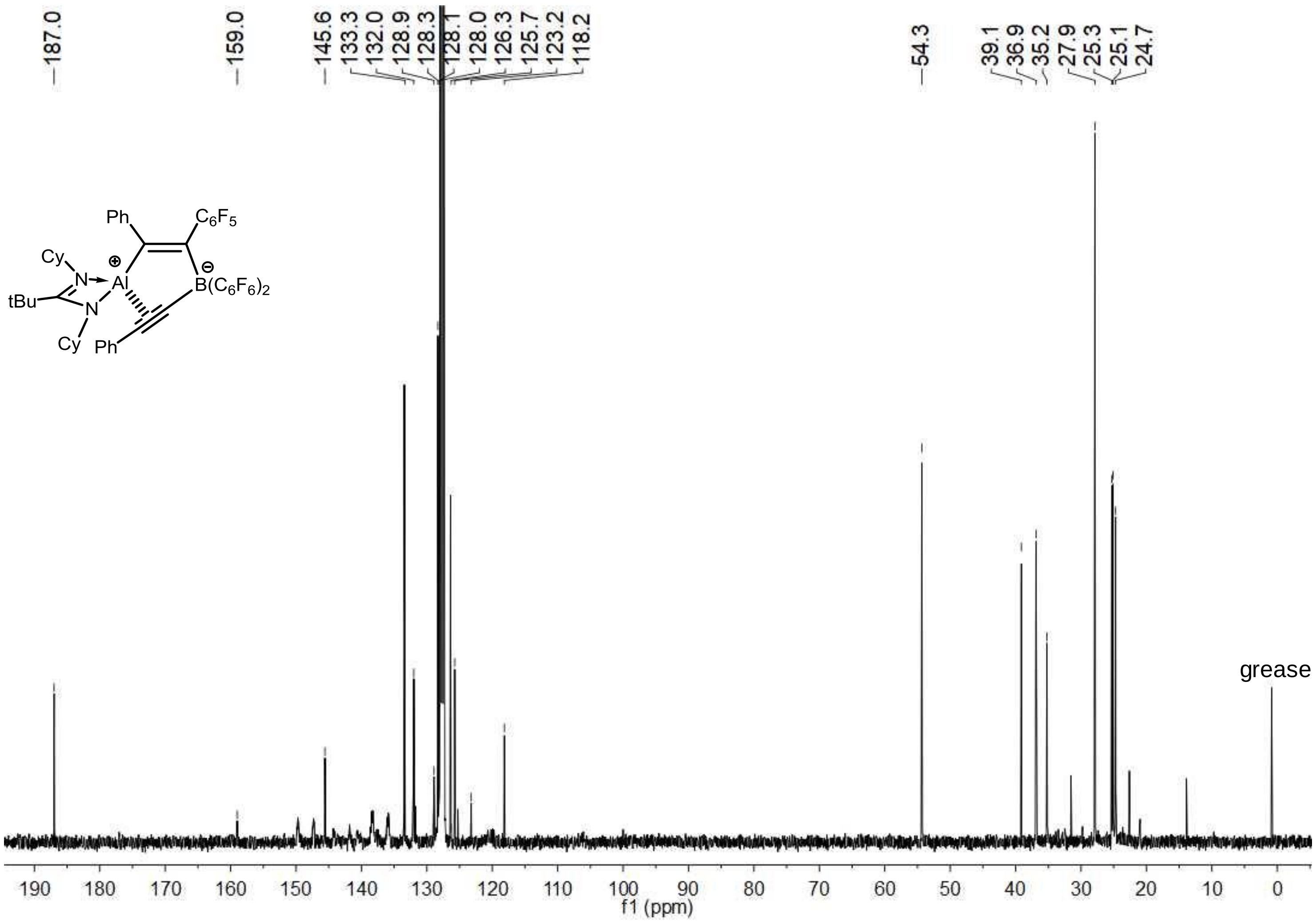

Figure S6-29. ^{13}C NMR spectrum of **9** in C_6D_6

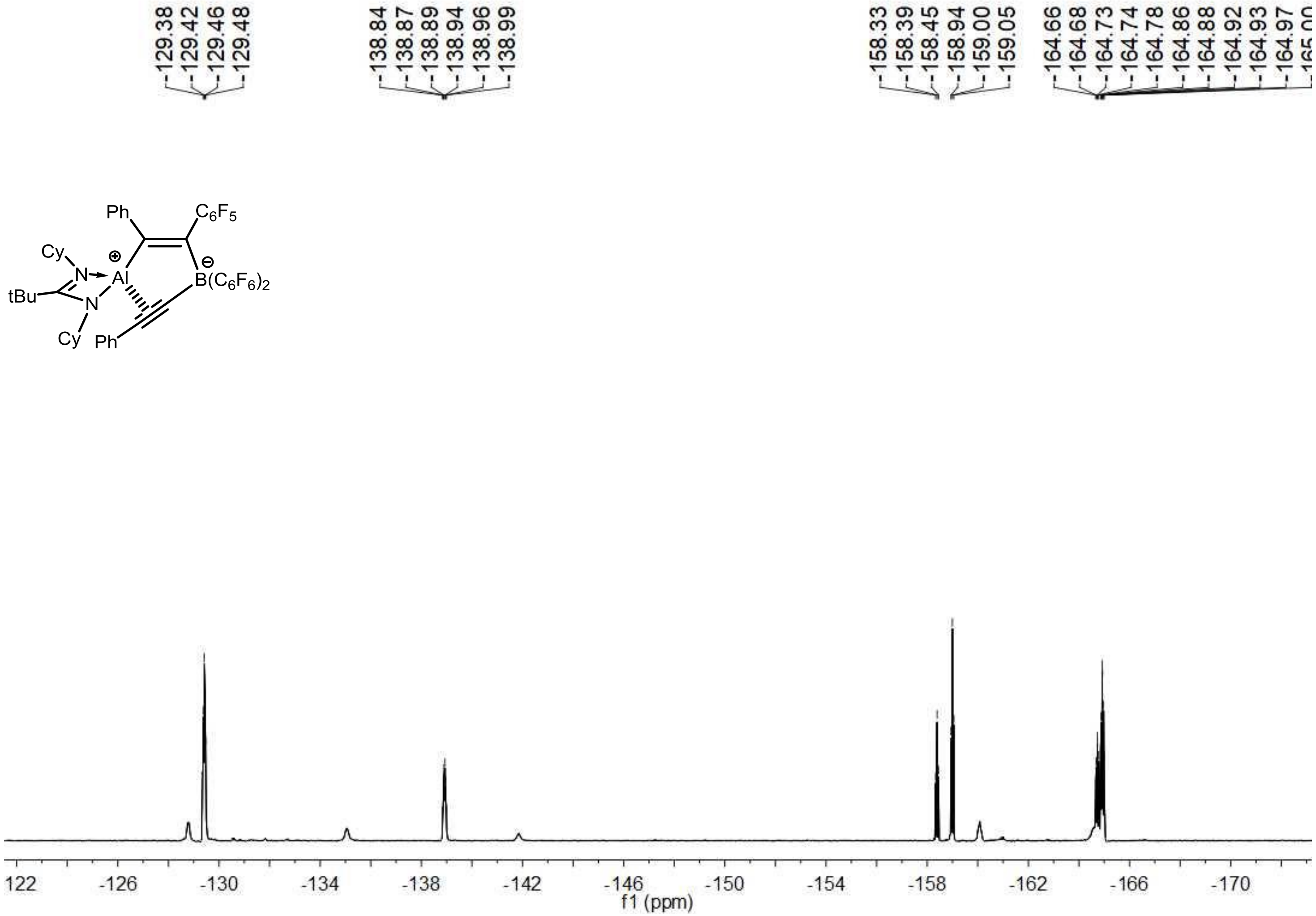

Figure S6-30 ^{19}F NMR spectrum of **9** in C_6D_6

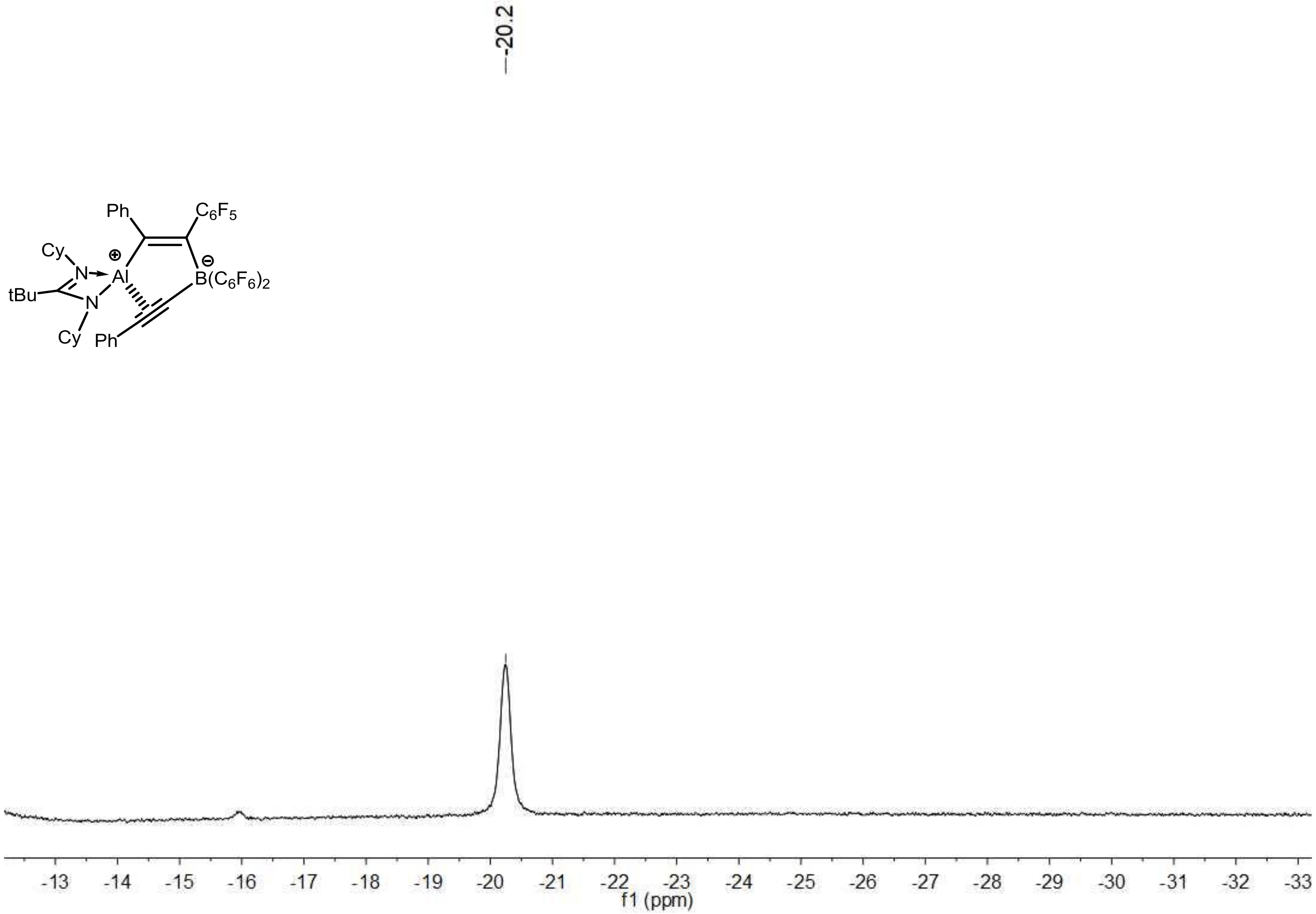

Figure S6-31 ^{11}B NMR spectrum of **9** in C_6D_6

V. References

[S1] W. Zheng, H. W. Roesky, *J. Chem. Soc. Dalton Trans.* **2002**, 2787−2796.

[S2] X. Liu, H. Xia, W. Gao. L. Ye, Y. Mu, Q. Su, Y. Ren. *Eur. J. Inorg. Chem.* **2006**, 1216–1222.

[S3] M. P. Coles, D. C. Swenson, R. F. Jordan, *Organometallics* **1997**, *16*, 5183–5194.

[S4] A. G. Massey, A. J. Park, *J. Organomet. Chem.* **1964**, *2*, 245−250.

[S5] G. M. Sheldrick, *Acta Crystallogr., Sect. A: Found. Crystallogr.* **1990**, *46*, 467–473.

[S6] G. M. Sheldrick, *SHELXL-97, Program for Crystal Structure Refinement*, University of Göttingen, Göttingen, Germany, 1997.

[S7] M. J. Frisch, G. W. T., H. B. Schlegel, G. E. Scuseria, M. A. Robb, J. R. Cheeseman, G. Scalmani, V. Barone, G. A. Petersson, H. Nakatsuji, X. Li, M. Caricato, A. Marenich, J. Bloino, B. G. Janesko, R. Gomperts, B. Mennucci, H. P. Hratchian, J. V. Ortiz, A. F. Izmaylov, J. L. Sonnenberg, D. Williams-Young, F. Ding, F. Lipparini, F. Egidi, J. Goings, B. Peng, A. Petrone, T. Henderson, D. Ranasinghe, V. G. Zakrzewski, J. Gao, N. Rega, G. Zheng, W. Liang, M. Hada, M. Ehara, K. Toyota, R. Fukuda, J. Hasegawa, M. Ishida, T. Nakajima, Y. Honda, O. Kitao, H. Nakai, T. Vreven, K. Throssell, J. A. Montgomery, Jr., J. E. Peralta, F. Ogliaro, M. Bearpark, J. J. Heyd, E. Brothers, K. N. Kudin, V. N. Staroverov, T. Keith, R. Kobayashi, J. Normand, K. Raghavachari, A. Rendell, J. C. Burant, S. S. Iyengar, J. Tomasi, M. Cossi, J. M. Millam, M. Klene, C. Adamo, R. Cammi, J. W. Ochterski, R. L. Martin, K. Morokuma, O. Farkas, J. B. Foresman, and D. J. Fox, *Gaussian 09*. 2009, Wallingford CT: Gaussian, Inc.

[S8] Y. Zhao, D. G. Truhlar, *Theor. Chem. Acc.* **2008**, *120*, 215–241.

[S9] A. V. Marenich, C. J. Cramer, D. G. Truhlar, *J. Phys. Chem. B* **2009**, *113*, 6378–6396.
